# Supplementary material for: PRIMPOL-Mediated Adaptive Response Suppresses Replication Fork Reversal in BRCA-Deficient Cells
Source: Mol Cell. 2020 Feb 6;77(3):461–474.e9. doi: 10.1016/j.molcel.2019.10.008 (PMC7007862; doi:10.1016/j.molcel.2019.10.008)
Supplement: Document S1. Figures S1–S7 and Tables S1–S3 [file mmc1.pdf]

Molecular Cell, Volume *77*

Supplemental Information

PRIMPOL-Mediated Adaptive Response Suppresses Replication Fork Reversal in BRCA-Deficient Cells

Annabel Quinet, Stephanie Tirman, Jessica Jackson, Saša Šviković, Delphine Lemaçon, Denisse Carvajal-Maldonado, Daniel González-Acosta, Alexandre T. Vessoni, Emily Cybulla, Matthew Wood, Steven Tavis, Luis F.Z. Batista, Juan Méndez, Julian E. Sale, and Alessandro Vindigni

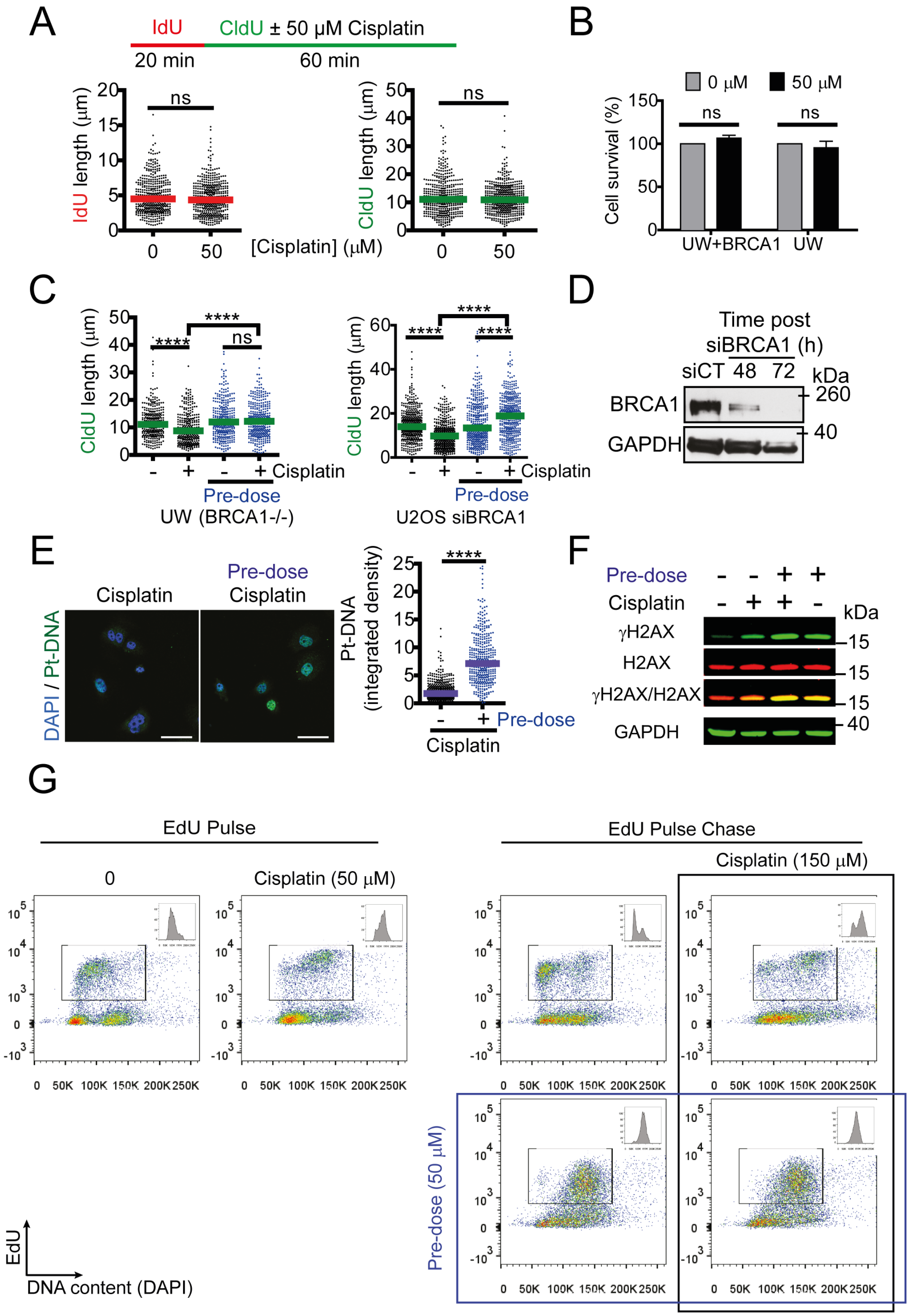

Figure S1. Treatment with a cisplatin pre-dose abolishes nascent DNA degradation in BRCA1-deficient cells, related to Figure 1. (A) Schematic of the single-molecule

DNA fiber tract analysis (top) and size distribution of IdU (bottom left) and CldU (bottom right) tract length in UW cells ± 50 μM cisplatin. Data are pooled from three independent experiments. N ≥ 150 tracts for each data set. Bars represent the median. Statistics: Mann-Whitney; *ns*, non-significant. (B) Cell survival of UW+BRCA1 and UW cells 24 hours after treatment with 50 μM cisplatin (pre-dose). Data represent means ± s.e.m. from three experiments. Statistics: two-way ANOVA followed by Bonferroni test; *ns*, non-significant. (C) Size distribution of CldU tract length in UW cells (left) and U2OS cells depleted for BRCA1 (siBRCA1) (right) ± 150 μM cisplatin with or without the pre-dose. Data are pooled from three independent experiments. N ≥ 150 tracts for each data set. Bars represent the median. Statistics: Mann-Whitney; **** $P < 0.0001$. (D) Expression of BRCA1 48 and 72 hours after siRNA (siBRCA1) knockdown in U2OS cells. (E) Immunofluorescence for cisplatin-modified DNA (Pt-DNA) in UW cells treated with 150 μM cisplatin for 1 hour with or without the pre-dose. Representative images are shown on the left (scale bar: 50 μm). The distribution of the integrated density of cisplatin-modified DNA staining *per* nuclei measured with ImageJ is represented as scatter dot plots (right). Data are pooled from three independent experiments. Bars correspond to median. N ≥ 100 nuclei for each data set. Statistics: Mann-Whitney; **** $P < 0.0001$. (F) Phosphorylated H2AX (γH2AX, green) and total H2AX (red) in UW cells 24 hours after treatment with 150 μM cisplatin with or without the pre-dose. γH2AX/H2AX shows simultaneous detection of both bands. GAPDH was used a loading control. A western blot representative of three independent experiments is shown. (G) UW cells were pulse labeled with EdU for 30 min and immediately fixed 24 hours after treatment with 0 or 50 μM cisplatin (Left, EdU pulse). UW cells were pulse labeled with EdU for 30 minutes immediately before treatment with 150 μM cisplatin (with or without the 50 μM cisplatin pre-dose) and fixed 24 hours later (Right, EdU pulse chase). This pulse chase approach allows evaluation of cell cycle progression of the cells that were replicating at the time of the cisplatin treatment. At the top right of each panel, the cell cycle distribution of EdU-positive cells as determined by DAPI is shown.

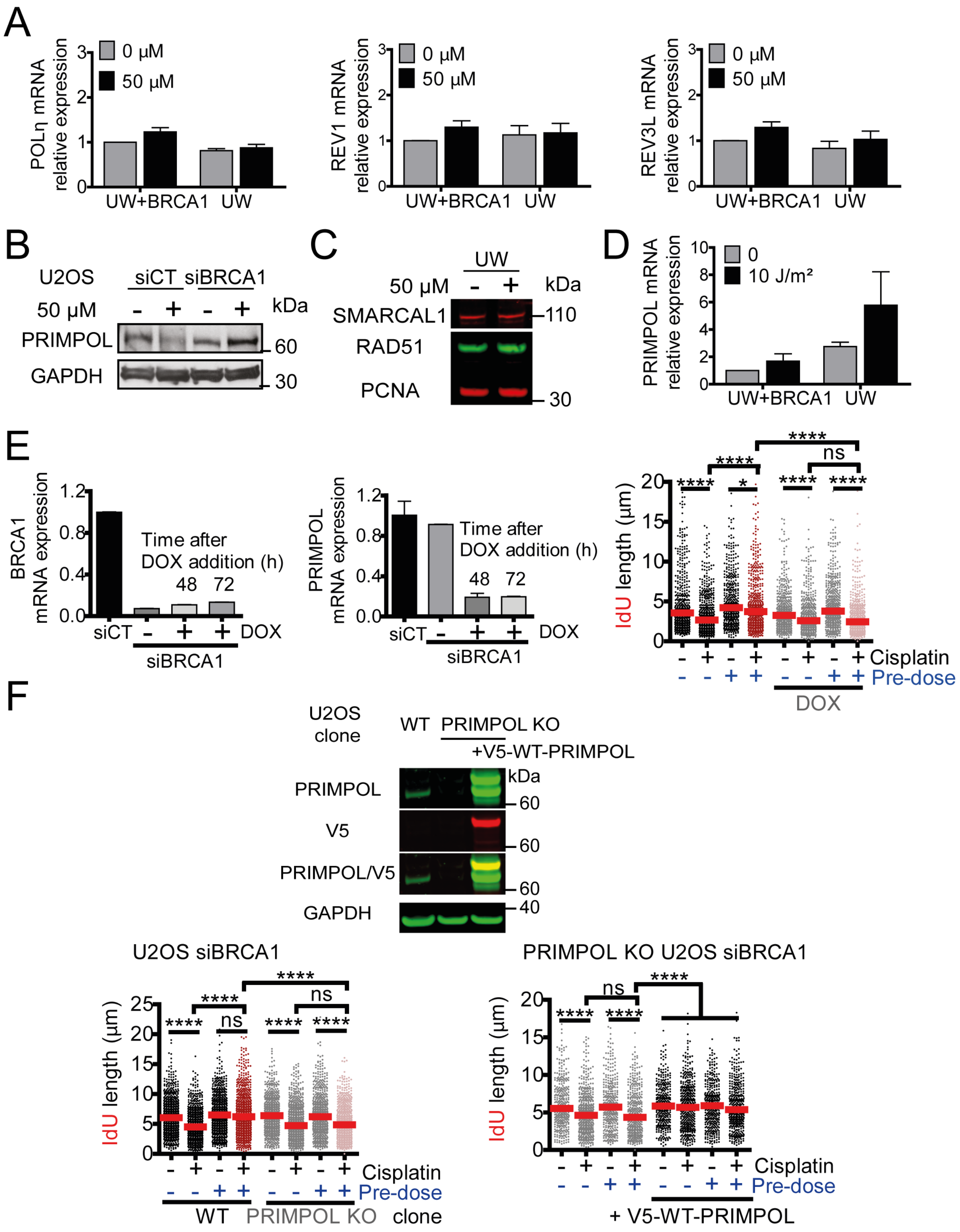

Figure S2. PRIMPOL, but not POLη, REV1 and REV3L, is upregulated in BRCA1-deficient cells, related to Figure 2. (A) TLS polymerases POLη, REV1 and REV3L

(catalytic sub-unit of POLζ) mRNA expression 24 hours after treatment with 0 or 50 μM cisplatin in UW and UW+BRCA1 cells. Data represent means ± s.e.m. from three independent experiments and are presented as relative to untreated UW+BRCA1 cells. (B) PRIMPOL protein expression 24 hours after treatment with 0 or 50 μM cisplatin in U2OS cells depleted for BRCA1 (siBRCA1) or transfected with a control siRNA (siCT). A representative western blot from three independent experiments is shown. GAPDH was used as a loading control. (C) SMARCAL1 and RAD51 protein expression in UW cells 24 hours after treatment with 0 or 50 μM cisplatin. PCNA was used as a loading control. (D) PRIMPOL mRNA expression 24 hours after 0 or 10 J/m^2 UVC in UW and UW+BRCA1 cells. Data represent means ± s.e.m. from three independent experiments and are presented as relative to untreated UW+BRCA1 cells. (E) Validation by RT-qPCR of BRCA1 and PRIMPOL depletion 48 (time of pre-dose) and 72 (time of DNA fiber assay) hours after transfection with siBRCA1 and addition of doxycycline to U2OS cells stably expressing a doxycycline-inducible shPRIMPOL (Left).) Size distribution of IdU tract length in doxycycline-inducible shPRIMPOL U2OS cells depleted for BRCA1 (siBRCA1) ± 150 μM cisplatin with or without the pre-dose, in the presence or absence of doxycycline (DOX) (Right. Data are pooled from three independent experiments. n ≥ 150 tracts for each data set. Bars represent the median. Statistics: Mann-Whitney; **** $P < 0.0001$. (F) PRIMPOL protein expression in PRIMPOL KO and wild-type counterpart U2OS clones and in the PRIMPOL KO clone expressing an exogenous V5-tagged WT-PRIMPOL (PRIMPOL in green, V5 in red, PRIMPOL/V5 shows simultaneous detection of both bands. GAPDH was used a loading control (Top). Size distribution of IdU tract length in PRIMPOL KO and wild-type counterpart U2OS clones depleted for BRCA1 ± 150 μM cisplatin with or without pre-dose (bottom left). Size distribution of IdU tract length in the PRIMPOL KO U2OS clone ± exogenous V5-tagged WT-PRIMPOL ± 150 μM cisplatin with or without pre-dose (bottom right). Data are pooled from three (bottom left) and two (bottom right) independent experiments. N ≥ 150 tracts for each data set. Bars represent the median. Statistics: Mann-Whitney; **** $P < 0.0001$

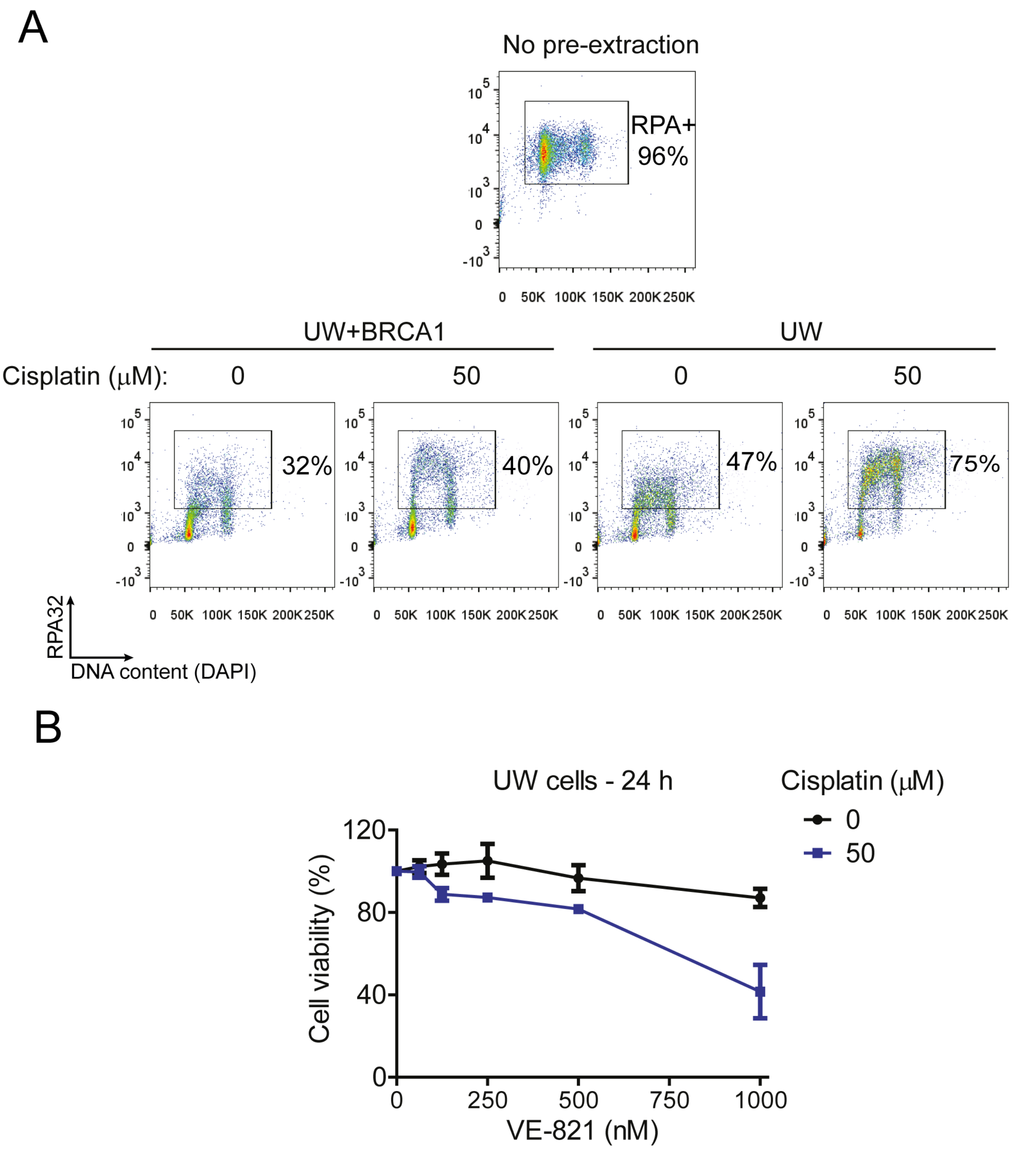

Figure S3. Impact of ATRi on cell survival of UW cells treated with cisplatin, related to Figure 3. (A) Representative experiment showing the detection of chromatin-bound RPA32 as a function of DNA content by flow cytometry in UW+BRCA and UW cells 24 hours after treatment with 50 μM cisplatin. The gate for the positive signal for RPA (RPA+) was defined based on a control sample in which cells were not subjected to pre-extraction prior to fixation and therefore are nearly all positive for RPA staining. Next, the same gate was applied to all the samples in which soluble proteins were pre-extracted before fixation in order to specifically detect cells positive for chromatin-bound RPA. Quantification from

three independent experiments is shown in Figure 3A. (B) Cell survival assessed by XTT of UW cells 24 hours after treatment with 50 μM cisplatin for 1 hour with or without the indicated doses of VE-821. VE-821 was added 1 hour prior to treatment with cisplatin and kept in the media for the following 24 hours. Data are expressed as relative to untreated control (without VE-821) and correspond to mean ± s.e.m. of four independent experiments.

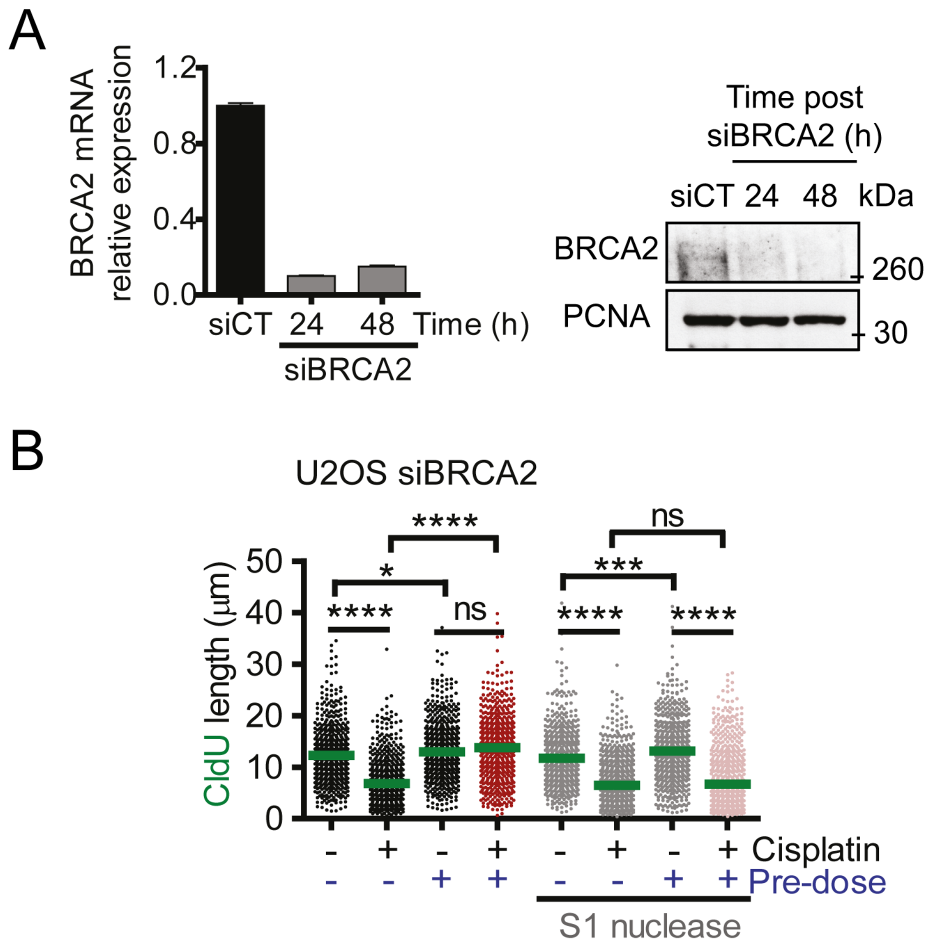

Figure S4. Treatment with a cisplatin pre-dose abolishes nascent DNA degradation and leads to ssDNA gaps in BRCA2-deficient cells, related to Figure 4. (A) Validation of BRCA2 depletion with siRNA (siBRCA2) in U2OS cells by RT-qPCR (left) and Western blot (right) 24 (pre-dose time) and 48 (DNA fiber assay time) hours after transfection. (B) Size distribution of CldU tract length in U2OS cells depleted for BRCA2 ± 150 μM cisplatin with or without the pre-dose followed by treatment with S1 nuclease or not. Data are pooled from three independent experiments. N ≥ 150 tracts for each data set. Bars represent the median. Statistics: Mann-Whitney; **** $P < 0.0001$.

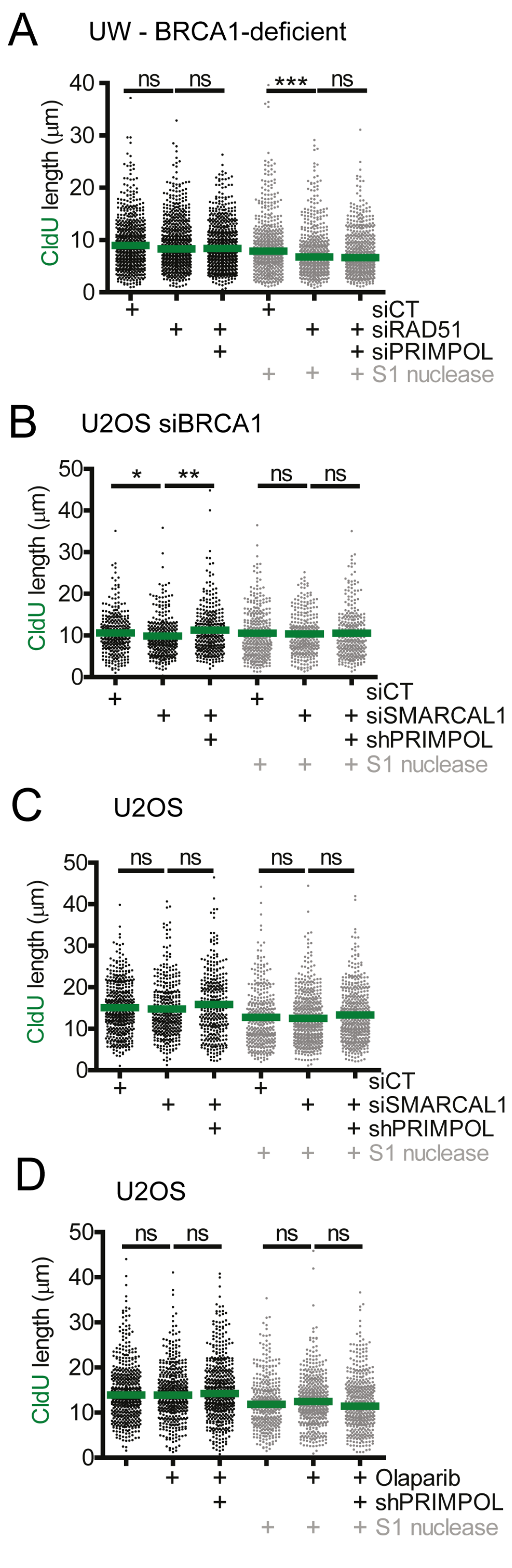

Figure S5. DNA fiber assay data of untreated samples, related to Figure 6. (A) Untreated samples from DNA fiber data shown in Figure 6B. (B) Untreated samples from

DNA fiber data shown in Figure 6C. (C) Untreated samples from DNA fiber data shown in Figure 6D. (D) Untreated samples from DNA fiber data shown in Figure 6E. Statistics: Mann-Whitney; *ns*, non-significant, * $P < 0.05$, ** $P < 0.01$, *** $P < 0.001$.

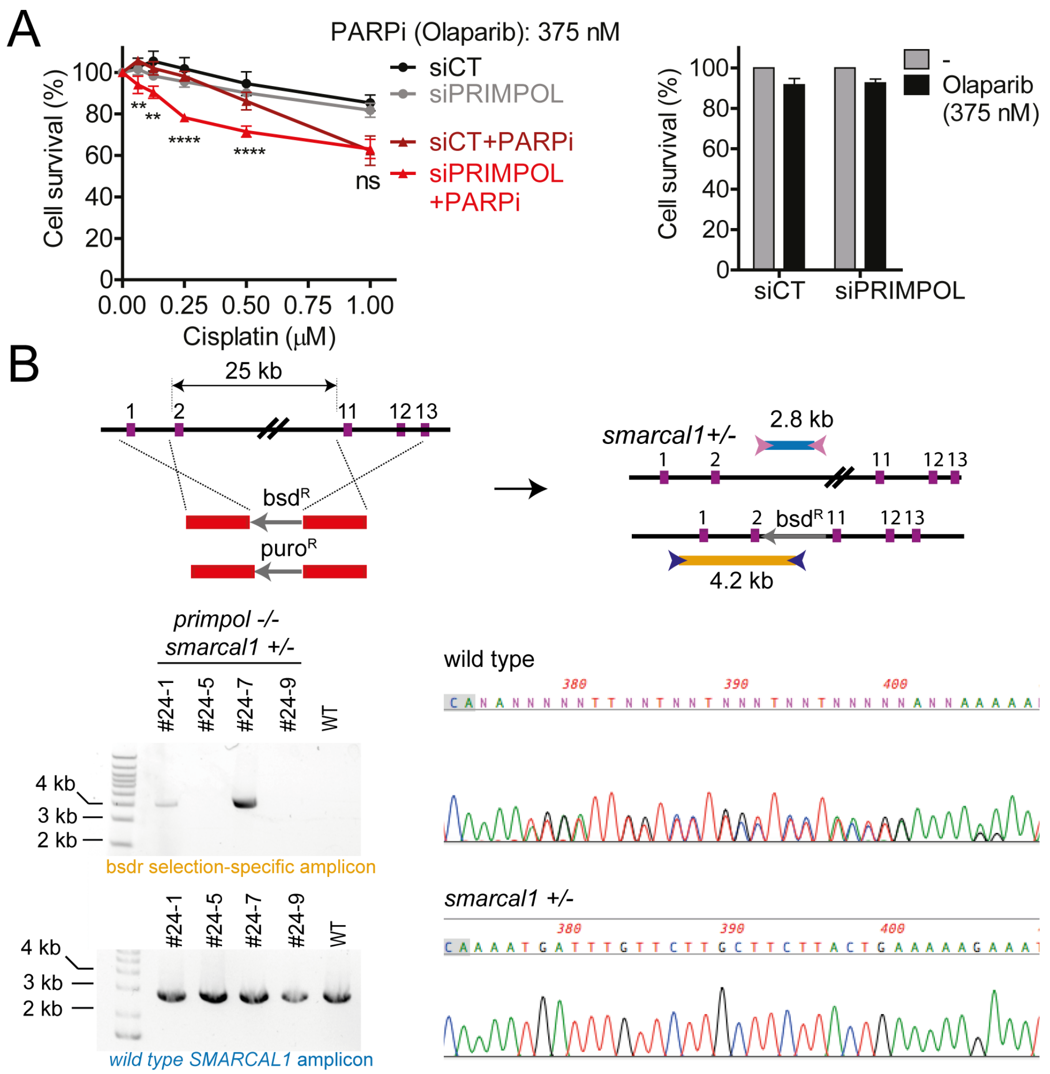

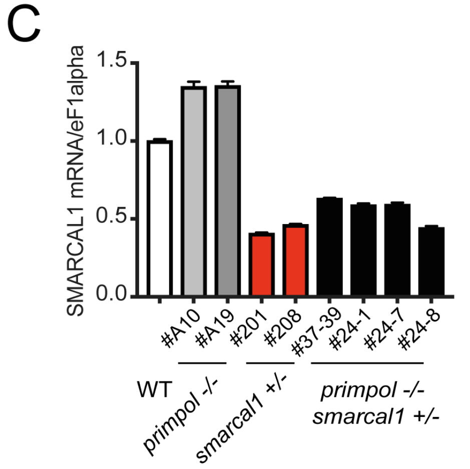

D

cell line	no. passages	doubling time [h] (mean ± SD)
wild type	10	10.81 ± 0.21
	20	11.52 ± 1.40
	40	10.82 ± 1.56
primpol -/-	10	12.89 ± 0.55
	20	13.41 ± 1.74
	40	13.76 ± 3.04
smarcal1 +/-	10	10.93 ± 0.94
	20	11.88 ± 1.83
	40	12.22 ± 3.27
primpol -/- smarcal1+/-	10	19.94 ± 3.91
	20	16.31 ± 5.10
	40	14.24 ± 3.28

Figure S6. Impact of PRIMPOL on cell survival, related to Figure 7. (A) Cell survival of U2OS cells transfected with siCT or siPRIMPOL upon 4 days of chronic treatment with

the indicated doses of cisplatin and PARP inhibitor (PARPi, Olaparib, 375 nM) (left). Cell survival of U2OS cells transfected with siCT or siPRIMPOL 4 days after treatment with Olaparib (375 nM) only (right). Data represent means ± s.e.m. from four independent experiments. Statistics: two-way ANOVA followed by Bonferroni test comparing siCT+PARPi and siPRIMPOL+PARPi (*ns*, non-significant, ** $P < 0.01$, **** $P < 0.0001$). (B) *smarcal1* knockout strategy (Keka et al., 2015) in DT40 *primpol* mutants is depicted on the top, with representative genotyping analysis on the bottom left (bsd^r specific amplicon [orange] to confirm successful targeting and *smarcal1* amplicon [blue] to confirm heterozygosity). The bottom right panel is a Sanger sequencing traces of the polymorphic region located between the homology regions. The upper sequencing trace shows a polymorphic region of *smarcal1,* with the ambiguous peaks reflecting differences between the maternal and paternal alleles of the cell line, as DT40 is derived from an F1 hybrid strain of chicken. The bottom panel is from a *smarcal1+/-* line and has lost the ambiguous peak, indicative of heterozygous deletion. (C) The expression levels of *smarcal1* analyzed by RT-qPCR in several independently derived DT40 clones. Data represent mean ± SD of three qPCR replicates. (D) Measured doubling times of various DT40 mutants at different times relative to transfection with *smarcal1* targeting construct. Mean and standard deviation of three experiments reported. For reference, the original smarcal1-/- cell line (Keka et al., 2015) has a doubling time of 18.2 +/- 3.3 h compared with WT doubling time 11.6 +/- 1.5 h.

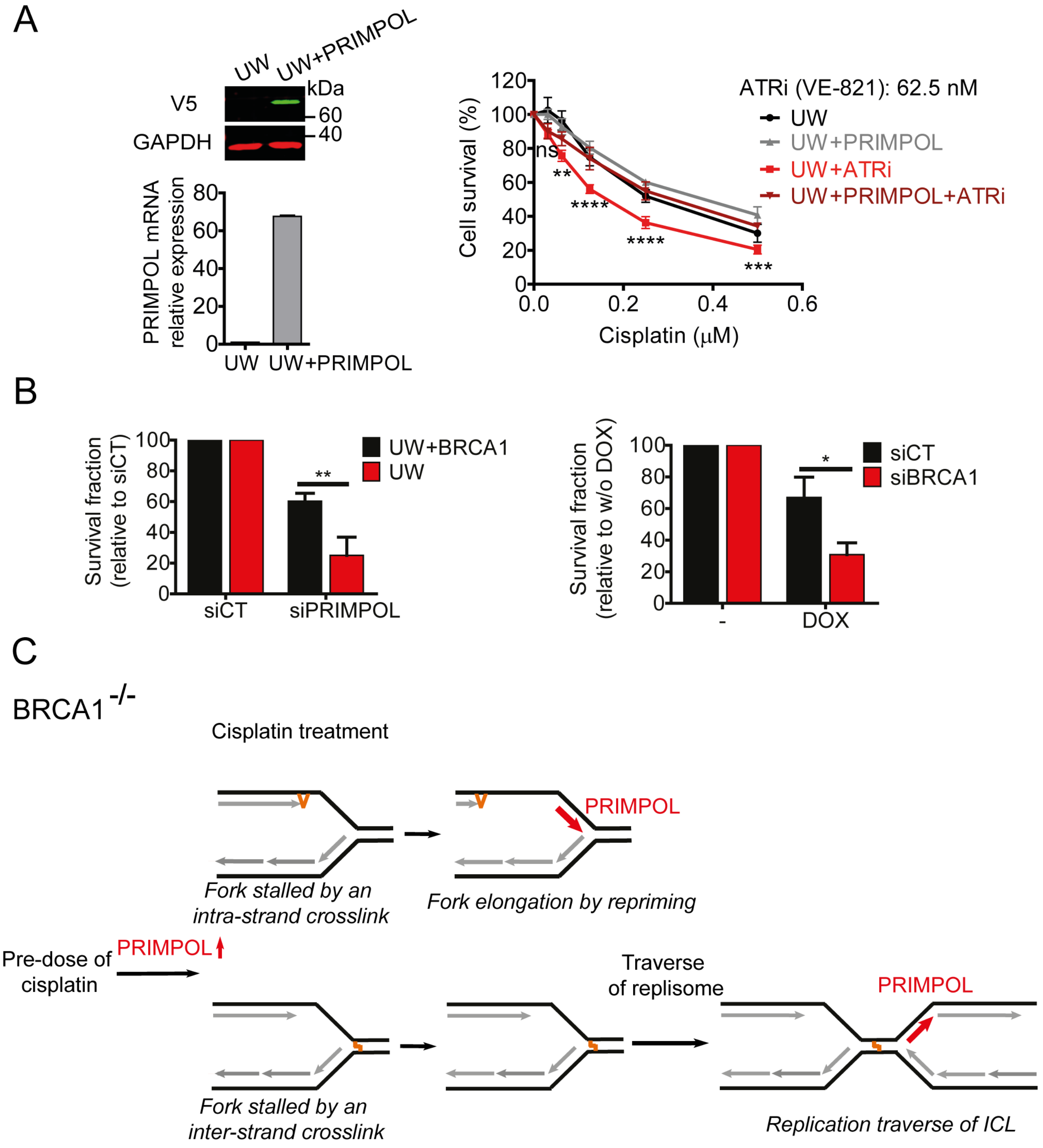

Figure S7: Impact of PRIMPOL on cell survival and proposed model for the role of PRIMPOL in the replication stress response to cisplatin-induced intra- and inter-strand crosslinks, related to Figure 7. (A) Validation of the generation of UW+PRIMPOL cells (UW cells stably overexpressing V5-WT-PRIMPOL) by detection of the V5 tag by western blot (top left) and by PRIMPOL mRNA levels as detected by RT-qPCR (bottom right). Cell survival of UW and UW+PRIMPOL cells 6 days upon chronic

treatment with ATR inhibitor (ATRi, VE-821, 62.5 nM) and the indicated doses of cisplatin (Right). Data represent means ± s.e.m. from three independent experiments. Statistics: two-way ANOVA followed by Bonferroni test comparing UW+ATRi *versus* UW+PRIMPOL+ATRi (*ns*, non-significant, * $P < 0.05$, ** $P < 0.01$, *** $P < 0.001$, **** $P < 0.0001$). (B) Colony forming assay in UW and UW±BRCA1 cells depleted for PRIMPOL (siPRIMPOL) (Left). Colony forming assay in PRIMPOL-depleted (DOX) in U2OS cells depleted for BRCA1 (siBRCA1) or treated with siRNA control (siCT) (Right). Data represent mean ± s.e.m. of three and two independent experiments, respectively. Statistics: two-way ANOVA followed by Bonferroni test comparing BRCA-proficient *versus* BRCA1-deficient. (* $P < 0.05$, ** $P < 0.01$). (C) Proposed model for the role of PRIMPOL in the replication stress response to cisplatin-induced intra- (top) and inter-strand crosslinks (bottom).

A

UW cells				Pre-dose		
% Forks with 1, 2, ≥ 3 internal gaps<br><br>(Total molecules analyzed)	0	Cisplatin	Cisplatin + Mirin	0	Cisplatin	Cisplatin + Mirin
Exp #1	-	12, 5, 0<br><br>(110)	14, 3, 0<br><br>(99)	-	15, 9, 6<br><br>(83)	11, 1, 0<br><br>(97)
Exp #2	11, 1, 0<br><br>(76)	12, 4, 0<br><br>(74)	10, 0, 0<br><br>(76)	13, 0, 0<br><br>(77)	17, 3, 3<br><br>(72)	11, 1, 0<br><br>(74)
Exp #3	-	12, 0, 0<br><br>(95)	9, 3, 1<br><br>(79)	-	21, 3, 3<br><br>(95)	7, 0, 0<br><br>(81)

B

UW cells				Pre-dose		
% Reversed Forks<br><br>(Total molecules analyzed)	0	Cisplatin	Cisplatin + Mirin	0	Cisplatin	Cisplatin + Mirin
Exp #1	-	11<br><br>(110)	18<br><br>(99)	-	7<br><br>(83)	19<br><br>(97)
Exp #2	7<br><br>(76)	9<br><br>(74)	16<br><br>(76)	8<br><br>(77)	8<br><br>(72)	16<br><br>(74)
Exp #3	-	12.6<br><br>(95)	22.8<br><br>(79)	-	12.6<br><br>(95)	19.8<br><br>(81)

Table S1. Electron microscopy data, related to Figures 4 and 5. (A) Percentage of replication forks with 1, 2 or ≥ 3 ssDNA gaps behind forks observed in three independent EM experiments for samples in Figure 4D. (B) Percentage of reversed forks observed in three independent EM experiments for samples in Figure 5B. Number of total molecules analyzed per sample is indicated in brackets.

A

U-2 OS cells		Pre-dose	
% Reversed Forks<br><br>(Total molecules analyzed)	Cisplatin		Cisplatin
Exp #1		24.1<br><br>(54)		15.5<br><br>(71)
Exp #2	5.8<br><br>(87)	18<br><br>(89)	10<br><br>(80)	12.8<br><br>(78)
Exp #3		23.4<br><br>(64)		15.8<br><br>(76)

B

U-2 OS cells			Pre-dose	
% Forks with internal gaps<br><br>(Total molecules analyzed)		Cisplatin		Cisplatin
Exp #1		11.1<br><br>(54)		15.5<br><br>(71)
Exp #2	8.05<br><br>(87)	6.7<br><br>(89)	7.5<br><br>(80)	15.4<br><br>(78)
Exp #3		10.9<br><br>(64)		17.1<br><br>(76)

Table S2. Electron microscopy data, related to Figure 5. (A) Percentage of reversed forks observed in three independent EM experiments for samples in Figure 5C left. (B) Percentage of replication forks with ssDNA gaps behind forks observed in three independent EM experiments for samples in Figure 5C right. Number of total molecules analyzed per sample is indicated in brackets.

A

U-2 OS cells		V5-PRIMPOL
% Reversed Forks<br><br>(Total molecules analyzed)	Cisplatin	Cisplatin
Exp #1	16.5<br><br>(85)	4.1<br><br>(98)
Exp #2	18.6<br><br>(86)	5.3<br><br>(95)
Exp #3	25<br><br>(76)	8.1<br><br>(86)

B

U-2 OS cells		V5-PRIMPOL
% Forks with internal gaps<br><br>(Total molecules analyzed)	Cisplatin	Cisplatin
Exp #1	7.1<br><br>(85)	19.4<br><br>(98)
Exp #2	8.1<br><br>(86)	22.1<br><br>(95)
Exp #3	10.5<br><br>(76)	18.6<br><br>(86)

Table S3. Electron microscopy data, related to Figure 5. (A) Percentage of reversed forks observed in three independent EM experiments for samples in Figure 5C bottom left. (B) Percentage of replication forks with ssDNA gaps behind forks observed in three independent EM experiments for samples in Figure 5C bottom right. Number of total molecules analyzed per sample is indicated in brackets.
