# Supplementary material for: PRIMPOL-Mediated Adaptive Response Suppresses Replication Fork Reversal in BRCA-Deficient Cells
Source: Mol Cell. 2020 Feb 6;77(3):461–474.e9. doi: 10.1016/j.molcel.2019.10.008 (PMC7007862; doi:10.1016/j.molcel.2019.10.008)
Supplement: Document S2. Article plus Supplemental Information [file mmc2.pdf]

Article

Molecular Cell

PRIMPOL-Mediated Adaptive Response Suppresses Replication Fork Reversal in BRCA-Deficient Cells

Graphical Abstract

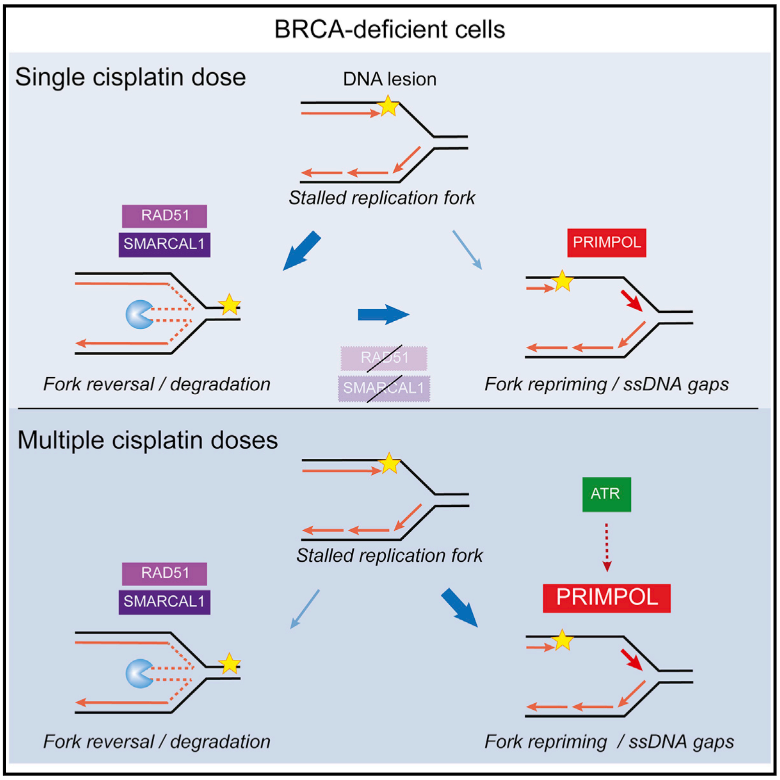

Authors

Annabel Quinet, Stephanie Tirman, Jessica Jackson, ..., Juan Méndez, Julian E. Sale, Alessandro Vindigni

Correspondence

avindigni@wustl.edu

In Brief

Reversed replication forks are degraded in BRCA-deficient cells. Quinet et al. show that treatment with multiple cisplatin doses promotes PRIMPOL repriming while suppressing fork reversal and preventing degradation. These studies identify a new function of PRIMPOL in the replication stress response and explain how cells adapt to multiple drug doses.

Highlights

- Multiple cisplatin doses suppress reversed fork degradation in BRCA-deficient cells
- The PRIMPOL adaptive response suppresses fork reversal and leads to ssDNA gaps
- The ATR kinase regulates the PRIMPOL-mediated adaptive response
- Impaired fork reversal shifts the balance toward PRIMPOL-mediated repriming

Quinet et al., 2020, Molecular Cell *77*, 461–474
February 6, 2020 © 2019 Elsevier Inc.
https://doi.org/10.1016/j.molcel.2019.10.008

Molecular Cell
Article

CellPress

PRIMPOL-Mediated Adaptive Response Suppresses Replication Fork Reversal in BRCA-Deficient Cells

Annabel Quinet,[1] Stephanie Tirman,[1,2] Jessica Jackson,[1] Saša Šviković,[3] Delphine Lemaçon,[2,6] Denisse Carvajal-Maldonado,[2,7] Daniel González-Acosta,[4] Alexandre T. Vessoni,[5] Emily Cybulla,[1,2] Matthew Wood,[1,2] Steven Tavis,[1] Luis F.Z. Batista,[5] Juan Méndez,[4] Julian E. Sale,[3] and Alessandro Vindigni[1,2,8,*]

[1]Division of Oncology, Department of Medicine, Washington University in St. Louis, St. Louis, MO 63110, USA
[2]Edward A. Doisy Department of Biochemistry and Molecular Biology, Saint Louis University School of Medicine, St. Louis, MO 63104, USA
[3]Division of Protein & Nucleic Acid Chemistry, Medical Research Council Laboratory of Molecular Biology, Francis Crick Avenue, Cambridge CB2 0QH, UK
[4]Spanish National Cancer Research Centre (CNIO), Madrid 28029, Spain
[5]Division of Hematology, Department of Medicine, Washington University School of Medicine, St. Louis, MO 63110, USA
[6]Present address: Department of Cell Biology and Physiology, Washington University School of Medicine, St. Louis, MO 63110, USA
[7]Present address: Department of Epigenetics and Molecular Carcinogenesis, The University of Texas MD Anderson Cancer Center, Smithville, TX 78957, USA
[8]Lead Contact
*Correspondence: avindigni@wustl.edu
https://doi.org/10.1016/j.molcel.2019.10.008

SUMMARY

Acute treatment with replication-stalling chemotherapeutics causes reversal of replication forks. BRCA proteins protect reversed forks from nucleolytic degradation, and their loss leads to chemosensitivity. Here, we show that fork degradation is no longer detectable in BRCA1-deficient cancer cells exposed to multiple cisplatin doses, mimicking a clinical treatment regimen. This effect depends on increased expression and chromatin loading of PRIMPOL and is regulated by ATR activity. Electron microscopy and single-molecule DNA fiber analyses reveal that PRIMPOL rescues fork degradation by reinitiating DNA synthesis past DNA lesions. PRIMPOL repriming leads to accumulation of ssDNA gaps while suppressing fork reversal. We propose that cells adapt to repeated cisplatin doses by activating PRIMPOL repriming under conditions that would otherwise promote pathological reversed fork degradation. This effect is generalizable to other conditions of impaired fork reversal (e.g., SMARCAL1 loss or PARP inhibition) and suggests a new strategy to modulate cisplatin chemosensitivity by targeting the PRIMPOL pathway.

INTRODUCTION

Germline mutations of the breast cancer susceptibility genes 1 and 2 (*BRCA1/2*) account for the majority of familial breast and ovarian cancers (Antoniou et al., 2003; Ford et al., 1998; King et al., 2003; Metcalfe et al., 2010; Nielsen et al., 2016). Aside from their well-established roles in double-strand break repair, BRCA proteins protect reversed replication forks from nucleolytic degradation (Kolinjivadi et al., 2017; Lemaçon et al., 2017; Mijic et al., 2017; Schlacher et al., 2011, 2012; Taglialatela et al., 2017; Ying et al., 2012). While replication fork reversal is generally seen as an important mechanism that allows replication forks to reverse their course to aid repair or bypass of DNA damage (Higgins et al., 1976; Neelsen and Lopes, 2015), it can also lead to the pathological degradation of replication intermediates when reversed forks are not adequately protected by the BRCA proteins. In the absence of BRCA proteins, the extensive degradation of reversed replication forks has been linked to increased genomic instability and chemotherapeutic sensitivity (Quinet et al., 2017b; Ray Chaudhuri et al., 2016; Schlacher et al., 2011). However, the relative contributions of the homologous recombination and fork protection functions of BRCA1/2 to the maintenance of genomic stability are still under investigation (Daza-Martin et al., 2019; Feng and Jasin, 2017; Ray Chaudhuri et al., 2016).

Besides replication fork reversal, cells have alternative DNA-damage tolerance (DDT) mechanisms to ensure that replication continues with minimal effects on fork elongation. For example, fork progression is facilitated by specialized translesion synthesis (TLS) polymerases that are able to replicate through a damaged DNA template, albeit with lower fidelity (Chatterjee and Walker, 2017; Sale, 2013; Vaisman and Woodgate, 2017). Alternatively, the replisome may skip the damaged DNA, thus leaving an unreplicated single-stranded DNA (ssDNA) gap to be repaired after replication. The bacterial replisome is able to reinitiate DNA synthesis downstream of a leading-strand lesion by *de novo* priming and recycling or exchange of stalled replicative polymerases (Heller and Marians, 2006). This mechanism also appears to efficiently restart replication in vertebrates using the PRIMPOL protein (Bianchi et al., 2013; García-Gómez et al., 2013; Keen et al.,

Check for updates

2014a; Kobayashi et al., 2016; Mourón et al., 2013; Pilzecker et al., 2016; Schiavone et al., 2016; Šviković et al., 2018; Wan et al., 2013). PRIMPOL has a conserved motif present in the archaeo-eukaryotic primases (AEP), and its primase activity allows *de novo* DNA priming (or repriming) downstream of the blocking lesion (García-Gómez et al., 2013; Mourón et al., 2013). How cells choose between fork reversal, TLS, or repriming is largely unknown. Interestingly, repriming mechanisms at stalled forks limit extensive fork uncoupling and fork reversal in *Saccharomyces cerevisiae*, suggesting that these mechanisms are mutually exclusive (Fumasoni et al., 2015). Whether this is the case in human cells remains undiscovered, and the molecular steps involved in the choice between these mechanisms need to be defined.

To date, most studies focus on the effect of genotoxic agents on replication fork stability immediately after drug treatment. In particular, the discovery that BRCA proteins protect reversed forks from pathological nucleolytic degradation came from single-molecule DNA fiber experiments performed immediately or shortly after exposure to replication-stalling agents (Schlacher et al., 2011, 2012; Ying et al., 2012). However, mammalian cells can adapt to genotoxic stress and respond differently to a new challenge (Quinet et al., 2018). For example, early studies showed that monkey or human cells exposed to low doses of ultraviolet (UV) or ionizing radiation, respectively, were better able to cope with subsequent treatment with a higher radiation dose (Olivieri et al., 1984; Sarasin and Hanawalt, 1978). More recently, exposure of primary human cells to UV light was shown to cause the upregulation of the TLS polymerase Pol η, prompting cells to better tolerate replication stress from follow-up exposure to higher UV doses (Lerner et al., 2017). Importantly, studying how cells adapt to multiple drug doses becomes particularly relevant in the context of cancer treatment regimens involving multiple doses of chemotherapeutics.

In this study, we investigated how replication is perturbed in BRCA1-deficient cancer cells treated with multiple doses of cisplatin, a crosslinking agent frequently used to treat ovarian cancers (Helm and States, 2009). We found that treatment with multiple doses of cisplatin abolishes the widely described nascent DNA degradation phenotype of BRCA1-deficient cells. This effect is due to the upregulation and increased chromatin recruitment of the PRIMPOL protein. Using a combination of genome-wide, single-molecule DNA fiber and electron microscopy (EM) approaches, we demonstrate that PRIMPOL rescues fork degradation through its *de novo* priming activity and leads to accumulation of internal ssDNA gaps behind the forks. These studies suggest that the balance between fork reversal and repriming is tilted toward repriming in genetic backgrounds that lead to extensive reversed fork degradation. We also found that loss of fork reversal factors promotes PRIMPOL repriming in both BRCA1-deficient and -proficient cells, indicating that cellular reliance on fork repriming is more broadly enhanced under conditions of impaired fork reversal. Collectively, our results establish a new paradigm for the PRIMPOL protein in replication fork protection and revisit current models for how BRCA1-deficient cancer cells cope with cisplatin-induced lesions.

RESULTS

Treatment with a Cisplatin Pre-dose Prevents Nascent DNA Degradation in BRCA1-Deficient Cells

Here, we sought to investigate how replication is perturbed in BRCA1-deficient cells after treatment with multiple cisplatin doses, as usually applied in a typical course of platinum-based chemotherapy (Taniguchi et al., 2003). We used the BRCA1 null human ovarian cancer cell line UWB1.289 (named UW here) and its complemented derivative UW+BRCA1 (DelloRusso et al., 2007), plus the human osteoscarcoma U2OS cells, which were siRNA depleted for *BRCA1*. We first confirmed that UW cells were more sensitive to cisplatin than wild-type cells (Figure 1A) (Lohse et al., 2015) and that treatment with a single cisplatin dose leads to fork degradation in a BRCA-deficient background (Lemaçon et al., 2017). Nucleolytic degradation following replication fork stalling was monitored by single-molecule DNA fiber assays by pulse-labeling cells with the first thymidine analog IdU (red) for 20 min, followed by treatment with 150 μM cisplatin and concomitant labeling with the second thymidine analog CldU (green) for 60 min (Figure 1B). In this case, the IdU analog is incorporated in the absence of DNA damage, which is limited to the timing of the CldU pulse, as previously described (Berti et al., 2013; Edmunds et al., 2008; Jansen et al., 2014; Vallerga et al., 2015; Zellweger et al., 2015). Shortening of the IdU tracts on fibers with contiguous red and green tracts can be measured as a readout of nascent DNA degradation of stalled replication forks that have been subsequently remodeled and restarted in a very dynamic process (Lemaçon et al., 2017; Quinet et al., 2017a). Indeed, previous studies showed that forks can quickly restart after degradation suggesting that forks can undergo multiple rounds of degradation and restart during the 60-min window of CldU labeling (Lemaçon et al., 2017; Schlacher et al., 2011) (see also STAR Methods). Cisplatin treatment reduced the median IdU tract length in BRCA1-deficient cells by 30% compared to the untreated control, corresponding to >3 kb of DNA. Tract shortening was rescued by inhibiting the nuclease activity of MRE11 with mirin (Figure 1C), in agreement with previous studies showing that MRE11 promotes fork degradation in BRCA-deficient cells (Ray Chaudhuri et al., 2016; Schlacher et al., 2011, 2012; Ying et al., 2012). However, our results do not completely rule out the alternative possibility that tract shortening is due to an inhibitory effect of MRE11 on fork movement that is independent of fork degradation.

Next, we implemented a ''multiple-dose'' strategy where UW cells were first treated with 50 μM cisplatin for 1 h (''pre-dose,'' Figure 1D), a condition that did not lead to fork degradation as detected by DNA fiber assay (Figure S1A) or reduced cell viability within 24 h (Figure S1B). Twenty-four h after the pre-dose, we added a second cisplatin dose at a concentration that promotes high levels of nascent strand degradation when delivered as a single dose for 1 h (''challenging dose,'' 150 μM, Figure 1B). Of note, previous studies suggest that when patients receive the second round of cisplatin-based chemotherapy there is still approximately 13%–39% of platinum present in tumors from the first round of treatment (Holding et al., 1991), reflecting the

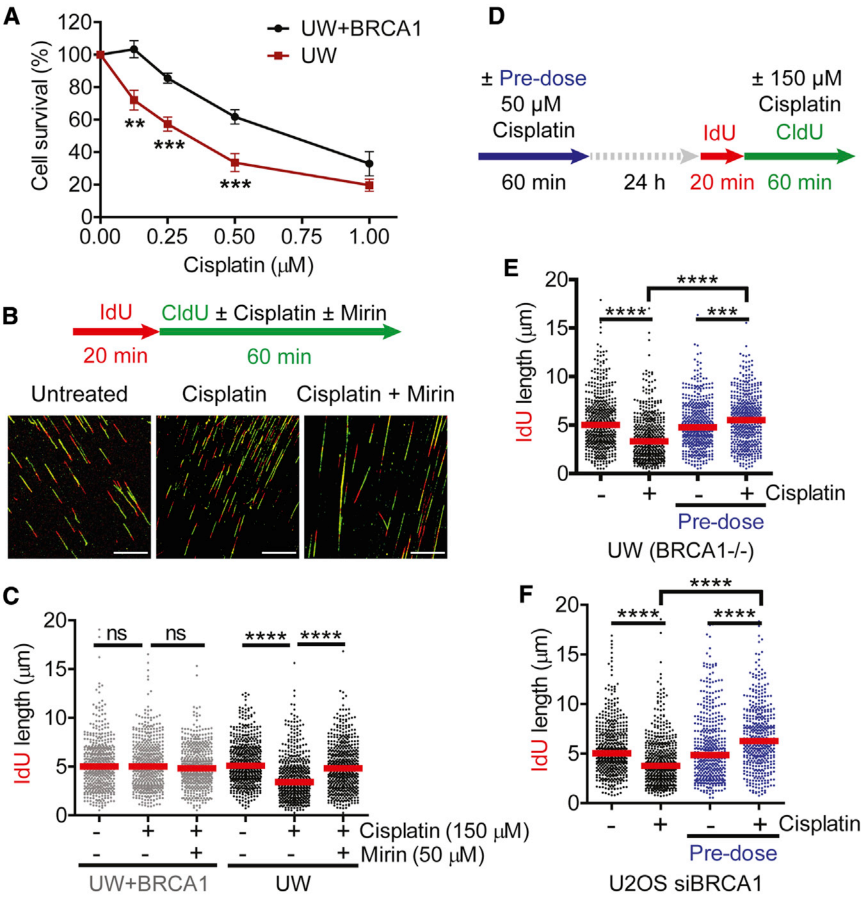

Figure 1. Treatment with a Cisplatin Pre-dose Abolishes Nascent DNA Degradation in BRCA1-Deficient Cells

(A) Cell survival of UW and UW+BRCA1 cells upon 6 days of chronic treatment with the indicated doses of cisplatin. Means ± SEM (n = 3). Statistics: two-way ANOVA followed by Bonferroni test. **$p < 0.01$, ***$p < 0.001$.

(B) Schematic of the DNA fiber assay and representative DNA fiber images of UW cells. IdU (red) was added for 20 min followed by CldU (green) for 60 min ± 150 μM cisplatin ± 50 μM mirin (added concomitantly with CldU labeling). Scale bar: 25 μm.

(C) Dot plot and median of IdU tract lengths in UW and UW+BRCA1 cells ± 150 μM cisplatin ± 50 μM mirin (n = 3). ns, non-significant, ****$p < 0.0001$.

(D) Schematic of the DNA fiber analysis with multiple cisplatin doses. Cells were treated with the cisplatin pre-dose (50 μM for 1 h). After 24 h, cells were treated with the second cisplatin dose (challenging dose, 150 μM) added concomitantly with CldU for 1 h.

(E and F) Dot plot and median of IdU tract lengths in UW (E) and U2OS cells depleted for BRCA1 (siBRCA1) (F) ± 150 μM cisplatin ± pre-dose (n = 3). ***$p < 0.001$, ****$p < 0.0001$.

See also Figure S1.

ratio between the two cisplatin doses used in our experimental conditions. Surprisingly, we found that the degradation phenotype observed with a single challenging dose of cisplatin is lost in the "multiple-dose" experiments (Figure 1E). The same was observed for CldU tracts (Figure S1C). Treatment with multiple cisplatin doses led to DNA fiber tracts that are even longer than those treated with the pre-dose alone, suggesting that multiple cisplatin doses promote an overall increase in the replication fork speed. These results were validated in U2OS cells siRNA depleted for BRCA1, confirming that this effect is not cell-type specific (Figures 1F, S1C, and S1D). To rule out the possibility that the rescue in fork degradation might be due to decreased levels of DNA damage following treatment with the cisplatin pre-dose, we monitored cisplatin-induced DNA adducts by immunofluorescence (Jazaeri et al., 2013; Tilby et al., 1991). Treatment with multiple cisplatin doses led to higher levels of cisplatin-induced DNA adducts compared to UW cells treated with the challenging dose alone (Figure S1E). Accordingly, multiple cisplatin doses led to increased levels of phosphorylated histone H2AX (γH2AX) compared to cells treated with the challenging dose alone (Figure S1F). To evaluate the effect of multiple cisplatin doses on replicating cells, we pulse-labeled S-phase UW cells with the thymidine analog EdU immediately before treating cells with the challenging dose of cisplatin and monitored their cell cycle progression after 24 h by flow cytometry (Figure S1G). We found that S-phase cells progressed faster through S-phase and reached G2/M earlier following treatment with multiple cisplatin doses compared to cells treated with a single challenging cisplatin dose. These results indicate that compensatory mechanisms rescue degraded replication forks upon multiple rounds of cisplatin treatment and drive faster progression through S-phase.

PRIMPOL Abolishes Nascent DNA Strand Degradation in BRCA1-Deficient Cells

We reasoned that canonical TLS polymerases or the PRIMPOL enzyme would be ideal candidates to rescue fork degradation upon multiple cisplatin doses because of their ability to overcome DNA lesions and reinitiate DNA synthesis. We investigated whether Pol η, REV1, and REV3L, the catalytic subunit of Pol ζ, were induced 24 h after treatment with the cisplatin pre-dose (50 μM) by RT-qPCR. We could not find any significant change in the expression levels of these canonical TLS polymerases (Figure S2A). However, we found that PRIMPOL mRNA and protein levels increased significantly in UW cells but not in UW+BRCA1 cells (Figures 2A–2C). This was accompanied by an increase in the levels of chromatin-bound PRIMPOL (Figure 2D). Similar results were obtained with BRCA1-depleted U2OS cells (Figure S2B). Importantly, PRIMPOL was also induced in response to ultraviolet irradiation (UVC) (Figure S2D), indicating that this phenotype is not specific to cisplatin.

Next, we repeated the DNA fiber assay with the multiple cisplatin doses in UW cells siRNA depleted for PRIMPOL. We ensured that PRIMPOL was depleted both during the pre-dose and the second dose of cisplatin (Figure 2E). PRIMPOL depletion

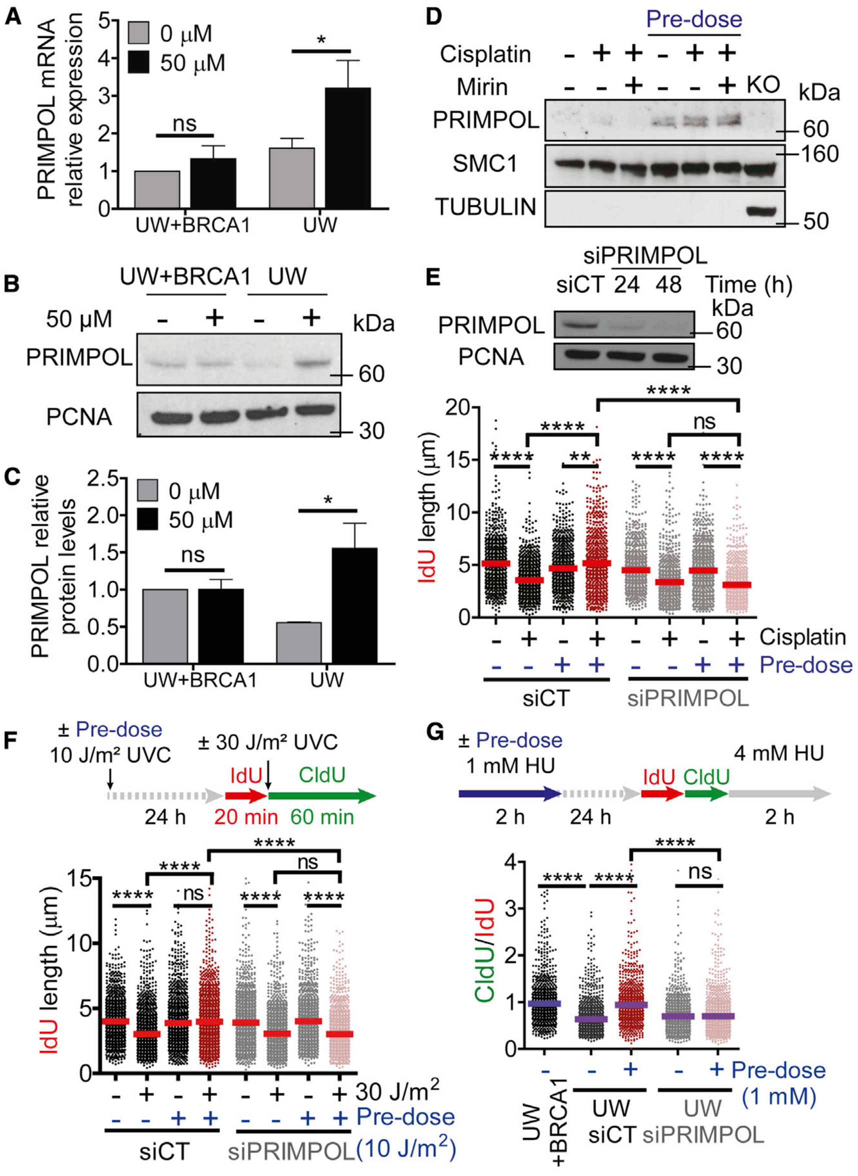

Figure 2. PRIMPOL Rescues Nascent Strand Degradation in BRCA1-Deficient Cells

(A–C) PRIMPOL mRNA (A) and protein (B and C) expression 24 h after treatment with 0 or 50 μM cisplatin in UW and UW+BRCA1 cells. (A) Means ± SEM (n = 5) are shown and presented relative to untreated UW+BRCA1 cells. Representative western blot (B) and quantifications (C) from three independent experiments. Statistics: two-way ANOVA followed by Bonferroni test. ns, non-significant, $*p < 0.05$.

(D) Chromatin-bound PRIMPOL in UW cells ± 150 μM cisplatin ± 50 μM mirin ± pre-dose. A representative western blot from three independent experiments is shown. Whole-cell extracts of PRIMPOL KO cells confirm the PRIMPOL antibody specificity.

(E) Expression of PRIMPOL after siRNA (siPRIMPOL) knockdown in UW cells 24 and 48 h after transfection (top). Dot plot and median of IdU tract lengths in siPRIMPOL or siRNA control (siCT) UW cells ± 150 μM cisplatin ± pre-dose (bottom) (n = 3). ns, non-significant, $**p < 0.01$, $****p < 0.0001$.

(F) Dot plot and median of IdU tract lengths in siCT or siPRIMPOL UW cells treated with ± 30 J/m^2 UVC ± pre-dose (10 J/m^2) (n = 3). ns, non-significant, $****p < 0.0001$.

(G) Dot plot and median of CldU/IdU ratios in siCT or siPRIMPOL UW cells treated with 4 mM HU for 2 h ± pre-dose (1 mM HU for 2 h) (n = 3). ns, non-significant, $****p < 0.0001$.

See also Figure S2.

restored fork degradation to the levels obtained using a single challenging cisplatin dose (Figure 2E). These results were validated by silencing PRIMPOL with a doxycycline inducible shRNA in BRCA1-depleted U2OS cells (Figure S2E) and using CRISPR/Cas9 PRIMPOL knockout U2OS cells depleted for BRCA1 (Figure S2F). Moreover, complementation of PRIMPOL knockout cells with exogenous PRIMPOL prevented fork degradation (Figure S2F). These results indicate that treatment with the pre-dose of cisplatin increases PRIMPOL levels and its recruitment to chromatin while abolishing nascent strand degradation in BRCA1-deficient cells. Importantly, pre-treating UW cells with a lower dose of UVC or hydroxyurea (HU) also led to PRIMPOL-dependent rescue of fork degradation upon treatment with a higher dose of the same genotoxic agent (Figures 2F and 2G), suggesting that the PRIMPOL-dependent adaptive response is activated by different types of replication challenges.

The PRIMPOL-Mediated Adaptive Response Is Dependent on ATR

The ATR pathway plays a central role in the control of replication fork stability (Saldivar et al., 2017) and has been implicated in the adaptive response to DNA damage (Christmann and Kaina, 2013). Treatment with the cisplatin pre-dose led to a significant increase in the levels of chromatin-bound RPA (Figures 3A and S3A) and a robust activation of the ATR pathway in UW cells, as indicated by increased levels of p-Chk1 and p-RPA (Figure 3B). This treatment also activated the ATR pathway in UW+BRCA1 cells, although to a lesser extent than in UW cells (Figures 3A and 3B). Next, we repeated the DNA fiber assay with multiple cisplatin doses in UW cells treated with the specific ATR inhibitor (ATRi) VE-821 (Figure 3C) (Reaper et al., 2011). VE-821 was added to the cell media during the cisplatin pre-dose and during the time that preceded the DNA fiber assay (Figure 3C) at a concentration (62.5 nM) that did not significantly affect cell viability under the experimental conditions used for the DNA fiber assay (Figure S3B) (Yazinski et al., 2017). Moreover, we removed the ATRi prior to the DNA fiber assay to uncouple the impact of ATR on the "pre-dose effect" from its role in replication dynamics. ATRi abolished the protective effect of the cisplatin pre-dose on replication fork stability, similarly to PRIMPOL depletion (Figures 3C and 2E). Moreover, RT-qPCR and western

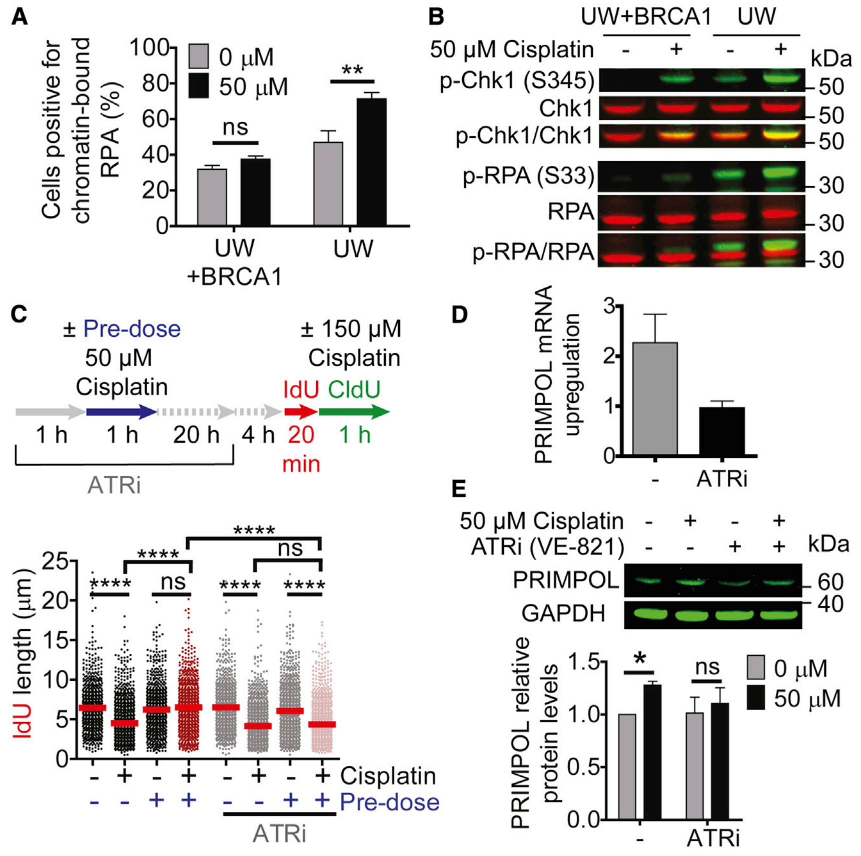

Figure 3. ATR Activity Controls PRIMPOL-Mediated Adaptive Response

(A) Percentage of UW+BRCA1 and UW cells positive for chromatin-bound RPA detected by flow cytometry 24 h after treatment with 0 or 50 μM cisplatin. Means ± SEM (n = 3). Statistics: two-way ANOVA followed by Bonferroni test. ns, non-significant, $^{**}p < 0.01$.

(B) p-Chk1 (S345, green), total Chk1 (red), p-RPA32 (S33, green), and total RPA (red) expression in UW+BRCA1 and UW cells 24 h upon 0 or 50 μM cisplatin. Simultaneous detection of phosphorylated form and total protein bands is shown in p-Chk1/Chk1 and p-RPA/RPA. A western blot representative of three independent experiments is shown.

(C) Schematic for the DNA fiber assay with the ATR inhibitor (ATRi) VE-821. 62.5 nM VE-821 was added 1 h prior to treatment with the pre-dose and removed from the media 4 h before performing the DNA fiber assay (top). Dot plot and median of IdU tract lengths in UW cells ± 150 μM cisplatin ± pre-dose ± ATRi (bottom) (n = 3). ns, non-significant, $^{****}p < 0.0001$.

(D and E) PRIMPOL mRNA (D) and protein (E) levels 24 h upon 0 or 50 μM cisplatin (pre-dose) ± ATRi. (D) Means ± SEM. Three independent biological replicates are shown and presented as fold change between 50 μM cisplatin and untreated samples. Representative western blot (E, top) and quantification (E, bottom) from four independent experiments. Statistics: two-way ANOVA followed by Bonferroni test. ns, non-significant, $^{*}p < 0.05$.

See also Figure S3.

blot analyses showed that PRIMPOL mRNA and protein levels were not induced by cisplatin treatment when ATR was inhibited, suggesting that ATR controls PRIMPOL induction at the transcriptional level (Figures 3D and 3E). Collectively, these data suggest that the ATR pathway is required for the PRIMPOL-dependent adaptive response to cisplatin.

PRIMPOL Primase Activity Suppresses DNA Degradation and Leads to ssDNA Gap Accumulation in BRCA1-Deficient Cells

Because PRIMPOL has both primase and polymerase activities (Bianchi et al., 2013; García-Gómez et al., 2013; Mourón et al., 2013), we overexpressed separation-of-function PRIMPOL mutants in UW cells and tested their impact on replication fork stability. A double mutation in the zinc-finger element present in its C-terminal domain (C419G/H426Y, CH variant) abolishes primase activity, preserving polymerase function (Mourón et al., 2013). Alanine substitutions of the two catalytic carboxylate residues Asp114 and Glu116 (AxA variant) disrupt both catalytic activities (García-Gómez et al., 2013; Mourón et al., 2013). Overexpression of wild-type PRIMPOL in BRCA1-deficient cells prevents fork degradation even after treatment with a single challenging cisplatin dose (Figures 4A and S2F). This result mimics the effect observed with pre-dose-dependent induction of PRIMPOL (Figure 1E) and further confirms that increased levels of PRIMPOL suppress DNA degradation. Conversely, overexpression of the catalytically dead PRIMPOL (AxA) or the primase-dead variant (CH) failed to prevent fork degradation (Figure 4A), suggesting that the primase activity of PRIMPOL is required to rescue fork degradation in BRCA1-deficient cells.

A PRIMPOL-dependent repriming mechanism would allow replication to skip the damaged DNA, leaving short ssDNA gaps behind the forks to be repaired after replication (Guilliam and Doherty, 2017; Rupp and Howard-Flanders, 1968; Yeeles et al., 2013). ssDNA gaps are typically shorter than 300–400 nucleotides and below the resolution of the DNA fiber technique. We therefore used a modified DNA fiber protocol where cells were treated with the ssDNA-specific S1 endonuclease after pulse labeling with the thymidine analogs. The shorter DNA fiber tracts generated by S1 cleavage were used as a readout for the presence of ssDNA gaps (Quinet et al., 2017a; Quinet et al., 2016) (Figure 4B). Treatment with the S1 nuclease led to significantly shorter DNA fiber tracts in the ‘‘multiple-dose’’ experiment compared to the pre-dose alone condition (Figure 4B). Interestingly, multiple doses of cisplatin also rescued nascent DNA degradation and led to ssDNA gaps in ongoing forks in U2OS cells siRNA depleted for BRCA2 (Figure S4), suggesting that PRIMPOL-mediated repriming is a more general mechanism of rescuing replication forks under conditions that lead to extensive reversed fork degradation.

To directly visualize the presence of internal ssDNA gaps behind forks, we analyzed the fine architecture of replication intermediates using a combination of *in vivo* psoralen crosslinking and EM (Figure 4C). This showed that treatment with multiple

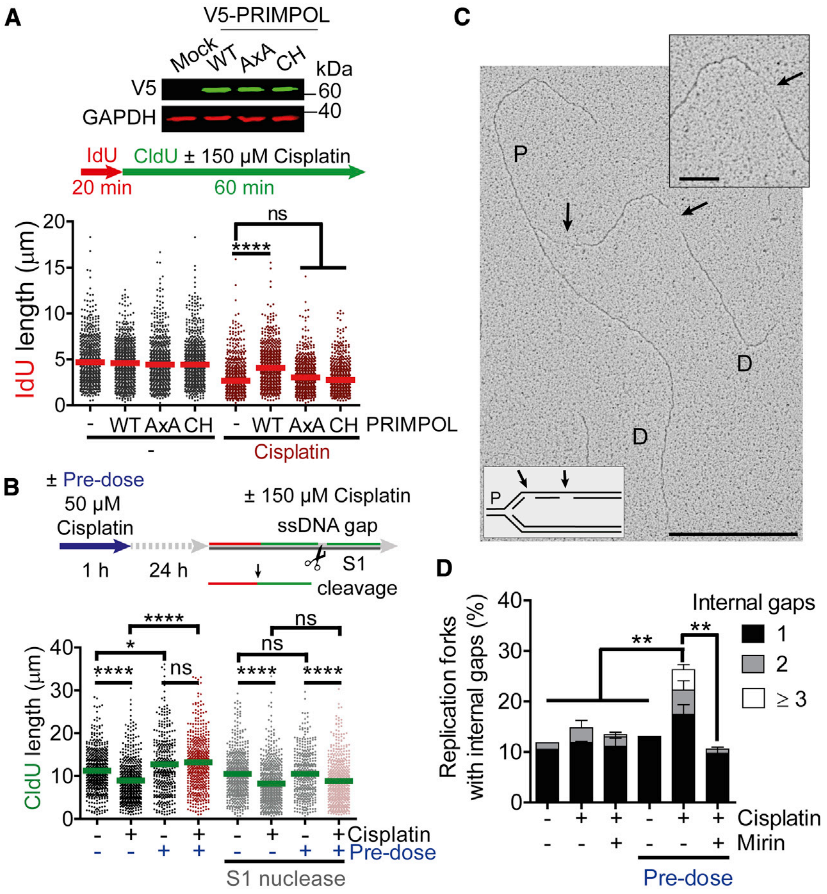

Figure 4. PRIMPOL Primase Activity Rescues Nascent Strand Degradation in BRCA1-Deficient Cells

(A) PRIMPOL overexpression in UW cells upon transfection with WT (wild-type), AxA (catalytic dead) and CH (primase dead only) V5-PRIMPOL constructs (top). Dot plot and median of IdU tract lengths in UW cells overexpressing the different constructs ± 150 μM cisplatin (bottom) (n = 3). ns, non-significant, ****p < 0.0001.

(B) Schematic for detection of ssDNA gaps using the ssDNA-specific S1 nuclease upon multiple treatments with cisplatin (top). Dot plot and median of CldU tract lengths in UW cells ± 150 μM cisplatin ± pre-dose ± S1 nuclease (bottom) (n = 3). ns, non-significant, *p < 0.05, ****p < 0.0001.

(C) Representative electron micrograph of a replication fork with internal ssDNA gaps behind the fork indicated by the arrows. Scale bar: 500 nm (left). Magnified internal ssDNA gap. Scale bar: 100 nm (right). P: parental strand, D: daughter strand.

(D) Percentage of replication forks with 1, 2 or ≥3 internal ssDNA gaps in UW cells ± 150 μM cisplatin ± pre-dose ± mirin. Means ± SEM (n = 3). Statistics: one-way ANOVA followed by Bonferroni test. **p < 0.01.
See also Figure S4 and Table S1.

cisplatin doses leads to an approximate 2-fold increase in the frequency of replication forks with internal ssDNA gaps compared to UW cells treated with a single-cisplatin dose (Figure 4D). Moreover, multiple doses of cisplatin led to a significant accumulation of intermediates with 2 or more internal ssDNA gaps (Figure 4D; Table S1A). Interestingly, inhibition of MRE11 nuclease activity by mirin decreased the frequency of replication forks with internal ssDNA gap from 26% to 10%, comparable to the levels of untreated cells. These results agree with previous studies showing that internal ssDNA gaps behind forks are suppressed by inhibition of MRE11 nuclease activity (Hashimoto et al., 2010). Together, these data suggest that increased levels of PRIMPOL promote repriming and accumulation of internal ssDNA gaps behind forks while suppressing nascent strand degradation.

PRIMPOL Overexpression Is Linked to Decreased Replication Fork Reversal

We reasoned that cells might "adapt" to conditions that promote extensive reversed fork degradation by suppressing replication fork reversal. To test this idea, we analyzed the frequency of reversed forks in UW cells that were either untreated, treated with a single cisplatin dose or treated with the cisplatin predose 24 h before treatment with the second dose. Treatment with a single challenging cisplatin dose (150 μM) led to a low frequency of reversed forks (approximately 11%) comparable to background levels (Figures 5A and 5B). Addition of mirin significantly increased reversed fork frequency (approximately 19%) and rescued the nascent DNA degradation observed by DNA fiber (Figure 1C), consistent with the model that MRE11 extensively degrades reversed forks in a BRCA-deficient background (Kolinjivadi et al., 2017; Lemaçon et al., 2017; Mijic et al., 2017; Taglialatela et al., 2017). In contrast, treatment with multiple cisplatin doses did not lead to fork degradation (Figure 1E). However, it still led to a low frequency of fork reversal events (approximately 10% of molecules analyzed) (Figure 5B), suggesting that this low frequency is not due to the degradation of reversed forks but rather to the suppression of fork reversal caused by the multiple-dose treatment. The interpretation of these results was, however, complicated by our finding that addition of mirin also restored reversed fork accumulation upon treatment with multiple cisplatin doses (Figure 5B). Based on the EM data showing that MRE11 inhibition suppresses the formation of ssDNA gaps in the multiple-cisplatin-dose experiments (Figure 4D), we speculate that addition of mirin either inhibits PRIMPOL-mediated repriming or prevents the MRE11-dependent enlargement of the ssDNA gaps after repriming, thus re-shifting the balance toward fork reversal.

To avoid any complication related to reversed fork degradation and the effect of mirin, we repeated the EM experiments in BRCA1-proficient U2OS cells (Figure 5C; Table S2). As expected, treatment with a single challenging dose of cisplatin led to a significant increase in the percentage of reversed forks. Treatment with multiple cisplatin doses led to a partial reduction in the frequency of reversed forks (from 22% to 15%) (Figure 5C). At the same time, it led to an increase in the percentage of

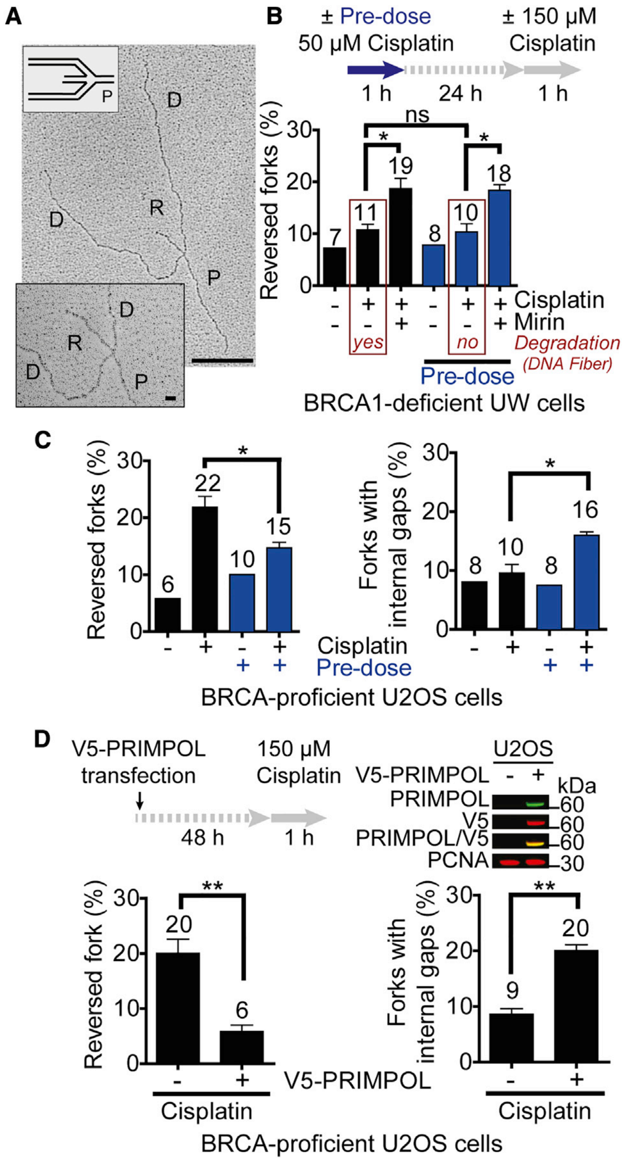

Figure 5. PRIMPOL Overexpression Is Linked to Decreased Fork Reversal

(A) Representative electron micrograph of a reversed replication fork. Scale bar: 200 nm. Magnified four-way junction at the reversed fork. Scale bar: 20 nm. P, parental strand; D, daughter strand; R, reversed arm.

(B) Schematic for the EM assay in UW cells treated with multiple doses of cisplatin (top). Percentage of reversed replication forks in UW cells ± 150 µM cisplatin ± pre-dose ± mirin. Means ± SEM (n = 3). Statistics: one-way ANOVA followed by Bonferroni test. (bottom). ns, non-significant, *$p < 0.05$.

(C) Percentage of reversed forks (left) and forks with internal gaps (right) in U2OS cells treated with 150 µM cisplatin ± pre-dose. Average ± SEM (n = 3). *$p < 0.05$.

(D) Schematic for the EM assay in U2OS cells overexpressing V5-PRIMPOL (top left). Expression of V5-PRIMPOL in U2OS cells (PRIMPOL in green, V5 in red, PRIMPOL/V5 shows simultaneous detection of both bands). PCNA was used a loading control (top right). Percentage of reversed forks (bottom left) and forks with internal gaps (bottom right) in U2OS mock-treated or overexpressing V5-PRIMPOL treated with 150 µM cisplatin. Means ± SEM (n = 3). Statistics: unpaired t test. **$p < 0.01$.

See also Tables S1, S2, and S3.

forks containing internal ssDNA gaps (Figure 5C). Even though these findings do not directly show that the ssDNA gaps are PRIMPOL-dependent, they strongly suggest that the PRIMPOL-mediated adaptive response is also activated in BRCA-proficient cells, although to a lesser extent than in BRCA1-deficient cells. Overall, the results support our model that treatment with multiple doses of cisplatin leads to decreased fork reversal while promoting repriming.

To directly test whether the upregulation of PRIMPOL is sufficient to promote repriming while restraining fork reversal, we overexpressed PRIMPOL by transfecting a V5-tagged PRIMPOL into BRCA-proficient U2OS cells and repeated the EM experiments upon treatment with a single challenging cisplatin dose (Figure 5D; Table S3). Overexpression of V5-PRIMPOL decreased the frequency of fork reversal events from 20% to approximately 6%. Moreover, it led to an increase of about 2-fold in the frequency of replication intermediates with internal ssDNA gaps (Figure 5D). Collectively, these experiments suggest that increasing PRIMPOL levels is sufficient to promote repriming while suppressing replication fork reversal.

Suppression of Replication Fork Reversal Promotes PRIMPOL-Dependent Repriming

Next, we asked whether suppression of fork reversal promotes fork repriming after a single challenging cisplatin dose (Figure 6A). To this end, we knocked down selected factors required for reversed fork formation, including the recombinase RAD51 (Zellweger et al., 2015), and the translocase SMARCAL1 (Bétous et al., 2012) in UW cells and in U2OS depleted for BRCA1. First, we confirmed that depletion of either factor restores fork protection and rescues tract shortening upon treatment with cisplatin, in agreement with previous studies showing that abolishing fork reversal suppresses DNA degradation in a BRCA1-deficient background (Figures 6B, 6C, S5A, and S5B) (Kolinjivadi et al., 2017; Lemaçon et al., 2017; Mijic et al., 2017; Taglialatela et al., 2017). However, tract shortening was not rescued in RAD51- or SMARCAL1-depleted cells treated with the S1 nuclease, unless PRIMPOL was co-depleted (Figures 6B, 6C, S5A, and S5B). This suggests that RAD51- and SMARCAL1-depleted cells accumulated ssDNA gaps, which were no longer detected upon PRIMPOL depletion. Of note, the levels of RAD51 and SMARCAL1 did not change upon treatment with the cisplatin pre-dose (Figure S2C), indicating that suppression of fork reversal upon multiple doses of cisplatin is not due to decreased levels of these factors.

Next, we sought to investigate whether suppressing fork reversal activates PRIMPOL-dependent repriming in the presence of functional BRCA. Depletion of SMARCAL1 also led to the accumulation of PRIMPOL-dependent ssDNA gaps in wild-type cells upon treatment with cisplatin (Figures 6D and S5C). Our previous studies showed that PARP activity promotes the accumulation of reversed forks by inhibiting RECQ1 fork-restoration activity, thus preventing premature reversed fork restart (Berti et al., 2013). We found that preventing the accumulation of reversed forks by using the PARP inhibitor olaparib also led to PRIMPOL-dependent ssDNA gaps (Figures 6E and S5D). In summary, these results indicate that suppression of fork reversal

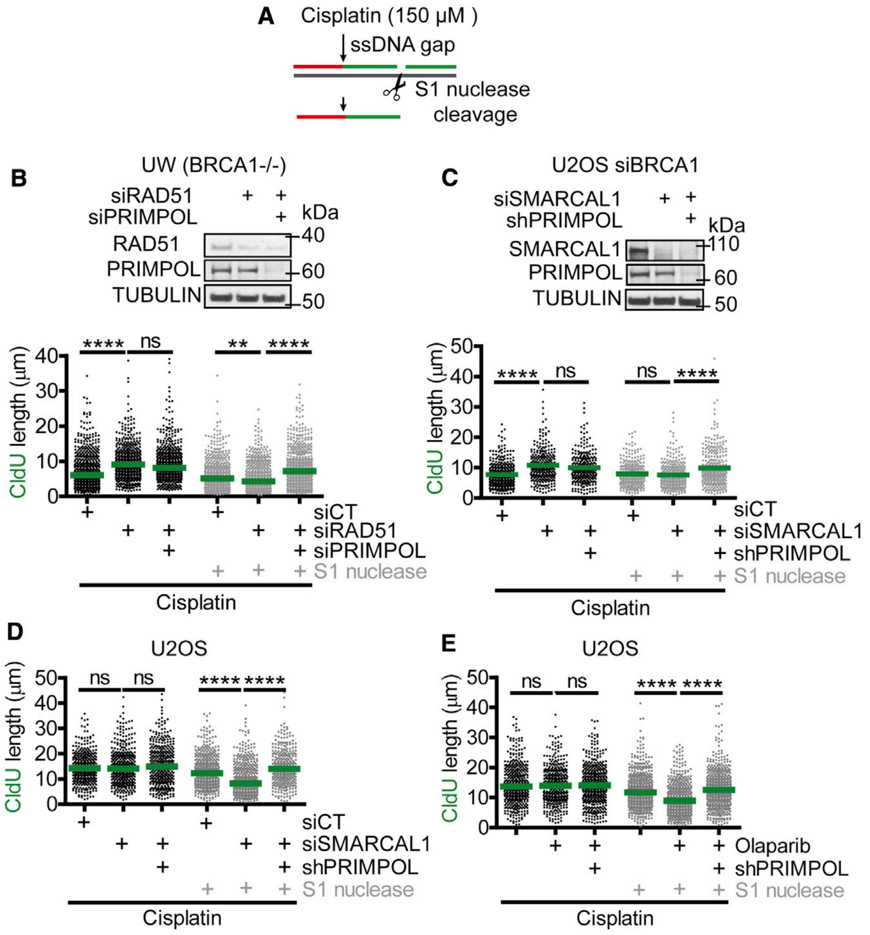

Figure 6. Depletion of Fork Reversal Factors Leads to Accumulation of PRIMPOL-Dependent ssDNA Gaps

(A) Schematic for detection of ssDNA gaps using the ssDNA-specific S1 nuclease upon treatment with cisplatin (150 μM).

(B) Expression of RAD51 and PRIMPOL after siRNA (siRAD51 and siPRIMPOL) knockdown in UW cells 48 h after transfection (top). Dot plot and median of CldU tract lengths upon treatment with 150 μM cisplatin in UW cells depleted for RAD51, PRIMPOL, or RAD51/PRIMPOL ± S1 nuclease (bottom) (n = 3). ns, non-significant, $**p < 0.01$, $****p < 0.0001$.

(C) Expression of SMARCAL1 48 h after depletion with siRNA (siSMARCAL1) and PRIMPOL upon addition of doxycycline (DOX) in U2OS siBRCA1 cells stably expressing a DOX-inducible shPRIMPOL (shPRIMPOL) (top). Dot plot and median of CldU tract lengths upon treatment with 150 μM cisplatin in U2OS siBRCA1 cells depleted for SMARCAL1, PRIMPOL, or SMARCAL1/PRIMPOL ± S1 nuclease (bottom) (n = 2). ns, non-significant, $****p < 0.0001$.

(D) Dot plot and median of CldU tract lengths upon treatment with 150 μM cisplatin in U2OS cells depleted for SMARCAL1, PRIMPOL, or SMARCAL1/PRIMPOL ± S1 nuclease (bottom) (n = 3). ns, non-significant, $****p < 0.0001$.

(E) Dot plot and median of CldU tract lengths upon treatment with 150 μM cisplatin in U2OS cells depleted for PRIMPOL (shPRIMPOL) ± PARPi and ± S1 nuclease (n = 3). ns, non-significant, $****p < 0.0001$.

See also Figure S5.

shifts the balance to PRIMPOL-dependent repriming events both in BRCA-deficient and -proficient cells.

Modulating PRIMPOL Levels Impacts Cell Survival

ATR inhibition has been shown to synergize with cisplatin treatment (Huntoon et al., 2013; Karnitz and Zou, 2015). Our model that ATR activity is critical for PRIMPOL-dependent fork protection (Figure 3C) suggests that the PRIMPOL-mediated adaptive response might also modulate cisplatin toxicity in BRCA1-deficient cells. We found that the synergistic effect of ATR inhibition and cisplatin in UW cells was no longer observed upon overexpression of PRIMPOL (Figures 7A and S7A). This data indicate that PRIMPOL overexpression in BRCA1-deficient cells decreases sensitivity to combined treatment with ATRi and cisplatin, which is currently studied in clinical trials (Karnitz and Zou, 2015). We also observed that depletion of PRIMPOL affected cell proliferation and cell viability in BRCA1-deficient cells in the absence of genotoxic treatment, supporting the notion that PRIMPOL becomes essential for cell survival in BRCA1 null cells (Figures 7B and S7B).

Based on our data that fork reversal and repriming are two alternative pathways to cope with cisplatin-induced lesions, we investigated whether suppressing both pathways impacts cancer cell sensitivity to cisplatin. To suppress fork reversal, we used olaparib at a concentration that did not significantly impair viability of wild-type and PRIMPOL-depleted U2OS cells (Figure S6A). Olaparib significantly increased the cisplatin sensitivity of PRIMPOL-depleted cells (Figure S6A). As an alternative strategy to further investigate the coordination between repriming and fork reversal, we used previously established knockout DT40 cell lines (Keka et al., 2015; Schiavone et al., 2016). We were readily able to generate a *primpol* −/− *smarcal1* +/− line by conventional gene targeting with high efficiency (7/48) (Figures S6B and S6C). However, we failed to generate *primpol* −/− *smarcal1*−/− double knockouts by sequentially targeting the second *smarcal1* allele, even after analyzing 495 independently derived clones, suggesting that DT40 cells carrying *primpol* and *smarcal1* mutations are inviable. While the *smarcal1* +/− did not show any growth defects when compared to wild-type cells, a heterozygous deletion of *smarcal1* in a *primpol*-deficient background (*primpol* −/− *smarcal1* +/−) showed a major increase in the doubling time (Figures 7C and S6D). Importantly, we noted that the doubling time of *primpol* −/− *smarcal1* +/− mutants reduced over time spent in culture (Figures 7C and S6D), which is indicative of the adaptive response to the paucity of SMARCAL1 protein. This idea is supported by the observation that *primpol* −/− DT40 cells upregulated *SMARCAL1* expression (Figure S6C). Together, these data indicate that the combined suppression of fork reversal

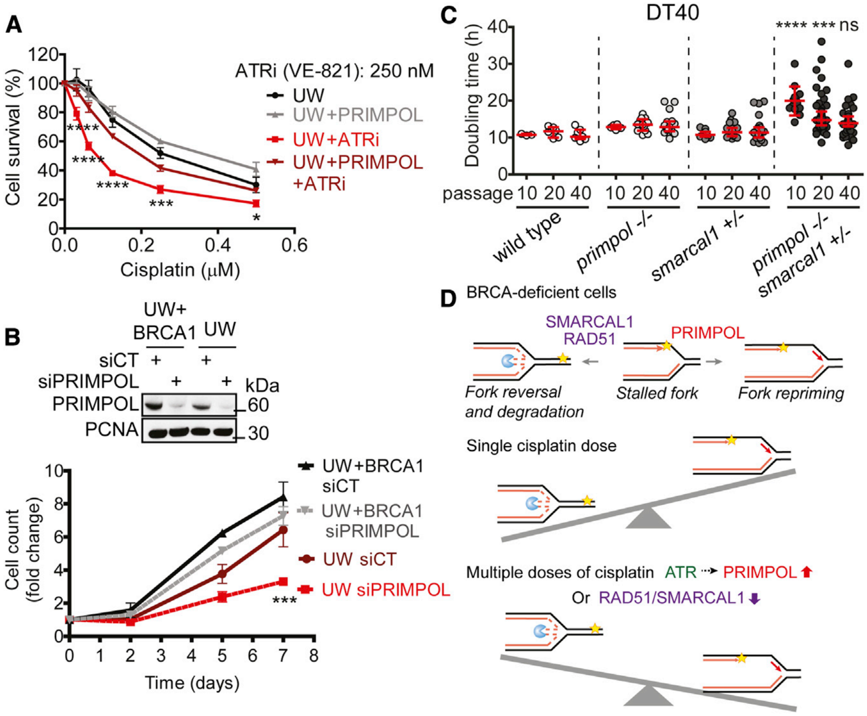

Figure 7. Impact of PRIMPOL on Cell Survival

(A) Cell survival of UW and UW+PRIMPOL cells upon 6 days of chronic treatment with ATR inhibitor (ATRi, VE-821, 250 nM) and the indicated doses of cisplatin. Means ± SEM (n = 3). Statistics: two-way ANOVA followed by Bonferroni test comparing UW+ATRi versus UW+PRIMPOL+ATRi. *p < 0.05, ***p < 0.001, ****p < 0.0001.

(B) Expression of PRIMPOL after siRNA (siPRIMPOL) in UW+BRCA1 and UW cells (top). Cell count in PRIMPOL-depleted UW+BRCA1 and UW cells. Means ± SEM (n = 3) (bottom). Statistics: two-way ANOVA followed by Bonferroni test comparing UW siCT versus UW siPRIMPOL. ***p < 0.001.

(C) Doubling time of DT40 cell mutants over the course of approximately 40 passages post-transfection with the *smarcal1* targeting construct. Each circle represents an individual measurement of doubling time derived from three independent experiments, repeated in duplicate and on multiple independently derived clones (two *primpol* −/−; three *smarcal1* +/− ; and five *primpol* −/− *smarcal1* +/−). Means and SD plotted as line and whiskers. Statistics: Kruskal-Wallis test for difference between *smarcal1* +/− and *primpol* −/− *smarcal1* +/−. ns, non-significant, ***p < 0.001, ****p < 0.0001.

(D) Proposed model. Fork repriming and reversal are two alternative mechanisms by which cells deal with cisplatin-induced DNA lesions (top). Upon treatment with a single cisplatin dose, fork reversal is the most frequent event. However, reversed forks are targeted by nucleases in a BRCA-deficient background leading to nascent DNA degradation (middle). Multiple treatments with cisplatin lead to an ATR-dependent upregulation of PRIMPOL, shifting the balance toward repriming events while suppressing fork reversal. Alternatively, depletion of fork reversal factors, such as RAD51 and SMARCAL1, also favors PRIMPOL-dependent repriming in both BRCA-deficient and -proficient cells (bottom).

See also Figures S6 and S7.

and repriming mechanisms impair cell survival and the ability of cells to cope with cisplatin-induced lesions.

DISCUSSION

PRIMPOL Is Involved in the Adaptive Response to Genotoxic Stress

Understanding how cells adapt to multiple drug doses has become increasingly important in the context of cancer treatment where patients are treated with multiple rounds of chemotherapeutics. However, little is known about this process, and mechanistic insights into how multiple dose regimens affect the DNA replication response are lacking. Our data show that a PRIMPOL-dependent pathway becomes activated at later time points after cisplatin exposure, among other challenges to replication, including HU and UV, uncovering a previously unappreciated role for PRIMPOL in replication fork protection and genomic stability.

How cells choose between PRIMPOL repriming and other replication stress response mechanisms, such as TLS or replication fork reversal, remains unclear. We found that cells adapt to cisplatin treatment by promoting PRIMPOL repriming while suppressing replication fork reversal. This adaptive response is regulated by ATR and is more marked in genetic backgrounds where fork reversal leads to pathological DNA degradation (e.g., BRCA-deficient cells). Increased PRIMPOL repriming likely reduces fork stalling, thereby diminishing the requirement for fork reversal. In line with this model, loss of PRIMPOL in BRCA1-deficient cells treated with multiple doses of cisplatin restores fork degradation, suggesting that forks are again prone to reverse in the absence of PRIMPOL. This notion was supported by experiments showing that PRIMPOL loss in BRCA1-deficient cells does not restore fork degradation when fork reversal is impaired in the absence of SMARCAL1 or RAD51. Based on these findings, we propose that fork repriming and fork reversal are two alternative mechanisms by which cells deal with cisplatin-induced DNA lesions. The balance between these two pathways can be tilted toward fork repriming either by increasing PRIMPOL expression levels, as observed upon treatment with multiple cisplatin doses, or by depleting fork reversal factors (Figure 7D).

ATR Regulates the PRIMPOL-Mediated Adaptive Response to Genotoxic Stress

ATR is a key factor for the regulation of the replication stress response in S-phase (Saldivar et al., 2017). Moreover, recent studies have pointed to a role for ATR/Chk1 and ATM/Chk2 pathways in the adaptive response to genotoxic stress (Bertoli et al., 2013; Buisson et al., 2015; Cambindo Botto et al., 2018; Christmann and Kaina, 2013; Gomes et al., 2019; Kim et al., 2018). Our data show that pre-treatment of BRCA1-deficient cells with cisplatin leads to increased levels

of chromatin-bound RPA as well as p-Chk1 and p-RPA. These data support a model where increased ssDNA formation due to fork degradation in BRCA1-deficient cells leads to high levels of ssDNA-bound RPA and consequent activation of the ATR pathway. In turn, activation of the ATR pathway is essential for PRIMPOL upregulation and for the rescue of fork degradation in BRCA1-deficient cells. Altogether, these data suggest that *PRIMPOL* is a new gene involved in ATR-mediated adaptive response to genotoxic stress. In line with these findings, we found that PRIMPOL overexpression renders BRCA1-deficient cells less sensitive to combined treatment with cisplatin and ATRi, providing a new rationale for the mechanism underlying the synergistic effect between cisplatin and ATR inhibition (Huntoon et al., 2013). Our data suggest that treatment with the cisplatin pre-dose also leads to activation of the PRIMPOL pathway in BRCA-proficient cells, although to a lesser extent than in BRCA1-deficient cells. These data are consistent with our finding that treatment with the cisplatin pre-dose also activates the ATR pathway in BRCA-proficient cells, although its activation is less marked than in BRCA1-deficient cells. These findings point to a model where the extent of activation of the ATR pathway dictates the level to which the PRIMPOL-pathway is activated.

Notably, a recent study reported that fork slowing and reversal are not restricted to sites of inter/intrastrand crosslinks (ICLs) and are instead genome-wide responses to DNA damage dependent on ATR activity (Mutreja et al., 2018). Future studies will be necessary to properly address whether the cisplatin-induced effects described here are also due to genome-wide signaling. In addition, the studies of Mutreja et al. suggest that ATR activity is required to promote fork reversal, whereas we found that the same activity is required for PRIMPOL-mediated adaptive response. Importantly, these observations are not contradictory, as Mutreja et al. studied the effect of ATR on replication fork dynamics by inhibiting ATR during the DNA fiber labeling, whereas we studied the effect of ATR activity on the adaptive response to cisplatin by removing the ATRi prior to performing the DNA fiber assay.

Finally, we found that the formation of ssDNA gaps behind forks is also dependent on the exonuclease activity of MRE11 (Figure 4D), in agreement with previous studies showing that inhibition of MRE11 activity suppresses ssDNA gaps behind forks in *Xenopus laevis* extracts depleted for RAD51 (Hashimoto et al., 2010). However, our chromatin fractionation experiments showed that inhibition of MRE11 activity does not affect PRIMPOL loading to DNA, indicating that MRE11 might act downstream of PRIMPOL binding to DNA (Figure 2D). We propose that ssDNA gaps might be enlarged by the action of the MRE11 nuclease in order to promote DNA damage bypass and suppress fork reversal in BRCA1-deficient cells treated with multiple cisplatin doses. This suggests that controlled degradation promoted by MRE11 is a physiological and frequent event that plays an important role during replication stress response (Costanzo, 2011). Interestingly, MRE11 nuclease activity was also shown to play a role in activating the ATR kinase in mice (Buis et al., 2008), pointing to a link between the MRE11 nuclease processing, ATR activation, and the PRIMPOL-dependent pathway.

PRIMPOL Primase Activity Is Required for Replication Fork Protection

The PRIMPOL enzyme possesses both primase and TLS activity *in vitro* (Bianchi et al., 2013; García-Gómez et al., 2013; Mourón et al., 2013; Wan et al., 2013). We have shown that the primase activity of PRIMPOL is essential to rescue nascent DNA strand degradation in BRCA1-deficient cells treated with cisplatin, consistent with the notion that PRIMPOL mostly acts as a repriming enzyme (Keen et al., 2014b; Kobayashi et al., 2016; Mourón et al., 2013; Schiavone et al., 2016). While the TLS activity of PRIMPOL is capable of bypassing some specific DNA lesions, such as 8oxoG (Bianchi et al., 2013; García-Gómez et al., 2013; Keen et al., 2014b; Mourón et al., 2013), the active site of PRIMPOL cannot accommodate bulky DNA adducts, arguing against a general TLS role (Rechkoblit et al., 2016). Accordingly, our model considers the more likely scenario that PRIMPOL-mediated repriming occurs downstream of an intrastrand crosslink in the leading strand template (Figure 7D). Although cisplatin mainly generates intra-strand adducts (Poklar et al., 1996), ~5% of lesions are inter-strand crosslinks (ICLs) (Deans and West, 2011). ICLs can be "traversed" in a reaction mediated by the FANCM/MHF DNA translocase, but how replication resumes after traverse remains unclear (Huang et al., 2013). A tantalizing hypothesis deserving investigation is that PRIMPOL facilitates fork progression through ICL lesions, leaving the ICL in ssDNA gaps behind forks to be repaired post-replicatively (Figure S7C).

Cells Cope with Cisplatin-Induced DNA Lesions by Balancing Fork Reversal and Repriming

We propose that fork repriming and reversal are two alternative and tightly controlled mechanisms by which cells can deal with cisplatin-induced DNA lesions. We have shown that the balance between these two pathways can be altered by increasing levels of PRIMPOL, leading to a decrease in fork reversal and an increase in ssDNA gap accumulation. Moreover, depletion of fork reversal factors, such as RAD51 and SMARCAL1, or inhibition of PARP activity, which prevents accumulation of reversed forks (Berti et al., 2013), leads to the accumulation of PRIMPOL-dependent ssDNA gaps in both BRCA-deficient and -proficient cells. These findings support a model where the equilibrium between fork reversal and repriming can be tilted in favor of one pathway by simply down or upregulating factors required for the other pathway (Figure 7D).

In agreement with our model, UVC-treated cells depleted of PRIMPOL show an increase in RAD51 loading to the chromatin (Bianchi et al., 2013), whereas cells depleted for RAD51 and exposed to UVC show excessive fork elongation dependent on PRIMPOL (Vallerga et al., 2015). The balance between fork repriming and reversal is likely relevant in other eukaryotes. For example, Pol α/primase *Saccharomyces cerevisiae* mutants that are unable to promote fork repriming accumulate aberrant reversed forks upon methyl methanesulfonate (MMS) treatment (Fumasoni et al., 2015). In addition, *Xenopus laevis* extracts depleted for RAD51 accumulate ssDNA gaps behind forks upon MMS and UVC exposure (Hashimoto et al., 2010), similar to our findings.

The notion that fork reversal and repriming are two alternative pathways that cells use to cope with cisplatin-induced lesions is

further supported by our findings that suppressing both pathways by inhibiting PARP activity and depleting PRIMPOL significantly sensitizes U2OS cells to cisplatin treatment. Moreover, knocking out *primpol* leads to a defect in cell growth in unperturbed DT40 cells. Further exacerbation in cell growth upon ablation of one of the *smarcal1* alleles in *primpol* mutant cells, together with failure to generate a double *primpol/smarcal1* knockout implies a major genetic interaction, emphasizing that cells need at least one of the two pathways to be functional in order to survive. Of note, loss of PRIMPOL in BRCA1-deficient cancer cells significantly affects cell growth and viability, even in the absence of exogenous damage, underscoring the relevance of the PRIMPOL pathway for BRCA1-deficient cell survival.

In conclusion, our work introduces the important concept that multiple-dose regimens are essential for a full understanding of how replication copes with chemotherapeutic insults. We envision that the use of this multiple-dose approach will lead to a revision of current models for how replication copes with DNA lesions in tumors. We provided clear evidence that cancer cells lacking BRCA proteins adapt to genotoxic stress by promoting PRIMPOL-dependent replication fork repriming at the expense of replication fork reversal, thereby avoiding toxic nascent strand degradation. In future work, it will be important to further elucidate the underlying molecular mechanisms that fine-tune the balance between replication fork repriming and fork reversal and to understand how combinatorial treatments that block both processes can be exploited therapeutically to increase cancer cell chemosensitivity.

STAR★METHODS

Detailed methods are provided in the online version of this paper and include the following:

- KEY RESOURCES TABLE
- LEAD CONTACT AND MATERIAL AVAILABILITY
- EXPERIMENTAL MODELS AND SUBJECT DETAILS
  - Cell culture and cell lines
  - Generation of a stable cell line overexpressing PRIMPOL
  - Generation of PRIMPOL KO cells
- METHOD DETAILS
  - Gene silencing with RNAi
  - Transfections of PRIMPOL constructs
  - Drugs and cell treatments
  - Cell survival
  - DNA fiber assay
  - RT-qPCR
  - Chromatin isolation and Western Blot
  - Electron microscopy
  - Doubling time experiments in DT40 cells
  - Cell cycle analysis
  - Immunofluorescence
  - Chromatin-bound RPA32 detection by flow cytometry
- QUANTIFICATION AND STATISTICAL ANALYSIS
- DATA AND CODE AVAILABILITY

SUPPLEMENTAL INFORMATION

Supplemental Information can be found online at https://doi.org/10.1016/j.molcel.2019.10.008.

ACKNOWLEDGMENTS

We would like to thank Shunichi Takeda (Kyoto University) for providing DT40 *smarcal1* mutant and targeting constructs and Aidan Doherty (University of Sussex) for providing the *primpol* DT40 targeting constructs. We thank the Research Microscopy Core Facility and the Flow Cytometry Research Core Facility of Saint Louis University for technical support. We thank the Genome Engineering and iPSC Center (GEiC) at Washington University in St. Louis for gRNA validation services and generation of the PRIMPOL KO U2OS cells. The work in the A.V. laboratory is supported by NIH grants R01CA237263 and R01GM108648, by DOD BRCP Breakthrough Award BC151728, and by the Siteman Cancer Center at Washington University in St. Louis. Work in the J.E.S. laboratory is supported by the UK Medical Research Council through a central grant to the Laboratory of Molecular Biology (U105178808). The work in J.M. laboratory is supported by the Spanish Ministry of Science, Innovation, and Universities (grant BFU2016-80402-R, co-financed with European Union ERDF funds). Work in the L.B laboratory is supported by NIH grant R01HL137793 and the Siteman Cancer Center at Washington University in St. Louis.

AUTHOR CONTRIBUTIONS

A.Q. designed the project and performed most of the experiments with the assistance of S.T., D.C.M., E.C., and M.W.; J.J. performed the EM experiments with the assistance of A.Q. and D.L.; S.S. performed the experiments with the DT40 cells; D.G.-A. performed the chromatin fractionation experiments; S.T. contributed to the analysis of the colony-forming assays; A.T.V. established the stable UW+PRIMPOL cell line. L.B., J.M., and J.E.S. assisted with experimental design and manuscript finalization; A.V. designed and supervised the project and wrote the manuscript with the assistance of A.Q.

DECLARATION OF INTERESTS

The authors decleare no competing interests.

Received: January 9, 2019
Revised: July 29, 2019
Accepted: October 7, 2019
Published: October 29, 2019

REFERENCES

Antoniou, A., Pharoah, P.D., Narod, S., Risch, H.A., Eyfjord, J.E., Hopper, J.L., Loman, N., Olsson, H., Johannsson, O., Borg, A., et al. (2003). Average risks of breast and ovarian cancer associated with BRCA1 or BRCA2 mutations detected in case Series unselected for family history: a combined analysis of 22 studies. Am. J. Hum. Genet. *72*, 1117–1130.

Berti, M., Ray Chaudhuri, A., Thangavel, S., Gomathinayagam, S., Kenig, S., Vujanovic, M., Odreman, F., Glatter, T., Graziano, S., Mendoza-Maldonado, R., et al. (2013). Human RECQ1 promotes restart of replication forks reversed by DNA topoisomerase I inhibition. Nat. Struct. Mol. Biol. *20*, 347–354.

Bertoli, C., Skotheim, J.M., and de Bruin, R.A. (2013). Control of cell cycle transcription during G1 and S phases. Nat. Rev. Mol. Cell Biol. *14*, 518–528.

Bétous, R., Mason, A.C., Rambo, R.P., Bansbach, C.E., Badu-Nkansah, A., Sirbu, B.M., Eichman, B.F., and Cortez, D. (2012). SMARCAL1 catalyzes fork regression and Holliday junction migration to maintain genome stability during DNA replication. Genes Dev. *26*, 151–162.

Bianchi, J., Rudd, S.G., Jozwiakowski, S.K., Bailey, L.J., Soura, V., Taylor, E., Stevanovic, I., Green, A.J., Stracker, T.H., Lindsay, H.D., and Doherty, A.J. (2013). PrimPol bypasses UV photoproducts during eukaryotic chromosomal DNA replication. Mol. Cell *52*, 566–573.

Buis, J., Wu, Y., Deng, Y., Leddon, J., Westfield, G., Eckersdorff, M., Sekiguchi, J.M., Chang, S., and Ferguson, D.O. (2008). Mre11 nuclease activity has essential roles in DNA repair and genomic stability distinct from ATM activation. Cell *135*, 85–96.

Buisson, R., Boisvert, J.L., Benes, C.H., and Zou, L. (2015). Distinct but Concerted Roles of ATR, DNA-PK, and Chk1 in Countering Replication Stress during S Phase. Mol. Cell *59*, 1011–1024.

Cambindo Botto, A.E., Muñoz, J.C., and Muñoz, M.J. (2018). Coupling between nucleotide excision repair and gene expression. RNA Biol. *15*, 845–848.

Carvajal-Maldonado, D., Byrum, A.K., Jackson, J., Wessel, S., Lemaçon, D., Guitton-Sert, L., Quinet, A., Tirman, S., Graziano, S., Masson, J.Y., et al. (2019). Perturbing cohesin dynamics drives MRE11 nuclease-dependent replication fork slowing. Nucleic Acids Res. *47*, 1294–1310.

Chatterjee, N., and Walker, G.C. (2017). Mechanisms of DNA damage, repair, and mutagenesis. Environ. Mol. Mutagen. *58*, 235–263.

Christmann, M., and Kaina, B. (2013). Transcriptional regulation of human DNA repair genes following genotoxic stress: trigger mechanisms, inducible responses and genotoxic adaptation. Nucleic Acids Res. *41*, 8403–8420.

Christmann, M., Boisseau, C., Kitzinger, R., Berac, C., Allmann, S., Sommer, T., Aasland, D., Kaina, B., and Tomicic, M.T. (2016). Adaptive upregulation of DNA repair genes following benzo(a)pyrene diol epoxide protects against cell death at the expense of mutations. Nucleic Acids Res. *44*, 10727–10743.

Costanzo, V. (2011). Brca2, Rad51 and Mre11: performing balancing acts on replication forks. DNA Repair (Amst.) *10*, 1060–1065.

Daza-Martin, M., Starowicz, K., Jamshad, M., Tye, S., Ronson, G.E., MacKay, H.L., Chauhan, A.S., Walker, A.K., Stone, H.R., Beesley, J.F.J., et al. (2019). Isomerization of BRCA1-BARD1 promotes replication fork protection. Nature *571*, 521–527.

Deans, A.J., and West, S.C. (2011). DNA interstrand crosslink repair and cancer. Nat. Rev. Cancer *11*, 467–480.

DelloRusso, C., Welcsh, P.L., Wang, W., Garcia, R.L., King, M.C., and Swisher, E.M. (2007). Functional characterization of a novel BRCA1-null ovarian cancer cell line in response to ionizing radiation. Mol. Cancer Res. *5*, 35–45.

Edmunds, C.E., Simpson, L.J., and Sale, J.E. (2008). PCNA ubiquitination and REV1 define temporally distinct mechanisms for controlling translesion synthesis in the avian cell line DT40. Mol. Cell *30*, 519–529.

Feng, W., and Jasin, M. (2017). BRCA2 suppresses replication stress-induced mitotic and G1 abnormalities through homologous recombination. Nat. Commun. *8*, 525.

Ford, D., Easton, D.F., Stratton, M., Narod, S., Goldgar, D., Devilee, P., Bishop, D.T., Weber, B., Lenoir, G., Chang-Claude, J., et al.; The Breast Cancer Linkage Consortium (1998). Genetic heterogeneity and penetrance analysis of the BRCA1 and BRCA2 genes in breast cancer families. Am. J. Hum. Genet. *62*, 676–689.

Forment, J.V., and Jackson, S.P. (2015). A flow cytometry-based method to simplify the analysis and quantification of protein association to chromatin in mammalian cells. Nat. Protoc. *10*, 1297–1307.

Fumasoni, M., Zwicky, K., Vanoli, F., Lopes, M., and Branzei, D. (2015). Error-free DNA damage tolerance and sister chromatid proximity during DNA replication rely on the Polα/Primase/Ctf4 Complex. Mol. Cell *57*, 812–823.

García-Gómez, S., Reyes, A., Martínez-Jiménez, M.I., Chocrón, E.S., Mourón, S., Terrados, G., Powell, C., Salido, E., Méndez, J., Holt, I.J., and Blanco, L. (2013). PrimPol, an archaic primase/polymerase operating in human cells. Mol. Cell *52*, 541–553.

Gomes, L.R., Rocha, C.R.R., Martins, D.J., Fiore, A.P.Z.P., Kinker, G.S., Bruni-Cardoso, A., and Menck, C.F.M. (2019). ATR mediates cisplatin resistance in 3D-cultured breast cancer cells via translesion DNA synthesis modulation. Cell Death Dis. *10*, 459.

Guilliam, T.A., and Doherty, A.J. (2017). PrimPol-Prime Time to Reprime. Genes (Basel) *8*, E20.

Hashimoto, Y., Ray Chaudhuri, A., Lopes, M., and Costanzo, V. (2010). Rad51 protects nascent DNA from Mre11-dependent degradation and promotes continuous DNA synthesis. Nat. Struct. Mol. Biol. *17*, 1305–1311.

Heller, R.C., and Marians, K.J. (2006). Replication fork reactivation downstream of a blocked nascent leading strand. Nature *439*, 557–562.

Helm, C.W., and States, J.C. (2009). Enhancing the efficacy of cisplatin in ovarian cancer treatment - could arsenic have a role. J. Ovarian Res. *2*, 2.

Higgins, N.P., Kato, K., and Strauss, B. (1976). A model for replication repair in mammalian cells. J. Mol. Biol. *101*, 417–425.

Holding, J.D., Lindup, W.E., Bowdler, D.A., Siodlak, M.Z., and Stell, M. (1991). Disposition and tumour concentrations of platinum in hypoalbuminaemic patients after treatment with cisplatin for cancer of the head and neck. Br. J. Clin. Pharmacol *32*, 173–179.

Huang, J., Liu, S., Bellani, M.A., Thazhathveetil, A.K., Ling, C., de Winter, J.P., Wang, Y., Wang, W., and Seidman, M.M. (2013). The DNA translocase FANCM/MHF promotes replication traverse of DNA interstrand crosslinks. Mol. Cell *52*, 434–446.

Huntoon, C.J., Flatten, K.S., Wahner Hendrickson, A.E., Huehls, A.M., Sutor, S.L., Kaufmann, S.H., and Karnitz, L.M. (2013). ATR inhibition broadly sensitizes ovarian cancer cells to chemotherapy independent of BRCA status. Cancer Res. *73*, 3683–3691.

Jackson, D.A., and Pombo, A. (1998). Replicon clusters are stable units of chromosome structure: evidence that nuclear organization contributes to the efficient activation and propagation of S phase in human cells. J. Cell Biol. *140*, 1285–1295.

Jansen, J.G., Temviriyanukul, P., Wit, N., Delbos, F., Reynaud, C.A., Jacobs, H., and de Wind, N. (2014). Redundancy of mammalian Y family DNA polymerases in cellular responses to genomic DNA lesions induced by ultraviolet light. Nucleic Acids Res. *42*, 11071–11082.

Jazaeri, A.A., Shibata, E., Park, J., Bryant, J.L., Conaway, M.R., Modesitt, S.C., Smith, P.G., Milhollen, M.A., Berger, A.J., and Dutta, A. (2013). Overcoming platinum resistance in preclinical models of ovarian cancer using the neddylation inhibitor MLN4924. Mol. Cancer Ther. *12*, 1958–1967.

Jones, J.C., Zhen, W.P., Reed, E., Parker, R.J., Sancar, A., and Bohr, V.A. (1991). Gene-specific formation and repair of cisplatin intrastrand adducts and interstrand cross-links in Chinese hamster ovary cells. J. Biol. Chem. *266*, 7101–7107.

Karnitz, L.M., and Zou, L. (2015). Molecular Pathways: Targeting ATR in Cancer Therapy. Clin. Cancer Res *21*, 4780–4785.

Keen, B.A., Bailey, L.J., Jozwiakowski, S.K., and Doherty, A.J. (2014a). Human PrimPol mutation associated with high myopia has a DNA replication defect. Nucleic Acids Res. *42*, 12102–12111.

Keen, B.A., Jozwiakowski, S.K., Bailey, L.J., Bianchi, J., and Doherty, A.J. (2014b). Molecular dissection of the domain architecture and catalytic activities of human PrimPol. Nucleic Acids Res. *42*, 5830–5845.

Keka, I.S., Mohiuddin, Maede, Y., Rahman, M.M., Sakuma, T., Honma, M., Yamamoto, T., Takeda, S., and Sasanuma, H. (2015). Smarcal1 promotes double-strand-break repair by nonhomologous end-joining. Nucleic Acids Res. *43*, 6359–6372.

Kim, D., Liu, Y., Oberly, S., Freire, R., and Smolka, M.B. (2018). ATR-mediated proteome remodeling is a major determinant of homologous recombination capacity in cancer cells. Nucleic Acids Res. *46*, 8311–8325.

King, M.C., Marks, J.H., and Mandell, J.B.; New York Breast Cancer Study Group (2003). Breast and ovarian cancer risks due to inherited mutations in BRCA1 and BRCA2. Science *302*, 643–646.

Kobayashi, K., Guilliam, T.A., Tsuda, M., Yamamoto, J., Bailey, L.J., Iwai, S., Takeda, S., Doherty, A.J., and Hirota, K. (2016). Repriming by PrimPol is critical for DNA replication restart downstream of lesions and chain-terminating nucleosides. Cell Cycle *15*, 1997–2008.

Kojic, A., Cuadrado, A., De Koninck, M., Giménez-Llorente, D., Rodríguez-Corsino, M., Gómez-López, G., Le Dily, F., Marti-Renom, M.A., and Losada, A. (2018). Distinct roles of cohesin-SA1 and cohesin-SA2 in 3D chromosome organization. Nat. Struct. Mol. Biol. *25*, 496–504.

Kolinjivadi, A.M., Sannino, V., De Antoni, A., Zadorozhny, K., Kilkenny, M., Techer, H., Baldi, G., Shen, R., Ciccia, A., Pellegrini, L., et al. (2017). Smarcal1-Mediated Fork Reversal Triggers Mre11-Dependent Degradation

of Nascent DNA in the Absence of Brca2 and Stable Rad51 Nucleofilaments. Mol. Cell *67*, 867–881.e7.

Lemaçon, D., Jackson, J., Quinet, A., Brickner, J.R., Li, S., Yazinski, S., You, Z., Ira, G., Zou, L., Mosammaparast, N., and Vindigni, A. (2017). MRE11 and EXO1 nucleases degrade reversed forks and elicit MUS81-dependent fork rescue in BRCA2-deficient cells. Nat. Commun. *8*, 860.

Lerner, L.K., Francisco, G., Soltys, D.T., Rocha, C.R., Quinet, A., Vessoni, A.T., Castro, L.P., David, T.I., Bustos, S.O., Strauss, B.E., et al. (2017). Predominant role of DNA polymerase eta and p53-dependent translesion synthesis in the survival of ultraviolet-irradiated human cells. Nucleic Acids Res. *45*, 1270–1280.

Lohse, I., Borgida, A., Cao, P., Cheung, M., Pintilie, M., Bianco, T., Holter, S., Ibrahimov, E., Kumareswaran, R., Bristow, R.G., et al. (2015). BRCA1 and BRCA2 mutations sensitize to chemotherapy in patient-derived pancreatic cancer xenografts. Br. J. Cancer *113*, 425–432.

Méndez, J., and Stillman, B. (2000). Chromatin association of human origin recognition complex, cdc6, and minichromosome maintenance proteins during the cell cycle: assembly of prereplication complexes in late mitosis. Mol. Cell. Biol. *20*, 8602–8612.

Metcalfe, K., Lubinski, J., Lynch, H.T., Ghadirian, P., Foulkes, W.D., Kim-Sing, C., Neuhausen, S., Tung, N., Rosen, B., Gronwald, J., et al.; Hereditary Breast Cancer Clinical Study Group (2010). Family history of cancer and cancer risks in women with BRCA1 or BRCA2 mutations. J. Natl. Cancer Inst. *102*, 1874–1878.

Mijic, S., Zellweger, R., Chappidi, N., Berti, M., Jacobs, K., Mutreja, K., Ursich, S., Ray Chaudhuri, A., Nussenzweig, A., Janscak, P., and Lopes, M. (2017). Replication fork reversal triggers fork degradation in BRCA2-defective cells. Nat. Commun. *8*, 859.

Mourón, S., Rodriguez-Acebes, S., Martínez-Jiménez, M.I., García-Gómez, S., Chocrón, S., Blanco, L., and Méndez, J. (2013). Repriming of DNA synthesis at stalled replication forks by human PrimPol. Nat. Struct. Mol. Biol. *20*, 1383–1389.

Mutreja, K., Krietsch, J., Hess, J., Ursich, S., Berti, M., Roessler, F.K., Zellweger, R., Patra, M., Gasser, G., and Lopes, M. (2018). ATR-Mediated Global Fork Slowing and Reversal Assist Fork Traverse and Prevent Chromosomal Breakage at DNA Interstrand Cross-Links. Cell Rep *24*, 2629–2642.e5.

Neelsen, K.J., and Lopes, M. (2015). Replication fork reversal in eukaryotes: from dead end to dynamic response. Nat. Rev. Mol. Cell Biol. *16*, 207–220.

Neelsen, K.J., Chaudhuri, A.R., Follonier, C., Herrador, R., and Lopes, M. (2014). Visualization and interpretation of eukaryotic DNA replication intermediates in vivo by electron microscopy. Methods Mol. Biol. *1094*, 177–208.

Nielsen, F.C., van Overeem Hansen, T., and Sørensen, C.S. (2016). Hereditary breast and ovarian cancer: new genes in confined pathways. Nat. Rev. Cancer *16*, 599–612.

Olivieri, G., Bodycote, J., and Wolff, S. (1984). Adaptive response of human lymphocytes to low concentrations of radioactive thymidine. Science *223*, 594–597.

Pilzecker, B., Buoninfante, O.A., Pritchard, C., Blomberg, O.S., Huijbers, I.J., van den Berk, P.C., and Jacobs, H. (2016). PrimPol prevents APOBEC/AID family mediated DNA mutagenesis. Nucleic Acids Res. *44*, 4734–4744.

Poklar, N., Pilch, D.S., Lippard, S.J., Redding, E.A., Dunham, S.U., and Breslauer, K.J. (1996). Influence of cisplatin intrastrand crosslinking on the conformation, thermal stability, and energetics of a 20-mer DNA duplex. Proc. Natl. Acad. Sci. USA *93*, 7606–7611.

Quinet, A., Vessoni, A.T., Rocha, C.R., Gottifredi, V., Biard, D., Sarasin, A., Menck, C.F., and Stary, A. (2014). Gap-filling and bypass at the replication fork are both active mechanisms for tolerance of low-dose ultraviolet-induced DNA damage in the human genome. DNA Repair (Amst.) *14*, 27–38.

Quinet, A., Martins, D.J., Vessoni, A.T., Biard, D., Sarasin, A., Stary, A., and Menck, C.F. (2016). Translesion synthesis mechanisms depend on the nature of DNA damage in UV-irradiated human cells. Nucleic Acids Res. *44*, 5717–5731.

Quinet, A., Carvajal-Maldonado, D., Lemacon, D., and Vindigni, A. (2017a). DNA Fiber Analysis: Mind the Gap! Methods Enzymol. *591*, 55–82.

Quinet, A., Lemaçon, D., and Vindigni, A. (2017b). Replication Fork Reversal: Players and Guardians. Mol. Cell *68*, 830–833.

Quinet, A., Lerner, L.K., Martins, D.J., and Menck, C.F.M. (2018). Filling gaps in translesion DNA synthesis in human cells. Mutat. Res. Genet. Toxicol. Environ. Mutagen. *836* (Pt B), 127–142.

Ray Chaudhuri, A., Callen, E., Ding, X., Gogola, E., Duarte, A.A., Lee, J.E., Wong, N., Lafarga, V., Calvo, J.A., Panzarino, N.J., et al. (2016). Replication fork stability confers chemoresistance in BRCA-deficient cells. Nature *535*, 382–387.

Reaper, P.M., Griffiths, M.R., Long, J.M., Charrier, J.D., Maccormick, S., Charlton, P.A., Golec, J.M., and Pollard, J.R. (2011). Selective killing of ATM- or p53-deficient cancer cells through inhibition of ATR. Nat. Chem. Biol. *7*, 428–430.

Rechkoblit, O., Gupta, Y.K., Malik, R., Rajashankar, K.R., Johnson, R.E., Prakash, L., Prakash, S., and Aggarwal, A.K. (2016). Structure and mechanism of human PrimPol, a DNA polymerase with primase activity. Sci. Adv. *2*, e1601317.

Rocha, C.R., Garcia, C.C., Vieira, D.B., Quinet, A., de Andrade-Lima, L.C., Munford, V., Belizário, J.E., and Menck, C.F. (2014). Glutathione depletion sensitizes cisplatin- and temozolomide-resistant glioma cells in vitro and in vivo. Cell Death Dis. *5*, e1505.

Romanello, M., Schiavone, D., Frey, A., and Sale, J.E. (2016). Histone H3.3 promotes IgV gene diversification by enhancing formation of AID-accessible single-stranded DNA. EMBO J. *35*, 1452–1464.

Rupp, W.D., and Howard-Flanders, P. (1968). Discontinuities in the DNA synthesized in an excision-defective strain of Escherichia coli following ultraviolet irradiation. J. Mol. Biol. *31*, 291–304.

Saldivar, J.C., Cortez, D., and Cimprich, K.A. (2017). The essential kinase ATR: ensuring faithful duplication of a challenging genome. Nat. Rev. Mol. Cell Biol. *18*, 622–636.

Sale, J.E. (2013). Translesion DNA synthesis and mutagenesis in eukaryotes. Cold Spring Harb. Perspect. Biol. *5*, a012708.

Sarasin, A.R., and Hanawalt, P.C. (1978). Carcinogens enhance survival of UV-irradiated simian virus 40 in treated monkey kidney cells: induction of a recovery pathway? Proc. Natl. Acad. Sci. USA *75*, 346–350.

Schiavone, D., Jozwiakowski, S.K., Romanello, M., Guilbaud, G., Guilliam, T.A., Bailey, L.J., Sale, J.E., and Doherty, A.J. (2016). PrimPol Is Required for Replicative Tolerance of G Quadruplexes in Vertebrate Cells. Mol. Cell *61*, 161–169.

Schlacher, K., Christ, N., Siaud, N., Egashira, A., Wu, H., and Jasin, M. (2011). Double-strand break repair-independent role for BRCA2 in blocking stalled replication fork degradation by MRE11. Cell *145*, 529–542.

Schlacher, K., Wu, H., and Jasin, M. (2012). A distinct replication fork protection pathway connects Fanconi anemia tumor suppressors to RAD51-BRCA1/2. Cancer Cell *22*, 106–116.

Simpson, L.J., and Sale, J.E. (2003). Rev1 is essential for DNA damage tolerance and non-templated immunoglobulin gene mutation in a vertebrate cell line. EMBO J. *22*, 1654–1664.

Šviković, S., Crisp, A., Tan-Wong, S.M., Guilliam, T.A., Doherty, A.J., Proudfoot, N.J., Guilbaud, G., and Sale, J.E. (2019). R-loop formation during S phase is restricted by PrimPol-mediated repriming. EMBO J. *38*, e99793.

Taglialatela, A., Alvarez, S., Leuzzi, G., Sannino, V., Ranjha, L., Huang, J.W., Madubata, C., Anand, R., Levy, B., Rabadan, R., et al. (2017). Restoration of Replication Fork Stability in BRCA1- and BRCA2-Deficient Cells by Inactivation of SNF2-Family Fork Remodelers. Mol. Cell *68*, 414–430.e8.

Taniguchi, T., Tischkowitz, M., Ameziane, N., Hodgson, S.V., Mathew, C.G., Joenje, H., Mok, S.C., and D'Andrea, A.D. (2003). Disruption of the Fanconi anemia-BRCA pathway in cisplatin-sensitive ovarian tumors. Nat. Med. *9*, 568–574.

Thangavel, S., Berti, M., Levikova, M., Pinto, C., Gomathinayagam, S., Vujanovic, M., Zellweger, R., Moore, H., Lee, E.H., Hendrickson, E.A., et al.

(2015). DNA2 drives processing and restart of reversed replication forks in human cells. J. Cell Biol. *208*, 545–562.

Tilby, M.J., Johnson, C., Knox, R.J., Cordell, J., Roberts, J.J., and Dean, C.J. (1991). Sensitive detection of DNA modifications induced by cisplatin and carboplatin in vitro and in vivo using a monoclonal antibody. Cancer Res. *51*, 123–129.

Vaisman, A., and Woodgate, R. (2017). Translesion DNA polymerases in eukaryotes: what makes them tick? Crit. Rev. Biochem. Mol. Biol. *52*, 274–303.

Vallerga, M.B., Mansilla, S.F., Federico, M.B., Bertolin, A.P., and Gottifredi, V. (2015). Rad51 recombinase prevents Mre11 nuclease-dependent degradation and excessive PrimPol-mediated elongation of nascent DNA after UV irradiation. Proc. Natl. Acad. Sci. USA *112*, E6624–E6633.

Vindigni, A., and Lopes, M. (2017). Combining electron microscopy with single molecule DNA fiber approaches to study DNA replication dynamics. Biophys. Chem. *225*, 3–9.

Wan, L., Lou, J., Xia, Y., Su, B., Liu, T., Cui, J., Sun, Y., Lou, H., and Huang, J. (2013). hPrimpol1/CCDC111 is a human DNA primase-polymerase required for the maintenance of genome integrity. EMBO Rep. *14*, 1104–1112.

Yazinski, S.A., Comaills, V., Buisson, R., Genois, M.M., Nguyen, H.D., Ho, C.K., Todorova Kwan, T., Morris, R., Lauffer, S., Nussenzweig, A., et al. (2017). ATR inhibition disrupts rewired homologous recombination and fork protection pathways in PARP inhibitor-resistant BRCA-deficient cancer cells. Genes Dev. *31*, 318–332.

Yeeles, J.T., Poli, J., Marians, K.J., and Pasero, P. (2013). Rescuing stalled or damaged replication forks. Cold Spring Harb. Perspect. Biol. *5*, a012815.

Ying, S., Hamdy, F.C., and Helleday, T. (2012). Mre11-dependent degradation of stalled DNA replication forks is prevented by BRCA2 and PARP1. Cancer Res. *72*, 2814–2821.

Zellweger, R., Dalcher, D., Mutreja, K., Berti, M., Schmid, J.A., Herrador, R., Vindigni, A., and Lopes, M. (2015). Rad51-mediated replication fork reversal is a global response to genotoxic treatments in human cells. J. Cell Biol. *208*, 563–579.

STAR★METHODS

KEY RESOURCES TABLE

REAGENT or RESOURCE	SOURCE	IDENTIFIER
Antibodies		
Mouse monoclonal anti-BrdU (B44)	BD Biosciences	Cat# 347580; RRID: AB_400326
Rat monoclonal anti-BrdU [BU1/75 (ICR1)]	Abcam	Cat# ab6326; RRID: AB_305426
Rabbit polyclonal anti-PRIMPOL	Mourón et al., 2013	N/A
Mouse monoclonal IgG_{2a} anti-CHK1 (G-4)	Santa Cruz Biotechnology	Cat# Sc-8408; RRID: AB_627257
Rabbit monoclonal anti-Phospho-CHK1 (Ser345) (133D3)	Cell Signaling Technology	Cat# 2348; RRID: AB_331212
Mouse monoclonal IgG_1 anti-SMARCAL1 (E-12)	Santa Cruz Biotechnology	Cat# Sc-166209; RRID: AB_2191695
Mouse monoclonal anti-BRCA1 (Ab-2) (MS13)	Millipore Sigma	Cat# OP93; RRID: AB_213440
Mouse monoclonal anti-BRCA2 (Ab-1) (2B)	Millipore Sigma	Cat#OP95; RRID: AB_213443
Mouse monoclonal IgG_{2a} anti-V5	Thermo Fisher Scientific	Cat# R96025; RRID: AB_2556564
Rabbit polyclonal IgG anti-RAD51 (H-92)	Santa Cruz Biotechnology	Cat# Sc-8349; RRID: AB_2253533
Rabbit polyclonal anti-RAD51 (Ab-1)	Millipore Sigma	Cat# PC130; RRID: AB_2238184
Rat monoclonal anti-Cisplatin-modified DNA [CP9/19]	Abcam	Cat# ab103261; RRID: AB_10715243
Mouse monoclonal anti-RPA32/RPA2 [9H8]	Abcam	Cat# ab2175; RRID: AB_302873
Rabbit polyclonal anti-Phospho-RPA32 (S33)	Bethyl	Cat# A300-246A; RRID: AB_2180847
Mouse monoclonal anti-Phospho-Histone H2A.X (Ser139) (clone JBW301)	Millipore Sigma	Cat# 05-636; RRID: AB_2755003
Rabbit polyclonal anti-Histone H2A.X	Cell Signaling Technology	Cat# 2595; RRID: AB_10694556
Rabbit polyclonal anti-SMC1	Ana Losada (Kojic et al., 2018)	N/A
Mouse monoclonal IgG_{2a} anti-PCNA (F-2)	Santa Cruz Biotechnology	Cat# Sc-25280; RRID: AB_628109
Mouse monoclonal anti-PCNA [PC10]	Abcam	Cat# Ab29; RRID: AB_303394
Rabbit monoclonal anti-GAPDH [EPR1689]	Abcam	Cat# ab181602; RRID: AB_2630358
Mouse monoclonal anti-TUBULIN	Millipore Sigma	Cat# T5168; RRID: AB_477579
Mouse monoclonal anti-alpha-TUBULIN	Millipore Sigma	Cat# F2168; RRID: AB_476967
Alexa Fluor 488 Chicken anti-Rat IgG (H+L)	Thermo Fisher Scientific	Cat# A21470; RRID: AB_2535873
Alexa Fluor 546 Goat anti-Mouse IgG_1	Thermo Fisher Scientific	Cat# A21123; RRID: AB_2535765
Alexa Fluor 594 Goat anti-Mouse IgG (H+L)	Thermo Fisher Scientific	Cat# A11005; RRID: AB_2534073
Alexa Fluor 488 Goat anti-Rabbit IgG (H+L)	Thermo Fisher Scientific	Cat# A11034; RRID: AB_2576217
IRDye 800CW Goat anti-Rabbit IgG (H+L)	LI-COR Biosciences	Cat# 925-32211; RRID: AB_2651127
IRDye 680RD Goat anti-Mouse IgG (H + L)	LI-COR Biosciences	Cat# 925-68070; RRID: AB_2651128
Goat anti-Mouse IgG (H+L), HRP	Thermo Fisher Scientific	Cat# 62-6520; RRID: AB_2533947
Goat anti-Rabbit IgG (H+L), HRP	Thermo Fisher Scientific	Cat# PI31460; RRID: AB_228341
Sheep anti-Mouse IgG (H+L), HRP	GE Healthcare	Cat# NA931; RRID: AB_772210
Donkey anti-Rabbit IgG (H+L), HRP	GE Healthcare	Cat# NA934, RRID: AB_772206
Bacterial and Virus Strains		
One Shot® TOP10 Chemically Competent *E. coli*	Thermo Fisher Scientific	Cat# C404003
Chemicals, Peptides, and Recombinant Proteins		
Cisplatin	Millipore Sigma	Cat# P4394-250MG
Hydroxyurea	Millipore Sigma	Cat# H8627-5G

(*Continued on next page*)

Continued

REAGENT or RESOURCE	SOURCE	IDENTIFIER
Olaparib	Selleck Chemicals	Cat# S1060
Mirin	Millipore Sigma	Cat# M9948-5MG
VE-821 (ATRi)	Selleck Chemicals	Cat# S8007
IdU	Millipore Sigma	Cat# I7125-5G
CldU	Millipore Sigma	Cat# C6891-100MG
EdU	Thermo Fisher Scientific	Cat# A10044
DAPI	Millipore Sigma	Cat# D9542
RNAimax transfection reagent	Thermo Fisher Scientific	Cat# 13778-150
TransIT LT1 transfection reagent	Mirus	Cat# MIR 2300
Lipofectamine 2000 transfection reagent	Thermo Fisher Scientific	Cat# 11668019
S1 nuclease	Thermo Fisher Scientific	Cat# 18001-016
Saponin	Millipore Sigma	Cat# 8047-15-2
PureLink RNase A (20 mg/mL)	Thermo Fisher Scientific	Cat# 12091039
Benzonase Nuclease HC, Purity > 99%	Millipore Sigma	Cat# 71206
Pierce Protease inhibitor tablets, EDTA-free	Thermo Fisher Scientific	Cat# A32965
PhosSTOP	Millipore Sigma	Cat# 04 906 837 001
G 418 disulfate salt solution	Millipore Sigma	Cat# G8168
Doxycycline	Clontech	Cat# 631311
TMP (4,5′,8-Trimethylpsoralen)	Millipore Sigma	Cat# T6137-100MG
Proteinase K	Life Technologies	Cat# 25530-015
PvuII HF	New England Biolabs	Cat# R3151S
Benzoylated Naphthoylated DEAE-Cellulose	Millipore Sigma	Cat# B6385-25G
Benzalkonium chloride (BAC)	Millipore Sigma	Cat# B6295
Uranyl Acetate	Electron Microscopy Sciences	Cat# 541-09-3
Critical Commercial Assays		
Click-It EdU Alexa Fluor 488 Imaging Kit	Thermo Fisher Scientific	Cat# C10337
Cell proliferation Kit II (XTT)	Millipore Sigma	Cat# 11465015001
PureLink RNA mini Kit	Thermo Fisher Scientific	Cat# 12183018A
M-MLV Reverse Transcriptase	Thermo Fisher Scientific	Cat# 28025013
iQTM SYBR Green supermix	Biorad	Cat# 41708880
Pierce BCA Protein Assay Kit	Thermo Fisher Scientific	Cat# 23225
Deposited Data		
Unprocessed microscopy images, gels, and blots	This paper, Mendeley data	https://doi.org/10.17632/c9dn6snk6w.1
Experimental Models: Cell Lines		
Human: U2OS	ATCC	HTB-96
Human: Stable U2OS doxycycline-inducible shPrimPol	Mourón et al., 2013	N/A
Human: PRIMPOL KO U2OS (clone 1D11)	This study	N/A
Human: UWB1.289	ATCC	CRL-2945
Human: UWB1.289 + BRCA1	ATCC	CRL-2946
Human: UWB1.289 + PRIMPOL	This study	N/A
Human: HEK293T	Luis Batista lab	N/A
Chicken: DT40 wild type	Sale lab stock	N/A
Chicken: DT40 *primpol* −/−	Šviković et al., 2019	N/A
Chicken: DT40 *smarcal1* +/−	This study	N/A

(*Continued on next page*)

Continued

REAGENT or RESOURCE	SOURCE	IDENTIFIER
Chicken: DT40 *smarcal1* –/–	Keka et al., 2015	N/A
Chicken: DT40 *primpol* –/– *smarcal1* +/–	This study	N/A
Oligonucleotides		
Gg SMARCAL1 qPCR primer F:	This study	TTACGTGGGCAGAGGCATTT
Gg SMARCAL1 qPCR primer R:	This study	TGCTGAGGAGGTCAAAGCTG
Gg EF1 alpha housekeeping gene primer F:	Romanello et al., 2016	GGTTATGCCCCTGTGCTGGATT
Gg EF1 alpha housekeeping gene primer R:	Romanello et al., 2016	CTTCTTGTCGACGGCCTTGATGA
Gg SMARCAL1 intron primer F:	Keka et al., 2015	TAAAAAATGGACGTTTACTTCTT GCGGATG
Gg SMARCAL1 intron primer R:	Keka et al., 2015	AATAACTACTTGGAAAGTGCTCTTG AGTTG
Gg SMARCAL1 5′HA genotyping primer F:	This study	TGTTTAACTTGAAAACAAAGGGAGG
Gg SMARCAL1 3′HA genotyping primer R:	This study	AAATATGAGAGCTAGTTTGTGC
Blasticidin S genotyping primer F:	This study	TGGTTACAAATAAAGCAATAGC
Chicken beta actin genotyping primer R:	This study	ATAAATACAAAATTGGGGGTGG
SMARCAL1 RT-qPCR primer F:	This study	CAGAGGCAGACCTTTCTGAAG
SMARCAL1 RT-qPCR primer R:	This study	CGGCCTCCTTTGGCTATG
BRCA1 RT-qPCR primer F:	Lemaçon et al., 2017	AGAAACCACCAAGGTCCAAAG
BRCA1 RT-qPCR primer R:	Lemaçon et al., 2017	GGGCCCATAGCAACAGATTT
BRCA2 RT-qPCR primer F:	Lemaçon et al., 2017	TGCAGCAATTAACATATGAGG
BRCA2 RT-qPCR primer R:	Lemaçon et al., 2017	AGGACTTGCCCCTTTCGTCTA
RAD51 RT-qPCR primer F:	This study	GAAGACCCAGATCTGTCATACG
RAD51 RT-qPCR primer R:	This study	GTGTCAATGTACATGGCCTTTC
REV3L RT-qPCR primer F:	This study	TCATGAGAAGGAAAGACACTTTATG
REV3L RT-qPCR primer R:	This study	GCTGTAGGAGGTAGGGAATATG
POLH RT-qPCR primer F:	Christmann et al., 2016	ATCTTCTACTGGCACAAG
POLH RT-qPCR primer R:	Christmann et al., 2016	ACATTATCTCCATCACTTCA
PrimPol RT-qPCR primer F:	Vallerga et al., 2015	TTCTACTGAAGTGCCGATACTGT
PrimPol RT-qPCR primer R:	Vallerga et al., 2015	TGTGGCTTTGGAGGTTACTGA
Beta-Actin RT-qPCR primer F:	This study	CTCGCCTTTGCCGATCC
Beta-Actin RT-qPCR primer R:	This study	ATGCCGGAGCCGTTGTC
REV1 RT-qPCR primer F:	Quinet et al., 2016	CCCAGACATCAGAGCTGTATAAT
REV1 RT-qPCR primer R:	Quinet et al., 2016	CTTCCTGTGCCTCTGTTACTT
Silencer Select Negative control #1 siRNA (siCT)	Ambion	Cat# 4390843
siRNA BRCA1	Dharmacon	Cat# L-007287
siRNA PRIMPOL	Dharmacon (custom siRNA)	GAGGAAACCGUUGUCCUCAGUGUAU
siRNA RAD51	Thermo Fisher Scientific	Cat# VHS40453
siRNA SMARCAL1	Dharmacon	Cat# D-013058-04-0002
siRNA BRCA2	Dharmacon	Cat# L-003462
sgRNA for PRIMPOL KO	This study	GATAGCGCTCCAGAGACAACNGG
Recombinant DNA		
Plasmid: *SMARCAL1* puromycin targeting construct	Keka et al., 2015	N/A
Plasmid: *SMARCAL1* blasticidin S targeting construct	This study	N/A
Plasmid: pCDH-CMV-MCS-EF1α-Neo	System Biosciences	Cat# CD514B-1
Plasmid: pCDH-CMV-WT-PRIMPOL-EF1α-Neo (WT PRIMPOL)	This study	N/A

(*Continued on next page*)

Continued		
REAGENT or RESOURCE	SOURCE	IDENTIFIER
Plasmid: pcDNA3.1_nV5-DEST V5-WT-PRIMPOL (V5-WT-PRIMPOL)	Mourón et al., 2013	N/A
Plasmid: pcDNA3.1_nV5-DEST V5-AxA-PRIMPOL (V5-AxA-PRIMPOL)	Mourón et al., 2013	N/A
Plasmid: pcDNA3.1_nV5-DEST V5-CH-PRIMPOL (V5-CH-PRIMPOL)	Mourón et al., 2013	N/A
Plasmid: pRP[Exp]-CMV > gag:pol:RRE	Vector Builder	Cat# VB160226-10009
pRP[Exp]-CMV > VSVG	Vector Builder	Cat# VB160226-10010
pRP[Exp]-RSV > Rev	Vector Builder	Cat# VB160226-10011
Software and Algorithms		
ImageJ		https://imagej.nih.gov/ij/ RRID: SCR_003070
GraphPad Prism	GraphPad software	https://www.graphpad.com RRID:SCR_002798
FlowJo	FlowJo LLC	https://www.flowjo.com/ RRID: SCR_008520
ImageStudioLite2	LiCOR Odyssey	https://www.licor.com/bio/image-studio-lite/ RRID: SCR_013715
LAS (Leica Application Suite) AF software	Leica	https://www.leica-microsystems.com/products/microscope-software/
Other		
Lenti-X Concentrator	Clontech	Cat# 631232
MEGM Mammary Epithelial Cell Growth Medium BulletKit	Lonza	Cat# CC-3150
JEOL 1200 EX Electron Microscope	JEOL	N/A
AMTXR41 Camera	AMT	N/A
Leica EM ACE600 Coater	Leica	N/A
TCS SP5 confocal microscope	Leica	N/A
Odyssey CLx Imaging System	LI-COR Biosciences	N/A
FACSCanto II	BD Biosciences	N/A
UVP CL-1000L Crosslinker	Fisher Scientific	Cat# UVP95017401
UVC Bulb (UVP XX-15S, Bench lamp, 254 nm)	MidSci	Cat# UVP95004205
UVP UV Radiometer	MidSci	Cat# UVP97001502
UVP UVC Sensor (254 nm)	MidSci	Cat# UVP97001601

LEAD CONTACT AND MATERIAL AVAILABILITY

Further information and request for resources and reagents should be directed to and will be fulfilled by the Lead Contact, Alessandro Vindigni (avindigni@wustl.edu). All unique/stable reagents generated in this study are available from the Lead Contact without restriction.

EXPERIMENTAL MODELS AND SUBJECT DETAILS

Cell culture and cell lines

The *BRCA1* mutant ovarian cancer cells UWB1.289 (named UW for simplification purposes) and their complemented derivative expressing wild-type BRCA1, UWB1.289+BRCA1 (named UW+BRCA1 for simplification purposes) (provided by Dr. Lee Zou, Harvard Medical School) (Yazinski et al., 2017; Lemaçon et al., 2017), were cultivated in 50% RPMI media, 50% MEGM BulletKit (Lonza CC-3150) supplemented with 3% FBS, 100 U/mL penicillin, and 100 μg/mL streptomycin at 37°C, 5% CO_2. For the culture of UW+BRCA1 cells, 400 μg/mL of G418 (G8168, Millipore Sigma) were added to the media. The human osteosarcoma U2OS cells (American Type Culture Collection), the U2OS cells stably expressing a doxycycline-inducible shRNA targeting *PRIMPOL*

(Mourón et al., 2013), and the PRIMPOL KO U2OS cells were grown in DMEM media supplemented with 10% FBS, 100 U/mL penicillin, and 100 μg/mL streptomycin at 37°C, 5% CO2.

Generation of a stable cell line overexpressing PRIMPOL

To establish the UW cells stably overexpressing WT-PRIMPOL (UW+PRIMPOL), V5-WT-PRIMPOL was PCR-cloned from pcDNA3.1/nV5-DEST Gateway Vector (Mourón et al., 2013) into the XbaI-AsiSI site of the lentivector pCDH_MCS_EF1_NEO mammalian expression system (System Biosciences). This plasmid was co-transfected with helper plasmids into HEK293T cells using Lipofectamine 2000 (ThermoFisher Scientific). Virus supernatant was collected, filtered (0.45 μm Cellulose Acetate) and concentrated using Lenti-X Concentrator (Clontech). Transduced cells were selected with and cultivated in the presence of 400 μg/mL of G418.

Generation of PRIMPOL KO cells

PRIMPOL KO U2OS cells were engineered by the Genome Engineering and IPSC Center (GEiC) of the Washington University in St. Louis Cells. Briefly, CRISPR/Cas9 was used to induce cleavage of Exon 7 of PRIMPOL with a gRNA sequence of 5′ -GATAG CGCTCCAGAGACAAC-NGG-3′. After clonal expansion, next generation sequencing was used to identify a clone bearing insertion (+1) and deletions (−1 or −7) on the three alleles of the PRIMPOL gene in U2OS cell line.

METHOD DETAILS

Gene silencing with RNAi

Transient gene depletions were carried out using the Lipofectamine RNAiMax transfection reagent (Life Technologies), according to the manufacturer's instructions and the following siRNA at a final concentration of 50 nM: SMARTpool siRNA L-003461-00 (Dharmacon) for BRCA1 (Lemaçon et al., 2017), custom-made 5′-GAG GAA ACC GUU GUC CUC AGU GUA U-3′ (Dharmacon) for PRIMPOL (Vallerga et al., 2015) and VHS40453 (Thermo Fisher Scientific) for RAD51 (Thangavel et al., 2015; Lemaçon et al., 2017). Depletion of SMARCAL1 was performed with 20 nM siGENOME individual siRNA (D-013058-04-02, Dharmacon) (Carvajal-Maldonado et al., 2019; Taglialatela et al., 2017) and of BRCA2 with 10 nM SMARTpool siRNA (L-003462-00, Dharmacon) (Carvajal-Maldonado et al., 2019; Lemaçon et al., 2017). Silencer select negative control #1 siRNA (4390843, Ambion) was used as control siRNA (siCT) at the same concentration of the most concentrated siRNA used in the same experiment (Lemaçon et al., 2017).

The induction of shRNA targeting PRIMPOL (shPRIMPOL) in U2OS cells stably expressing a doxycycline-inducible shPRIMPOL was carried out by adding 1 μg/mL doxycycline to the cell growth media for 3 days (Mourón et al., 2013).

Transfections of PRIMPOL constructs

Different PRIMPOL constructs (WT, wild-type; AxA, catalytically dead and CH, primase dead) (Mourón et al., 2013) were transiently overexpressed in UW cells upon transfection of the corresponding plasmids using Transit-LT1 Transfection Reagent (MIR 2304, Mirus) and experiments were performed 48 h after transfection.

Drugs and cell treatments

Cisplatin (P4394, Millipore Sigma) was dissolved in 10X PBS at a 5 mM concentration stock and stored at −20°C. Prior to use, aliquots were warmed at 60°C for approximately 10 min and then diluted in growth media to the indicated final concentrations. For the single challenging dose experiments, cells were treated with 150 μM cisplatin for 1 h at 37°C, 5% CO_2. For the multiple dose experiments, cells received a 50 μM pre-dose of cisplatin for 1 h at 37°C, 5% CO_2, washed twice with PBS, and allowed to recover in fresh media in the incubator. Twenty-four h after the pre-dose treatment, cells were treated with 150 μM cisplatin for 1 h (challenging dose) at 37°C, 5% CO_2, washed twice with PBS, and harvested for subsequent analysis. For chronic treatment with cisplatin, cells were placed in growth media with the indicated final concentrations of cisplatin for the entire duration of the experiment.

For UVC irradiation, cells were washed with warmed (37°C) PBS and then exposed to a UVC lamp (XX-15S, Bench lamp, 254 nm, 95-0042-05, UVP) at a rate of 1 J/m^2/s as monitored by a UV radiometer and UVC sensor (97-0015-02 and 97-0016-01, UVP) for 10 s for a final dose of 10 J/m^2 or 30 s for 30 J/m^2.

Hydroxyurea (HU, Millipore Sigma H8627) was dissolved in water at a 1 M concentration stock and dissolved in cell growth media to a final concentration of 1 mM (for pre-dose treatment) or 4 mM (for challenging dose treatment). Treatments with HU were performed for 2 h at 37°C, 5% CO_2.

The MRE11 inhibitor mirin (M9948, Millipore Sigma) was dissolved in DMSO at a 50 mM concentration stock and dissolved in cell growth media to a final concentration of 50 μM. The ATR inhibitor VE-821 (S8007, Selleckchem) was dissolved in DMSO at a 10 mM concentration stock and dissolved in cell growth media to a final concentration that ranged between 62.5 and 1000 nM. The PARP inhibitor Olaparib (AZD2281, Selleckchem) was dissolved in DMSO for a 10 mM concentration stock and immediately dissolved in cell growth media to a final concentration of 375 nM for cell survival experiments or 10 μM for DNA fiber experiments.

Cell survival

Cell survival assay was performed using Cell Proliferation Kit II (XTT, 11465015001, Millipore Sigma) by seeding $1.3x10^4$ cells per well in a 24-well plate in duplicate the day prior to treatment. Cells were then treated chronically with the indicated doses of cisplatin, PARPi (olaparib), or ATRi (VE-821) and cell survival was assessed after 6 days of treatment for UW ± BRCA1 and UW+PRIMPOL ovarian cancer cells, and after 4 days of treatment for U2OS cells. The absorbance was measured at 450 nm with a reference wavelength at 650 nm. Results were expressed as percentage of the corresponding untreated control (Quinet et al., 2014).

For colony assays, the following concentrations of cells were plated in 60 mm plates: 4000 UW+BRCA1 or UW cells transfected with siCT or siPRIMPOL; 300, 600, and 1200 cells U2OS cells transfected with siCT ± doxycycline for shPRIMPOL induction; 1500, 2100, and 3000 U2OS cells transfected with siBRCA1 ± shPRIMPOL. Media was changed every 3-4 days. Cells were fixed 14 days after plating with 10% acetic acid / 10% methanol and stained with 0.4% crystal violet in 20% ethanol for about 1 h. Plates were rinsed with water and dried overnight. Only clearly distinguishable colonies were counted. Differences in initial cell plating were taken into account for the calculation of survival fraction relative to the corresponding control (set as 100%). Statistical differences in cell survival and cell viability were determined by two-way ANOVA followed by Bonferroni test.

DNA fiber assay

For experiments with cisplatin treatment or UVC irradiation, exponentially growing cells were pulse-labeled with 20 μM IdU (5-Iodo-2'-deoxyuridine, Millipore Sigma) for 20 min, washed twice with PBS, then pulse-labeled with 200 μM CldU (5-Chloro-2'-deoxyuridine, Millipore Sigma) for 1 h, followed by two washes with PBS. In the case of cisplatin treatment, CldU was added concomitantly with the indicated doses of cisplatin with or without mirin (50 μM). In the case of UVC irradiation, cells were irradiated immediately before addition of CldU. For co-treatment experiments with cisplatin and olaparib (Figures 6E and S5D), 10 μM olaparib was added 2 h prior to the DNA fiber assay and maintained in the cell media during the entire labeling period. For experiments with HU, cells were pulse-labeled with 20 μM IdU for 20 min, washed twice with PBS, then pulse-labeled with 200 μM CldU for 20 min followed by 2 h of treatment with 4 mM HU. Cells were harvested, pelleted at ~300 x g for 5 min at 4°C, and resuspended in PBS for a final concentration of 1,500 cells/μl.

For the DNA fiber assay with the ssDNA-specific S1 nuclease (S1 Fiber), cells were permeabilized with CSK100 (100 mM NaCl, 10 mM MOPS pH 7, 3 mM $MgCl_2$, 300 mM sucrose and 0.5% Triton X-100 in water) after the CldU pulse for 10 min at R.T., treated with the S1 nuclease (18001-016, ThermoFisher Scientific) at 20 U/mL in S1 buffer (30 mM sodium acetate pH 4.6, 10 mM zinc acetate, 5% glycerol, 50 mM NaCl in water) for 30 min at 37°C, and collected in PBS-0.1%BSA with cell scraper. Nuclei were then pelleted at ~4600 x g for 5 min at 4°C, then resuspended in PBS (nuclei cannot be quantified, so initial number of cells plated should be considered when resuspending to a final concentration of 1,500 nuclei/μl) (Quinet et al., 2017a; Quinet et al., 2016).

For both the standard DNA fiber assay and the S1 Fiber, 2 μL of cells were mixed with 6 μL of lysis buffer (200 mM Tris-HCl pH 7.5, 50 mM EDTA, 0.5% SDS in water) on top of a positively charged glass slide. After 5 min incubation at R.T., slides were tilted at a 20-45° angle to spread the fibers at a constant, low speed. After air drying for 10-15 min at R.T., DNA was fixed onto the slides with a freshly prepared solution of methanol: glacial acetic acid at 3:1 for 5 min, dried, then stored at 4°C for at least overnight.

For immuno-staining of DNA fibers, DNA was rehydrated in PBS twice for 5 min, then denatured with 2.5 M HCl for 1 h at R.T. Slides were then washed with PBS three times and blocked with 5% BSA at 37°C for 45 min-1 h. DNA fibers were immuno-stained with rat anti-BrdU (1/100, Ab6326, Abcam) and mouse-anti-BrdU (1/20, 347580, BD Biosciences) for 1.5 h at R.T., put in PBS, washed three times with PBS-0.1%Tween-20 for 5 min, then incubated with anti-rat Alexa Fluor 488 and anti-mouse Alexa Fluor 546 (1/100, A21470 and A21123, respectively, ThermoFisher Scientific) for 1 h at R.T. After three washes with PBS-0.1%Tween-20 of 5 min each, slides were put in PBS before mounting with Prolong Gold Antifade Reagent (P36930, ThermoFisher Scientific) (Quinet et al., 2017a).

Images were acquired with LAS AF software using TCS SP5 confocal microscope (Leica) with a 63x/1.4 oil immersion objective.

All the samples were blinded to the operators and each experiment was repeated at least two times independently. At least 10-15 images were taken across the whole slide using only one channel to select the regions for the images in order avoid any potential bias. At least 150-200 individual tracts were scored for each dataset. Only DNA fiber tracts where the beginning and end of each color was unambiguously defined were considered in the analysis. For all the DNA fiber experiments, we measured both IdU and CldU tracts only on forks characterized by contiguous IdU-CldU signals (i.e., progressing replication forks). The length of each tract was measured manually using the segmented line tool on ImageJ software (NIH). The pixel values were converted into μm using the scale bar generated by the microscope software. Size distribution of tract lengths or ratios from individual DNA fibers were plotted as scatter dot plot with the line representing the median. Data were pooled from independent experiments. Statistical differences in DNA fiber tract lengths were determined by Mann-Whitney test.

For the DNA fiber experiments with HU treatment, nascent DNA degradation was assessed by plotting the CldU/IdU ratio for each individual fiber. Decrease in the median of CldU/IdU distribution reflects degradation of the CldU tracts that were incorporated immediately prior to HU treatment (Lemaçon et al., 2017; Schlacher et al., 2011).

For the DNA fiber experiments with cisplatin or UVC, shortening of the first tract was measured only on forks characterized by contiguous IdU-CldU signals (and not on forks that have only the IdU label) to ensure that the shortening phenotype was indeed due to nucleolytic resection of stalled replication forks that can resume DNA synthesis and not to premature termination events (see also (Quinet et al., 2017a; Vindigni and Lopes, 2017)). Replication forks can quickly resume DNA synthesis within 15 min after

initial fork stalling and fork degradation does not significantly impact the timing of fork restart (Lemaçon et al., 2017; Schlacher et al., 2011). Consequently, individual forks might undergo multiple rounds of degradation and restart within the 60 min window of cisplatin treatment, and restart could be followed by a new round of degradation. Therefore, shortening of the IdU tracts on fibers with contiguous red and green tracts provides a readout of nascent DNA degradation of stalled replication forks that have been subsequently remodeled and restarted in a very dynamic process.

Of note, a recent report showed that fork reversal is a global mechanism of replication stress response, which is not restricted to sites of replication barriers (Mutreja et al., 2018). Our data do not provide exact information on the density of cisplatin-induced DNA lesions during the CldU labeling period. However, previous atomic absorption studies showed that treatment with 300 μM cisplatin for 60 min lead to 1.65 adducts/10 Kb in Chinese hamster ovary cells (Jones et al., 1991). Based on this published data, we could estimate that the number of cisplatin-induced DNA lesions when using 150 μM cisplatin for 60 min is approximately 1 adduct every 12 Kb. This is just a rough estimate and we cannot rule out the possibility that the number of cisplatin-induced DNA lesions might change depending on cell type. With these limitations in mind, having 1 adduct every for 12 Kb would mean that all the thymidine labeled replication forks have a high probability of encountering at least 1 adduct during the 60 min labeling period, as the DNA fiber tracts are approximately 30 Kb long after 60 min labeling with CldU (based on the fact that our DNA fiber tracts are approximately 10 μm long and that 1 μm corresponds to approximately 2.59 Kb of DNA according to (Jackson and Pombo, 1998)). In principle, these calculations would be compatible with a model in which fork processing occurs at sites of fork-stalling lesions. On the other hand, the extent of tract shortening observed upon treatment with HU (35%) and UVC (25%) is very similar to the one observed with cisplatin (approximately 30%), despite the different types of replication challenges. In particular, HU affects most replication forks by depriving cells from dNTP pools, whereas UVC and cisplatin induce DNA damage at specific sites. The fact that all three compounds lead to a similar extent of degradation argues in favor of the model that the cisplatin-induced effects detected by DNA fiber assays are due to genome-wide signaling. However, future studies will be necessary to properly address whether the cisplatin-induced effects described in this manuscript are due to genome-wide signaling.

RT-qPCR

Total RNA was extracted using the PureLink RNA mini Kit (12183018A, ThermoFisher Scientific), cDNA was synthesized by M-MLV Reverse Transcriptase (28025013, ThermoFisher Scientific) and PCR was performed using iQTM SYBR Green supermix (1708880, Biorad) by the CFX96 Real Time PCR Detection System (Biorad), according to the manufacturers' instructions (Lemaçon et al., 2017). The following primers were used: BRCA1: forward AGAAACCACCAAGGTCCAAAG, reverse GGGCCCATAGCAACAGATTT; PRIMPOL (Vallerga et al., 2015): forward TGTGGCTTTGGAGGTTACTGA, reverse TTCTACTGAAGTGCCGATACTGT; POLH (Christmann et al., 2016): forward ATCTTCTACTGGCACAAG, reverse ACATTATCTCCATCACTTCA; REV1 (Quinet et al., 2016) forward CCCAGACATCAGAGCTGTATAAT, reverse CTTCCTGTGCCTCTGTTACTT; REV3L: forward TCATGAGAAGGAAAG ACACTTTATG, reverse GCTGTAGGAGGTAGGGAATATG; BRCA2 (Lemaçon et al., 2017): forward AGGACTTGCCCCTTTCGTCTA, reverse TGCAGCAATTAACATATGAGG; SMARCAL1: forward CAGAGGCAGACCTTTCTGAAG, reverse CGGCCTCCTTTGGCTATG

ACTIN: forward CTCGCCTTTGCCGATCC, reverse ATGCCGGAGCCGTTGTC was used as an endogenous control. The results were calculated according to the 2-ΔΔCt methodology and are shown as relative expressions to the correspondent control. Statistical differences in mRNA levels were determined by two-way ANOVA followed by Bonferroni test.

Chromatin isolation and Western Blot

For total and phosphorylated proteins detection by western blot, total protein was extracted with lysis buffer (50 mM Tris-HCl pH 7.5, 20 mM NaCl, 1 mM $MgCl_2$, 0.1% SDS, 1X protease inhibitor, 1X phosSTOP) and benzonase (71206, Novagen) at 250 U/mL for 20 min on ice. Total protein concentration was measured using Pierce BCA protein assay kit (23227, ThermoFisher Scientific) according to the manufacturer's instructions. 1X NuPAGE LDS sample buffer (NP0007, ThermoFisher Scientific) and 200 mM DTT were added and proteins were denaturated at 100°C for 5 min. 10-30 μg proteins were loaded onto a NuPAGE Novex 4%–12% Bis-Tris Gel (NP0322BOX, ThermoFisher Scientific) and run with 1X NuPAGE MES SDS Running buffer (NP0002, ThermoFisher Scientific). Proteins were transferred onto a 0.45 μm pore nitrocellulose membrane (10600002, GE Healthcare Life Sciences) by cold wet-transfer in 1X Tris/Glycine Buffer (1610734, Biorad) and 20% Methanol at constant 400 mA for 45 min. Membranes were blocked with 5% milk (170-6404, Biorad) in TBS-0.1% Tween-20 for total proteins or with 5% BSA in TBS for phosphorylated proteins for 1 h at R.T. The following primary antibodies were used: PRIMPOL (1/1,000; (Mourón et al., 2013)), RAD51 (1/1,000, H-92, Santa Cruz), BRCA1 (1/200, OP-93, Millipore Sigma), Chk1 (1/1,000, G4, Santa Cruz), p-Chk1 Ser345 (1/1,000, 2348, Cell Signaling), RPA (1/5,000, ab2175, Abcam), p-RPA Ser33 (1/5,000, A300-246A, Bethyl), γH2AX (1/1,000, 05-636, Millipore Sigma), H2AX (1/1,000, 2595, Cell Signaling), V5 (1/5,000, R96025, ThermoFisher Scientific), PCNA (1/2,000, 25280, Santa Cruz and ab29, Abcam), GAPDH (1/20,000, ab181602, Abcam), SMARCAL1 (1/500, E-12, Santa Cruz), TUBULIN (1/5,000, T5168, Millipore Sigma), BRCA2 (1/1,000, OP-95, Calbiochem), SMC1 (Kojic et al., 2018). IRDye Infrared secondary antibodies from LI-COR were used and proteins were detected by Odyssey CLx (1/20,000, LI-COR). Alternatively, HRP-conjugated antibodies were used, and proteins were detected using ECL (1/5,000, 32106, Pierce) according to the manufacturer's instruction. When indicated, chromatin was isolated using biochemical fractionation, as described (Méndez and Stillman, 2000). Statistical differences in protein levels were determined by two-way ANOVA followed by Bonferroni test.

Electron microscopy

For the EM analysis of replication intermediates, 5-10x10^6 U2OS or UW cells were harvested immediately after treatment with the cisplatin challenging dose (150 μM for 1 h) with or without the pre-dose. For experiments with the MRE11 inhibitor mirin, mirin was added for 4 h and cisplatin was added during the last h of mirin treatment. Genomic DNA was cross-linked by three rounds of incubation in 10 μg/mL 4,5′,8-trimethylpsoralen (Sigma-Aldrich) and 3 min of irradiation with 366 nm UV light on a precooled metal block (Lemaçon et al., 2017; Thangavel et al., 2015). Cells were lysed and genomic DNA was isolated from the nuclei by proteinase K (Roche) digestion and phenol-chloroform extraction. DNA was purified by isopropanol precipitation, digested with PvuII HF in the proper buffer for 3–5 h at 37°C and replication intermediates were enriched on a benzoylated naphthoylated DEAE–cellulose (Sigma-Aldrich) column. EM samples were prepared by spreading the DNA on carbon-coated grids in the presence of benzyl-dimethyl-alkylammonium chloride and visualized by platinum rotary shadowing. Images were acquired on a transmission electron microscope (JEOL 1200 EX) with side-mounted camera (AMTXR41 supported by AMT software v601) and analyzed with ImageJ (NIH). EM analysis allows distinguishing duplex DNA—which is expected to appear as a 10 nm thick fiber after the platinum/carbon coating step necessary for EM visualization—from ssDNA, which has a reduced thickness of 5-7 nm. Internal ssDNA gaps behind forks are scored by measuring ssDNA regions located in the daughter arms of three-way junction fork structures, and excluding ssDNA discontinuities present at fork junctions. The criteria used for the unequivocal assignment of reversed forks include the presence of a rhomboid structure at the junction itself in order to provide a clear indication that the junction is opened up and that the four-way junction structure is not simply the result of the occasional crossing of two DNA molecules (Neelsen et al., 2014). In addition, the length of the two arms corresponding to the newly replicated duplex should be equal (b = c), whereas the length of the parental arm and the regressed arm can vary ($a \neq b = c \neq d$). Conversely, canonical Holliday junction structures will be characterized by arms of equal length (a = b, c = d). Statistical differences in the frequency of reversed forks or forks with internal gaps were determined by two-way ANOVA followed by Bonferroni test for grouped analysis or by unpaired t test between two groups only.

Doubling time experiments in DT40 cells

DT40 cell culture, doubling time measurement and genetic manipulation were performed as previously described (Simpson and Sale, 2003). To generate *smarcal1* mutants in wild-type and *primpol* −/− DT40 cells (Schiavone et al., 2016; Šviković et al., 2019), previously published constructs replacing a 25 kb of the gene body with bsr or puror selectable markers were used (Keka et al., 2015). Successful targeting was confirmed by PCR using primer pairs amplifying across the 5′ (gene specific: TGTTTAACTTGAAA ACAAAGGGAGG; Bsr specific: GAACTCATTCCACTCAAATATACCC) or 3′ homology arms (gene specific: AAATATGAGAG CTAGTTTGTGC; puro specific: ATAAATACAAAATTGGGGGTGG). Heterozygosity was confirmed by amplifying the polymorphic region between the homology regions using TAAAAAATGGACGTTTACTTCTTGCGGATG and AATAACTACTTGGAAAGTGCTCT TGAGTTG primers and analyzed by Sanger sequencing. RT-qPCR was performed using TAAAAAATGGACGTTTACTTCTTGC GGATG and AATAACTACTTGGAAAGTGCTCTTGAGTTG, or TTACGTGGGCAGAGGCATTT and TGCTGAGGAGGTCAAAGCTG primer pairs, and normalized to expression of eF1α (primers: GGTTATGCCCCTGTGCTGGATT and CTTCTTGTCGACGGCCT TGATGA (Romanello et al., 2016)). Statistical differences in doubling time of DT40 cell mutants were assessed by Kruskal-Wallis test.

Cell cycle analysis

Cell Cycle analysis was performed with Click-iT EdU Alexa Fluor 488 Imaging Kit (C10337, Thermo Fisher Scientific), according to the manufacturer's instructions. For pulse experiments, asynchronous cells were treated with 10 μM 5-ethynyl-2'-deoxyuridine (EdU, E10187, Thermo Fisher Scientific) for 30 min and collected immediately after. For the pulse chase experiments, cells were treated with 10 μM EdU for 30 min, washed twice with PBS, supplemented with fresh cell growth media, and collected after 24 h. Cells were fixed with 3.7% formaldehyde for 10 min at R.T., and blocked with 1% BSA in PBS for 10 min, before permeabilization in 1% BSA with 0.5% saponin (8047-15-2, Millipore Sigma) in the dark for 30 min. Permeabilized cells were then incubated with the Click-iT cocktail in the dark for 30 min before staining with DAPI (1% BSA, 0.1 mg/mL RNase A, 2 μg/mL DAPI) for 20 min in the dark at R.T. Samples were run through flow cytometry (FACSCanto II, BD Biosciences) and data were analyzed on FlowJo. EdU versus DAPI dot plots and histograms of DNA content of EdU-positive cells were plotted in order to visualize the distribution in the cell cycle of S-phase cells.

Immunofluorescence

For immunodetection of cisplatin-modified DNA (Pt-DNA), cells were plated onto glass coverslips and allowed to fully attach overnight. After treatment with cisplatin, cells were fixed with 4% paraformaldehyde for 10 min at R.T. Cells were then washed twice with PBS before permeabilization with 0.5% Triton X-100 for 10 min at R.T. Following permeabilization, coverslips were washed 3 times with PBS, DNA was denatured with 2 M HCL for 10 min at 37°C, and cells were then washed 3 times in PBS. Coverslips were blocked in 5% BSA with 0.05% Tween 20 in PBS (T-BSA) for 1 h at R.T and were then were incubated with primary antibody, anti-Pt-DNA (ab103261, abcam; (Jazaeri et al., 2013; Rocha et al., 2014; Tilby et al., 1991)) diluted 1/5,000 in T-BSA overnight at 4°C. After incubation, coverslips were washed 3 times in PBS before incubation with secondary antibody anti-rat Alexa Fluor 488 diluted 1/1,000 in T-BSA (A21470, Thermo Fisher Scientific). Cells were washed 3 times in PBS, incubated with 0.05 μg/mL DAPI in PBS for 10 min at R.T., washed again 3 times in PBS, and then mounted onto a glass slide with ProLong Gold Antifade Reagent (P36930, Thermo Fisher Scientific). Slides were allowed to dry overnight at R.T. in the dark before visualization. Images were acquired with LAS AF software

using TCS SP5 confocal microscope (Leica) with a 63x/1.4 oil immersion objective. The integrated density of cisplatin-modified DNA (Pt-DNA) signal *per* nucleus was measured using the ImageJ software (NIH). To avoid bias, a macro program was used to identify nuclei outlines based on DAPI staining and consistently measure cisplatin-modified DNA signal only in the nuclei. At least 100 nuclei were scored per data and three independent experiments were performed. Size distribution of Pt-DNA signal from individual nuclei were plotted as a scatter dot plot with the line representing the median. Data were pooled from independent experiments. Statistical differences in Pt-DNA signal were determined by Mann-Whitney test.

Chromatin-bound RPA32 detection by flow cytometry

Detection of chromatin-bound RPA32 in UW and UW+BRCA1 cells was adapted from previously published flow cytometry protocols (Forment and Jackson, 2015; Quinet et al., 2014). Cells were first collected and resuspended in CSK100 buffer (100 mM NaCl, 10 mM MOPS pH 7, 3 mM $MgCl_2$, 300 mM sucrose and 0.5% Triton X-100) on ice for 10 min to extract non-chromatin-bound proteins. Some cells were set apart to act as the non-extracted control. Cells were then washed with PBS-B (0.1% BSA in PBS) before fixing cells in 2% paraformaldehyde for 15 min at R.T. Cells then pelleted at 1500 x g for 5-10 min at 4°C and washed with PBS-B before blocking and permeabilization in BSA-T (1% BSA, 0.2% Triton X-100 in PBS) for 5 min at R.T. Cells were incubated with primary antibody anti-RPA32 (ab2175, Abcam) diluted at 1/500 in BSA-T for 2 h at R.T. with gentle shaking. Cells were then washed with BSA-T before incubation with secondary antibody anti-mouse Alexa Fluor 594 (A11005, Thermo Fisher Scientific) diluted 1/1,000 in BSA-T for 1 h in the dark with gentle shaking. After secondary incubation, cells were washed once with BSA-T, and then resuspended in DAPI solution (1% BSA, 0.1 mg/mL RNase A, 2 μg/mL DAPI) for 20 min in the dark at R.T. Samples were kept at 4°C until processing. Samples were run through flow cytometry (FACSCanto II, BD Biosciences) and data were analyzed on FlowJo. The gate for the positive signal for RPA32 staining was defined based on the non-extracted control sample in which cells were not subjected to pre-extraction prior to fixation and therefore are virtually all positive for RPA32 staining. Next, the same gate was applied to all the samples in which soluble proteins were pre-extracted before fixation in order to specifically detect cells positive for chromatin-bound RPA32. See Figure S3A for a representative experiment. Statistical differences in percentage of cells positive for chromatin-bound RPA were assessed by two-way ANOVA followed by Bonferroni test.

QUANTIFICATION AND STATISTICAL ANALYSIS

Statistical analysis was performed using Prism (GraphPad Software). In all cases: *ns*, non-significant, * $p < 0.05$, ** $p < 0.01$, *** $p < 0.001$, **** $p < 0.0001$.

Statistical differences in DNA fiber tract lengths were determined by Mann-Whitney test. Statistical differences for all grouped analyses, i.e., cell survival, cell count, mRNA (RT-qPCR) and protein (western blot) levels, percentage of cells positive for chromatin-bound RPA (flow cytometry), frequency of fork reversal and forks with internal gaps (EM) shown in Figures 4D and 5B, respectively, were assessed by two-way ANOVA followed by Bonferroni test. Statistical differences in frequency of fork reversal and forks with internal gaps (EM) shown in Figures 5C and 5D were determined by unpaired t test. Statistical differences in doubling time of DT40 cell mutants were assessed by Kruskal-Wallis test.

DATA AND CODE AVAILABILITY

All data are available by request. Raw images have been deposited in Mendeley Data and are available at https://doi.org/10.17632/c9dn6snk6w.1.

Molecular Cell, Volume 77

Supplemental Information

PRIMPOL-Mediated Adaptive Response

Suppresses Replication Fork

Reversal in BRCA-Deficient Cells

Annabel Quinet, Stephanie Tirman, Jessica Jackson, Saša Šviković, Delphine Lemaçon, Denisse Carvajal-Maldonado, Daniel González-Acosta, Alexandre T. Vessoni, Emily Cybulla, Matthew Wood, Steven Tavis, Luis F.Z. Batista, Juan Méndez, Julian E. Sale, and Alessandro Vindigni

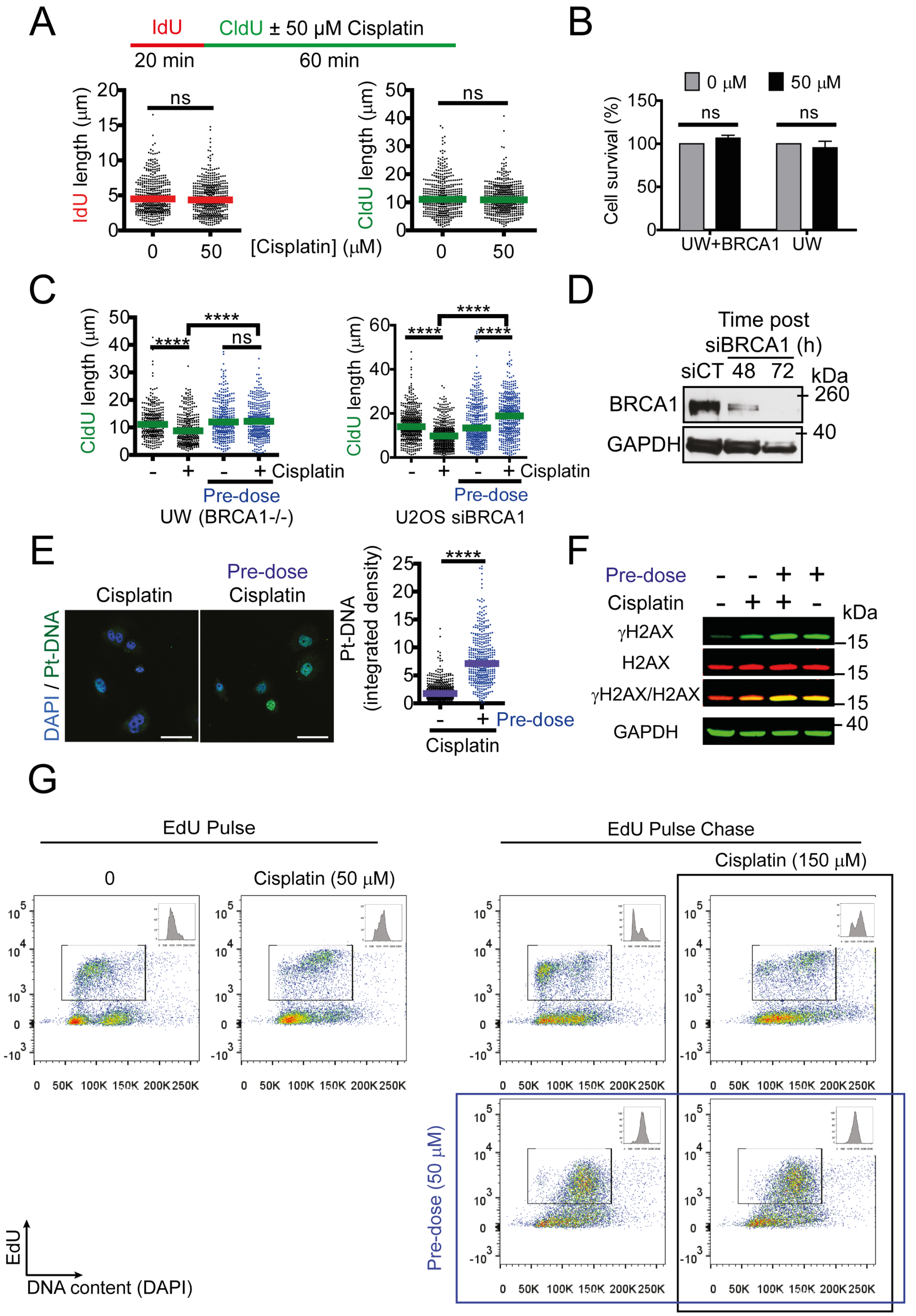

Figure S1. Treatment with a cisplatin pre-dose abolishes nascent DNA degradation in BRCA1-deficient cells, related to Figure 1. (A) Schematic of the single-molecule

DNA fiber tract analysis (top) and size distribution of IdU (bottom left) and CldU (bottom right) tract length in UW cells ± 50 μM cisplatin. Data are pooled from three independent experiments. N ≥ 150 tracts for each data set. Bars represent the median. Statistics: Mann-Whitney; *ns*, non-significant. (B) Cell survival of UW+BRCA1 and UW cells 24 hours after treatment with 50 μM cisplatin (pre-dose). Data represent means ± s.e.m. from three experiments. Statistics: two-way ANOVA followed by Bonferroni test; *ns*, non-significant. (C) Size distribution of CldU tract length in UW cells (left) and U2OS cells depleted for BRCA1 (siBRCA1) (right) ± 150 μM cisplatin with or without the pre-dose. Data are pooled from three independent experiments. N ≥ 150 tracts for each data set. Bars represent the median. Statistics: Mann-Whitney; **** $P < 0.0001$. (D) Expression of BRCA1 48 and 72 hours after siRNA (siBRCA1) knockdown in U2OS cells. (E) Immunofluorescence for cisplatin-modified DNA (Pt-DNA) in UW cells treated with 150 μM cisplatin for 1 hour with or without the pre-dose. Representative images are shown on the left (scale bar: 50 μm). The distribution of the integrated density of cisplatin-modified DNA staining *per* nuclei measured with ImageJ is represented as scatter dot plots (right). Data are pooled from three independent experiments. Bars correspond to median. N ≥ 100 nuclei for each data set. Statistics: Mann-Whitney; **** $P < 0.0001$. (F) Phosphorylated H2AX (γH2AX, green) and total H2AX (red) in UW cells 24 hours after treatment with 150 μM cisplatin with or without the pre-dose. γH2AX/H2AX shows simultaneous detection of both bands. GAPDH was used a loading control. A western blot representative of three independent experiments is shown. (G) UW cells were pulse labeled with EdU for 30 min and immediately fixed 24 hours after treatment with 0 or 50 μM cisplatin (Left, EdU pulse). UW cells were pulse labeled with EdU for 30 minutes immediately before treatment with 150 μM cisplatin (with or without the 50 μM cisplatin pre-dose) and fixed 24 hours later (Right, EdU pulse chase). This pulse chase approach allows evaluation of cell cycle progression of the cells that were replicating at the time of the cisplatin treatment. At the top right of each panel, the cell cycle distribution of EdU-positive cells as determined by DAPI is shown.

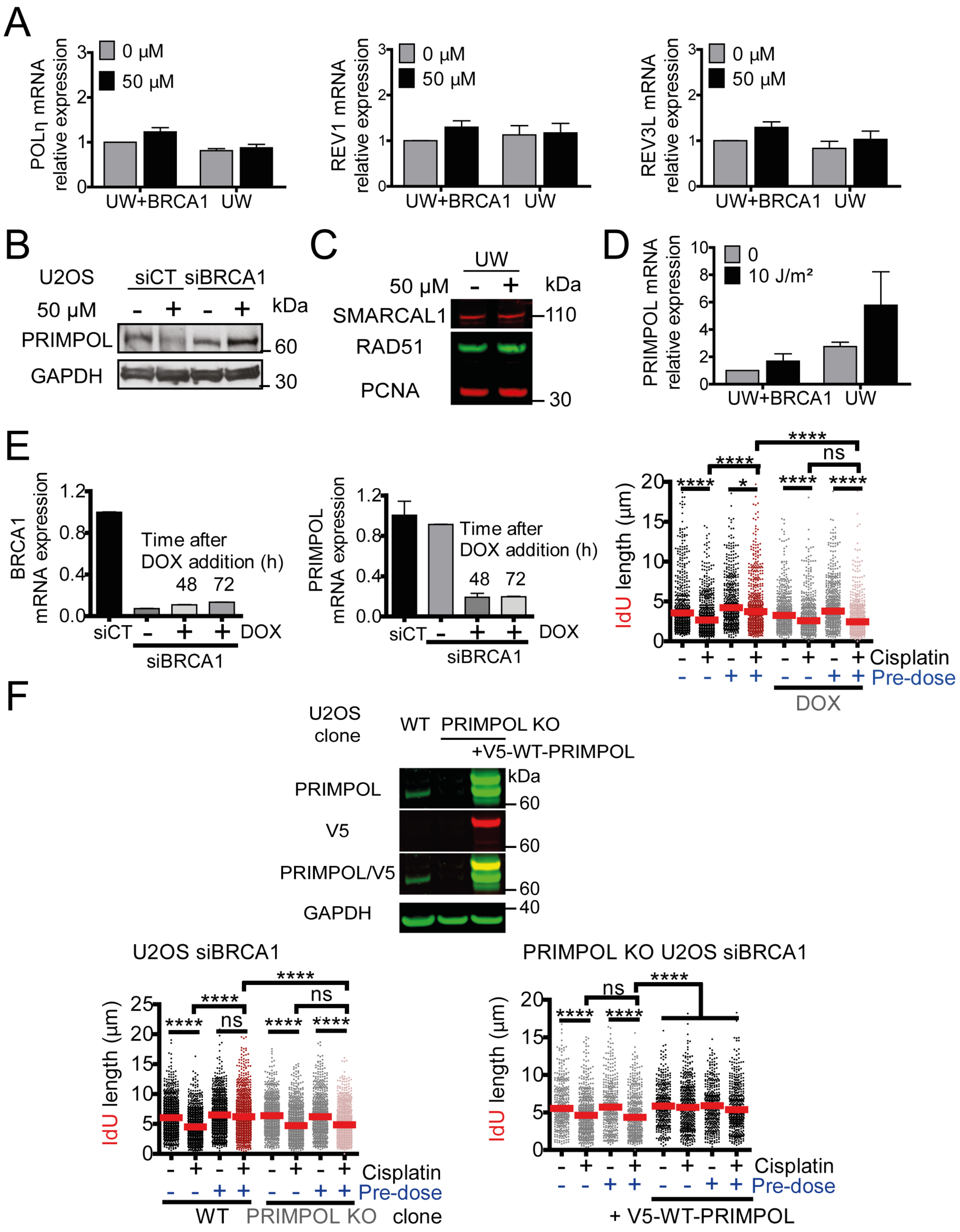

Figure S2. PRIMPOL, but not POLη, REV1 and REV3L, is upregulated in BRCA1-deficient cells, related to Figure 2. (A) TLS polymerases POLη, REV1 and REV3L

(catalytic sub-unit of POLζ) mRNA expression 24 hours after treatment with 0 or 50 μM cisplatin in UW and UW+BRCA1 cells. Data represent means ± s.e.m. from three independent experiments and are presented as relative to untreated UW+BRCA1 cells. (B) PRIMPOL protein expression 24 hours after treatment with 0 or 50 μM cisplatin in U2OS cells depleted for BRCA1 (siBRCA1) or transfected with a control siRNA (siCT). A representative western blot from three independent experiments is shown. GAPDH was used as a loading control. (C) SMARCAL1 and RAD51 protein expression in UW cells 24 hours after treatment with 0 or 50 μM cisplatin. PCNA was used as a loading control. (D) PRIMPOL mRNA expression 24 hours after 0 or 10 J/m^2 UVC in UW and UW+BRCA1 cells. Data represent means ± s.e.m. from three independent experiments and are presented as relative to untreated UW+BRCA1 cells. (E) Validation by RT-qPCR of BRCA1 and PRIMPOL depletion 48 (time of pre-dose) and 72 (time of DNA fiber assay) hours after transfection with siBRCA1 and addition of doxycycline to U2OS cells stably expressing a doxycycline-inducible shPRIMPOL (Left).) Size distribution of IdU tract length in doxycycline-inducible shPRIMPOL U2OS cells depleted for BRCA1 (siBRCA1) ± 150 μM cisplatin with or without the pre-dose, in the presence or absence of doxycycline (DOX) (Right. Data are pooled from three independent experiments. n ≥ 150 tracts for each data set. Bars represent the median. Statistics: Mann-Whitney; **** $P < 0.0001$. (F) PRIMPOL protein expression in PRIMPOL KO and wild-type counterpart U2OS clones and in the PRIMPOL KO clone expressing an exogenous V5-tagged WT-PRIMPOL (PRIMPOL in green, V5 in red, PRIMPOL/V5 shows simultaneous detection of both bands. GAPDH was used a loading control (Top). Size distribution of IdU tract length in PRIMPOL KO and wild-type counterpart U2OS clones depleted for BRCA1 ± 150 μM cisplatin with or without pre-dose (bottom left). Size distribution of IdU tract length in the PRIMPOL KO U2OS clone ± exogenous V5-tagged WT-PRIMPOL ± 150 μM cisplatin with or without pre-dose (bottom right). Data are pooled from three (bottom left) and two (bottom right) independent experiments. N ≥ 150 tracts for each data set. Bars represent the median. Statistics: Mann-Whitney; **** $P < 0.0001$

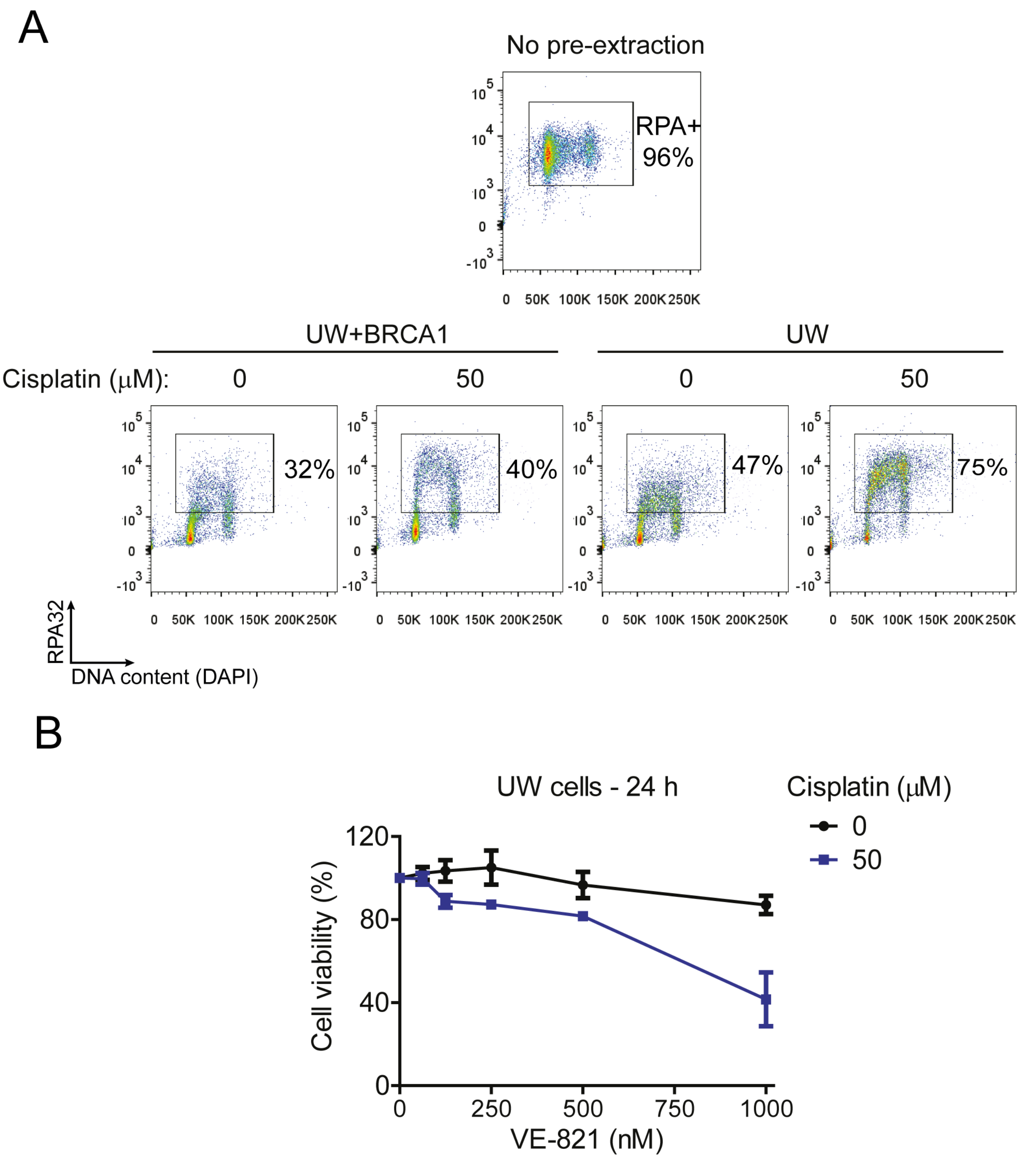

Figure S3. Impact of ATRi on cell survival of UW cells treated with cisplatin, related to Figure 3. (A) Representative experiment showing the detection of chromatin-bound RPA32 as a function of DNA content by flow cytometry in UW+BRCA and UW cells 24 hours after treatment with 50 μM cisplatin. The gate for the positive signal for RPA (RPA+) was defined based on a control sample in which cells were not subjected to pre-extraction prior to fixation and therefore are nearly all positive for RPA staining. Next, the same gate was applied to all the samples in which soluble proteins were pre-extracted before fixation in order to specifically detect cells positive for chromatin-bound RPA. Quantification from

three independent experiments is shown in Figure 3A. (B) Cell survival assessed by XTT of UW cells 24 hours after treatment with 50 μM cisplatin for 1 hour with or without the indicated doses of VE-821. VE-821 was added 1 hour prior to treatment with cisplatin and kept in the media for the following 24 hours. Data are expressed as relative to untreated control (without VE-821) and correspond to mean ± s.e.m. of four independent experiments.

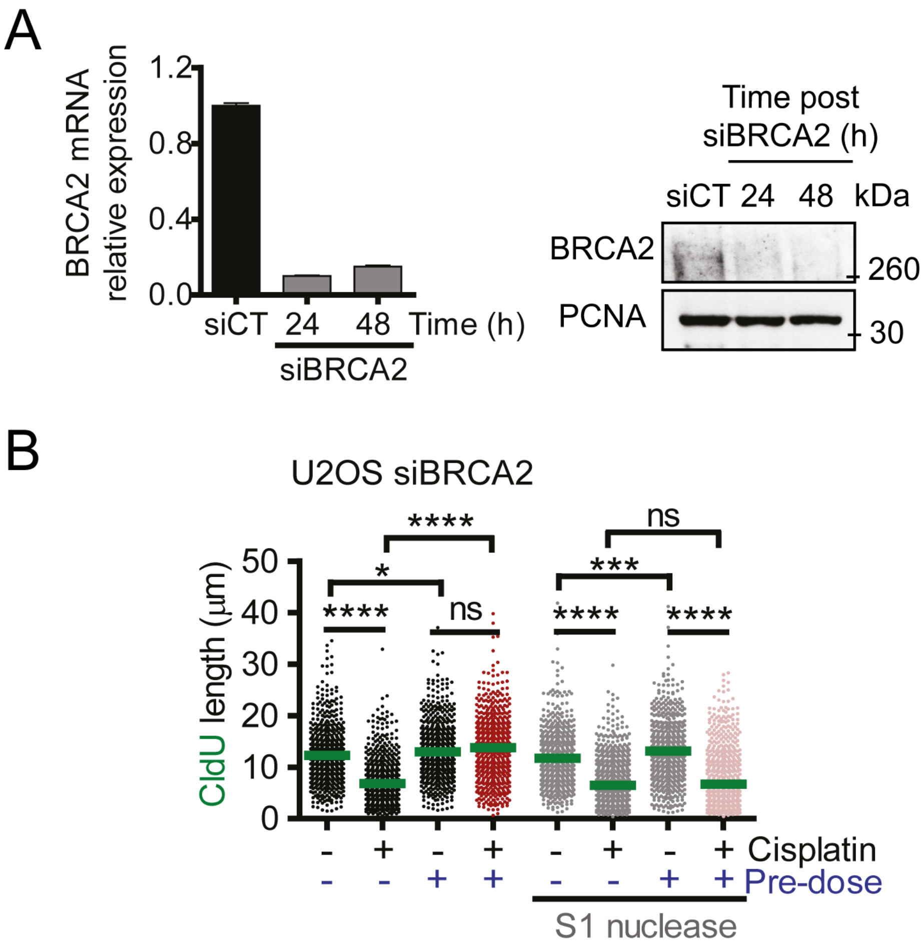

Figure S4. Treatment with a cisplatin pre-dose abolishes nascent DNA degradation and leads to ssDNA gaps in BRCA2-deficient cells, related to Figure 4. (A) Validation of BRCA2 depletion with siRNA (siBRCA2) in U2OS cells by RT-qPCR (left) and Western blot (right) 24 (pre-dose time) and 48 (DNA fiber assay time) hours after transfection. (B) Size distribution of CldU tract length in U2OS cells depleted for BRCA2 ± 150 µM cisplatin with or without the pre-dose followed by treatment with S1 nuclease or not. Data are pooled from three independent experiments. N ≥ 150 tracts for each data set. Bars represent the median. Statistics: Mann-Whitney; **** $P < 0.0001$.

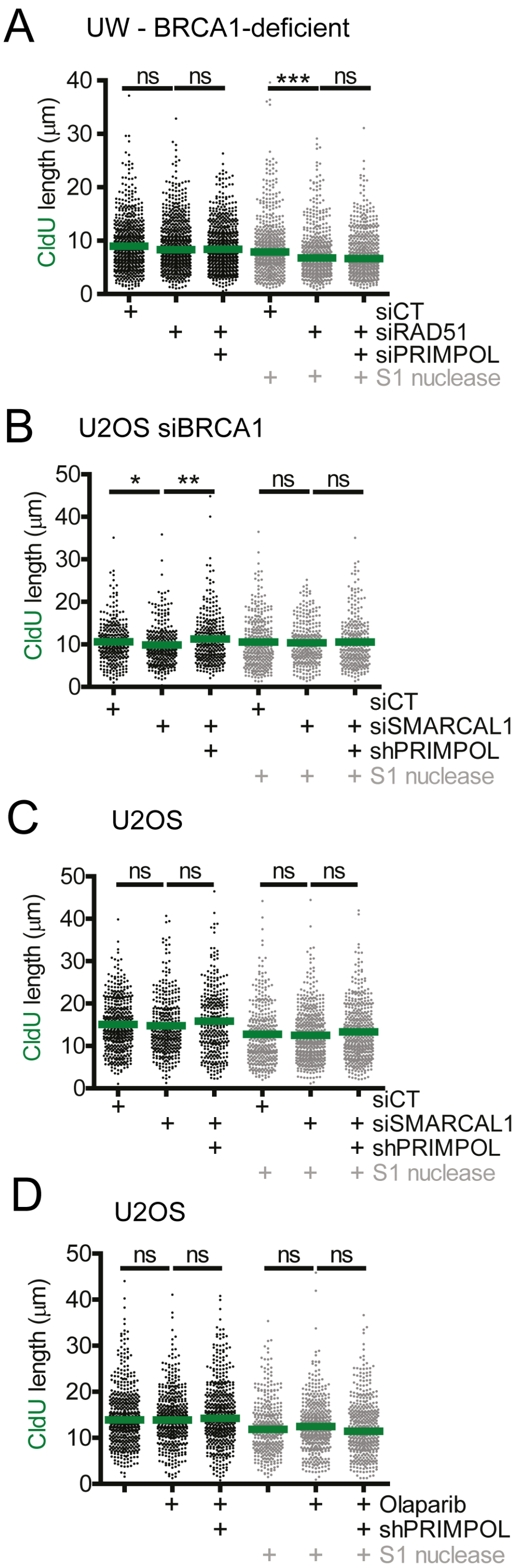

Figure S5. DNA fiber assay data of untreated samples, related to Figure 6. (A) Untreated samples from DNA fiber data shown in Figure 6B. (B) Untreated samples from

DNA fiber data shown in Figure 6C. (C) Untreated samples from DNA fiber data shown in Figure 6D. (D) Untreated samples from DNA fiber data shown in Figure 6E. Statistics: Mann-Whitney; *ns*, non-significant, * $P < 0.05$, ** $P < 0.01$, *** $P < 0.001$.

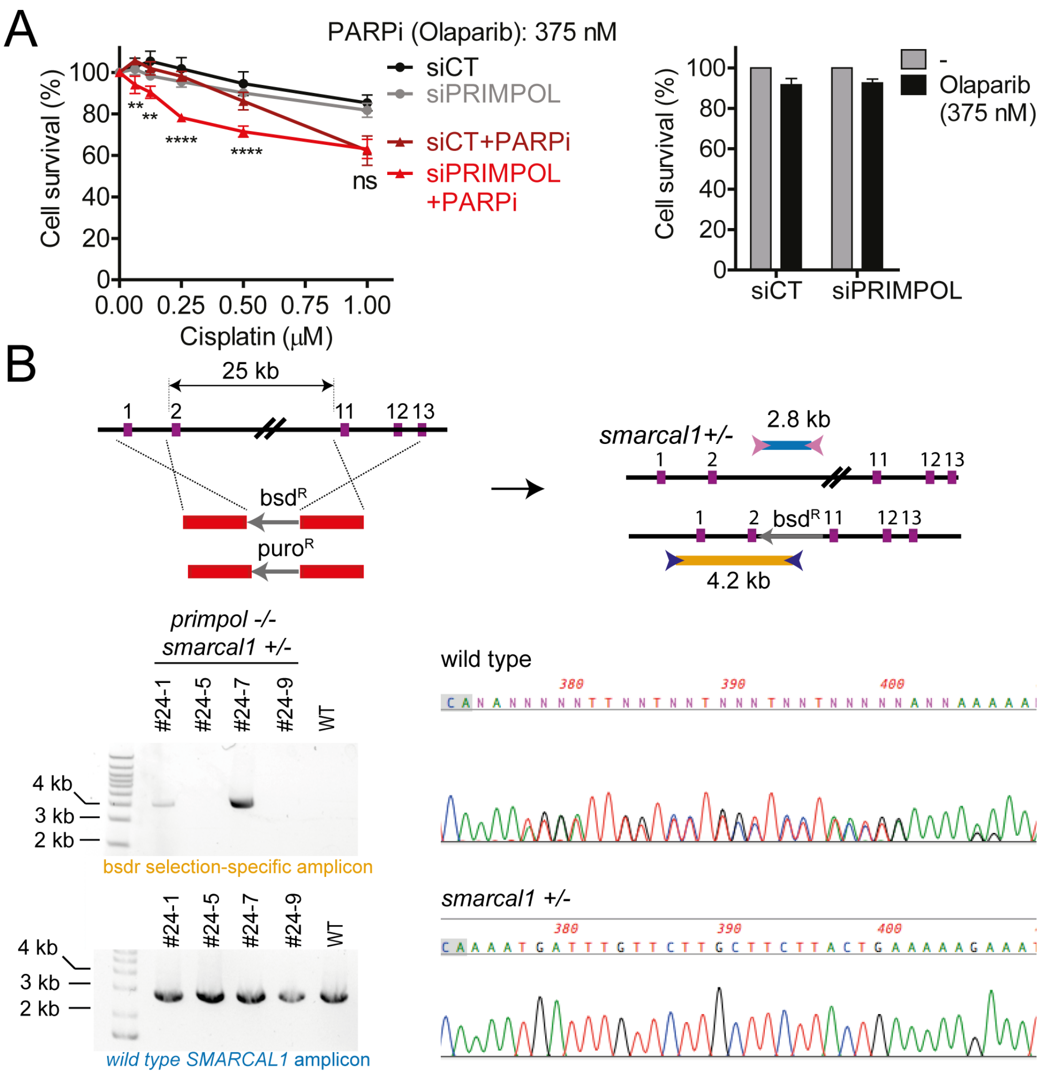

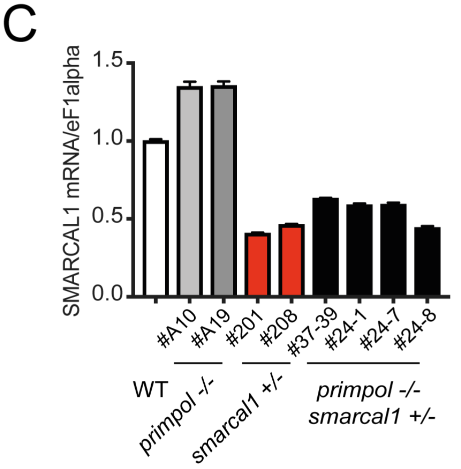

D

cell line	no. passages	doubling time [h] (mean ± SD)
wild type	10	10.81 ± 0.21
	20	11.52 ± 1.40
	40	10.82 ± 1.56
primpol -/-	10	12.89 ± 0.55
	20	13.41 ± 1.74
	40	13.76 ± 3.04
smarcal1 +/-	10	10.93 ± 0.94
	20	11.88 ± 1.83
	40	12.22 ± 3.27
primpol -/- smarcal1+/-	10	19.94 ± 3.91
	20	16.31 ± 5.10
	40	14.24 ± 3.28

Figure S6. Impact of PRIMPOL on cell survival, related to Figure 7. (A) Cell survival of U2OS cells transfected with siCT or siPRIMPOL upon 4 days of chronic treatment with

the indicated doses of cisplatin and PARP inhibitor (PARPi, Olaparib, 375 nM) (left). Cell survival of U2OS cells transfected with siCT or siPRIMPOL 4 days after treatment with Olaparib (375 nM) only (right). Data represent means ± s.e.m. from four independent experiments. Statistics: two-way ANOVA followed by Bonferroni test comparing siCT+PARPi and siPRIMPOL+PARPi (*ns*, non-significant, ** $P < 0.01$, **** $P < 0.0001$). (B) *smarcal1* knockout strategy (Keka et al., 2015) in DT40 *primpol* mutants is depicted on the top, with representative genotyping analysis on the bottom left (bsd^r specific amplicon [orange] to confirm successful targeting and *smarcal1* amplicon [blue] to confirm heterozygosity). The bottom right panel is a Sanger sequencing traces of the polymorphic region located between the homology regions. The upper sequencing trace shows a polymorphic region of *smarcal1,* with the ambiguous peaks reflecting differences between the maternal and paternal alleles of the cell line, as DT40 is derived from an F1 hybrid strain of chicken. The bottom panel is from a *smarcal1+/-* line and has lost the ambiguous peak, indicative of heterozygous deletion. (C) The expression levels of *smarcal1* analyzed by RT-qPCR in several independently derived DT40 clones. Data represent mean ± SD of three qPCR replicates. (D) Measured doubling times of various DT40 mutants at different times relative to transfection with *smarcal1* targeting construct. Mean and standard deviation of three experiments reported. For reference, the original smarcal1-/- cell line (Keka et al., 2015) has a doubling time of 18.2 +/- 3.3 h compared with WT doubling time 11.6 +/- 1.5 h.

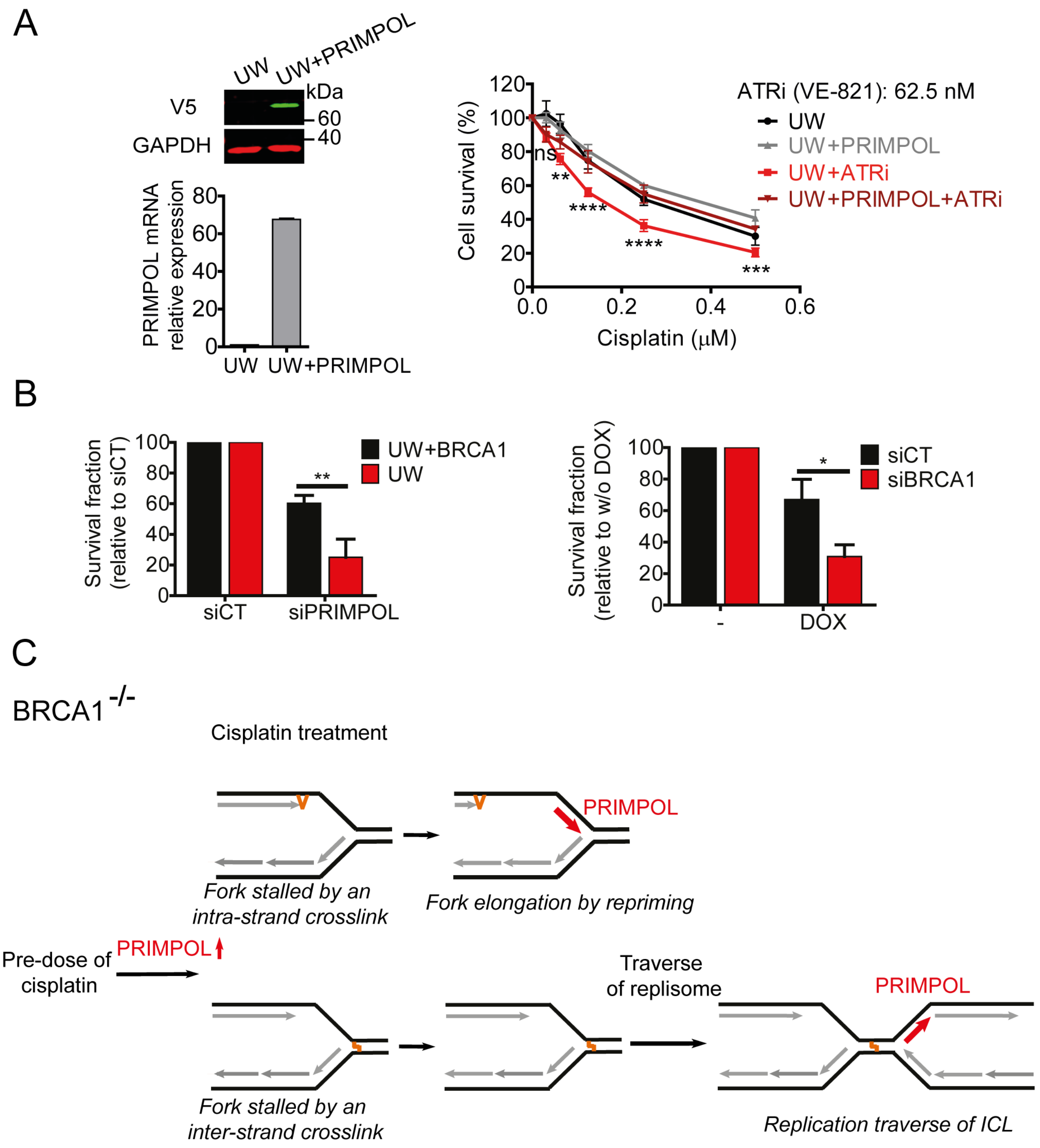

Figure S7: Impact of PRIMPOL on cell survival and proposed model for the role of PRIMPOL in the replication stress response to cisplatin-induced intra- and inter-strand crosslinks, related to Figure 7. (A) Validation of the generation of UW+PRIMPOL cells (UW cells stably overexpressing V5-WT-PRIMPOL) by detection of the V5 tag by western blot (top left) and by PRIMPOL mRNA levels as detected by RT-qPCR (bottom right). Cell survival of UW and UW+PRIMPOL cells 6 days upon chronic

treatment with ATR inhibitor (ATRi, VE-821, 62.5 nM) and the indicated doses of cisplatin (Right). Data represent means ± s.e.m. from three independent experiments. Statistics: two-way ANOVA followed by Bonferroni test comparing UW+ATRi *versus* UW+PRIMPOL+ATRi (*ns*, non-significant, * $P < 0.05$, ** $P < 0.01$, *** $P < 0.001$, **** $P < 0.0001$). (B) Colony forming assay in UW and UW±BRCA1 cells depleted for PRIMPOL (siPRIMPOL) (Left). Colony forming assay in PRIMPOL-depleted (DOX) in U2OS cells depleted for BRCA1 (siBRCA1) or treated with siRNA control (siCT) (Right). Data represent mean ± s.e.m. of three and two independent experiments, respectively. Statistics: two-way ANOVA followed by Bonferroni test comparing BRCA-proficient *versus* BRCA1-deficient. (* $P < 0.05$, ** $P < 0.01$). (C) Proposed model for the role of PRIMPOL in the replication stress response to cisplatin-induced intra- (top) and inter-strand crosslinks (bottom).

A

UW cells				Pre-dose		
% Forks with 1, 2, ≥ 3 internal gaps<br><br>(Total molecules analyzed)	0	Cisplatin	Cisplatin + Mirin	0	Cisplatin	Cisplatin + Mirin
Exp #1	-	12, 5, 0<br><br>(110)	14, 3, 0<br><br>(99)	-	15, 9, 6<br><br>(83)	11, 1, 0<br><br>(97)
Exp #2	11, 1, 0<br><br>(76)	12, 4, 0<br><br>(74)	10, 0, 0<br><br>(76)	13, 0, 0<br><br>(77)	17, 3, 3<br><br>(72)	11, 1, 0<br><br>(74)
Exp #3	-	12, 0, 0<br><br>(95)	9, 3, 1<br><br>(79)	-	21, 3, 3<br><br>(95)	7, 0, 0<br><br>(81)

B

UW cells				Pre-dose		
% Reversed Forks<br><br>(Total molecules analyzed)	0	Cisplatin	Cisplatin + Mirin	0	Cisplatin	Cisplatin + Mirin
Exp #1	-	11<br><br>(110)	18<br><br>(99)	-	7<br><br>(83)	19<br><br>(97)
Exp #2	7<br><br>(76)	9<br><br>(74)	16<br><br>(76)	8<br><br>(77)	8<br><br>(72)	16<br><br>(74)
Exp #3	-	12.6<br><br>(95)	22.8<br><br>(79)	-	12.6<br><br>(95)	19.8<br><br>(81)

Table S1. Electron microscopy data, related to Figures 4 and 5. (A) Percentage of replication forks with 1, 2 or ≥ 3 ssDNA gaps behind forks observed in three independent EM experiments for samples in Figure 4D. (B) Percentage of reversed forks observed in three independent EM experiments for samples in Figure 5B. Number of total molecules analyzed per sample is indicated in brackets.

A

U-2 OS cells		Pre-dose		
% Reversed Forks (Total molecules analyzed)		Cisplatin		Cisplatin
Exp #1		24.1 (54)		15.5 (71)
Exp #2	5.8 (87)	18 (89)	10 (80)	12.8 (78)
Exp #3		23.4 (64)		15.8 (76)

B

U-2 OS cells		Pre-dose		
% Forks with internal gaps (Total molecules analyzed)		Cisplatin		Cisplatin
Exp #1		11.1 (54)		15.5 (71)
Exp #2	8.05 (87)	6.7 (89)	7.5 (80)	15.4 (78)
Exp #3		10.9 (64)		17.1 (76)

Table S2. Electron microscopy data, related to Figure 5. (A) Percentage of reversed forks observed in three independent EM experiments for samples in Figure 5C left. (B) Percentage of replication forks with ssDNA gaps behind forks observed in three independent EM experiments for samples in Figure 5C right. Number of total molecules analyzed per sample is indicated in brackets.

A

U-2 OS cells		V5-PRIMPOL
% Reversed Forks (Total molecules analyzed)	Cisplatin	Cisplatin
Exp #1	16.5 (85)	4.1 (98)
Exp #2	18.6 (86)	5.3 (95)
Exp #3	25 (76)	8.1 (86)

B

U-2 OS cells		V5-PRIMPOL
% Forks with internal gaps (Total molecules analyzed)	Cisplatin	Cisplatin
Exp #1	7.1 (85)	19.4 (98)
Exp #2	8.1 (86)	22.1 (95)
Exp #3	10.5 (76)	18.6 (86)

Table S3. Electron microscopy data, related to Figure 5. (A) Percentage of reversed forks observed in three independent EM experiments for samples in Figure 5C bottom left. (B) Percentage of replication forks with ssDNA gaps behind forks observed in three independent EM experiments for samples in Figure 5C bottom right. Number of total molecules analyzed per sample is indicated in brackets.
